# Molecular heterogeneity and clonal origin of CCR8^+^ effector regulatory T cells in human cancer

**DOI:** 10.1038/s41467-026-76670-6

**Published:** 2026-08-21

**Authors:** Julian Swatler, Simone Puccio, Emanuele Voulaz, Giuseppe Marulli, Matthew Wheeler, Sharmila Sambanthamoorthy, Eloise Scamardella, Simona Feno, Sabina Testa, Chiara Camisaschi, Valentina Errico, Matteo Simonelli, Agnese Losurdo, Emilia Maria Cristina Mazza, Enrico Lugli

**Affiliations:** 1 IRCCS Humanitas Research Hospital, Rozzano, Milan, Italy; 2 Institute of Genetic and Biomedical Research, UoS Milan, National Research Council, Rozzano, Milan, Italy; 3 Department of Biomedical Sciences, Humanitas University, Pieve Emanuele, Milan, Italy; 4 Discovery Biology, Bristol Myers Squibb Company, Redwood City, California, CA USA

**Keywords:** Tumour immunology, Cancer microenvironment, Immunosuppression

## Abstract

CD4^+^CD25^+^FOXP3^+^ regulatory T cells (Treg) are highly activated in tumors and promote disease progression. Specific, universal targeting of these effector Treg cells is limited by the lack of a conserved signature across human cancers and information on their origin. Here we combine analysis of single-cell RNA-sequencing datasets with spectral flow cytometry and identify a core signature of 88 genes consistently upregulated in intratumoral Treg cells among 9 epithelial cancers. We describe 4 Treg cell subsets – CCR7^+^ quiescent, CCR8^+^ effector, CD161^+^ and intermediate, with distinct tissue distribution, function, differentiation trajectories and molecular drivers. By single-cell T cell receptor sequencing, we observe that protumoral, effector CCR8^+^ Treg cells exhibit little clonal relationship with other Treg cell subsets inside tumors, but are clonally related to Treg cells in tumor-draining lymph nodes, as well as conventional T cells in tumors. This resource provides insights for development and fine-tuning of CCR8^+^ Treg cell-targeting therapies in cancer.

## Abstract (web summary)

Studies of conserved molecular programs in regulatory T cells (Tregs) across human tumors are limited. Here, by integrating scRNAseq datasets from 9 tumor types, the authors identify a common signature of human Treg cells across solid tumors, further dissecting the molecular and functional heterogeneity of human intratumoral Tregs.

## Introduction

CD4^+^CD25^+^FOXP3^+^ regulatory T cells (Treg) are central to immune homeostasis, tolerance and repair processes in human tissues^1^. Treg cells adapt to local niches to perform specialized functions in tissue maintenance, which is also reflected in their phenotypic, functional and molecular diversity^2^. On the contrary, Treg cells play a detrimental role in tumors, where they are preferentially recruited to interfere with antitumor immunity, thus promoting tumor growth and therapy resistance. Accordingly, a variety of preclinical studies in mouse models have shown that depletion or functional inhibition of Treg cells reinvigorates antitumor immunity, leading to tumor regression^3–5^, thus making Treg cells an attractive target of cancer immunotherapy. However, autoimmunity is a common side effect^6^, thereby requiring the identification of tumor-specific targets that might spare tissue-resident Treg cells^1^.

Studies in the last decade provided new knowledge on the phenotypic, functional and molecular characteristics of tumor-infiltrating Treg cells compared to those present in adjacent tissues, identifying receptors that are potential targets of depleting monoclonal antibodies. These include, among others, the chemokine receptor CCR8^7^, glycoprotein CD177^8^, neuropilin-1 (NRP1)^9^, TNF receptor superfamily members CD30, TNFR2, and 4-1BB, and interleukin receptors IL21R, IL1R2^10^, and IL1R1^11^. Some of these targets are currently being evaluated in phase I clinical trials across multiple metastatic cancers^12,13^. However, studies defining the conserved presence of these molecules across solid tumors are lacking, precluding the identification of “universal” targets of Treg cell-depleting immunotherapies.

Recent technological advancements, especially at the level of single cells, revealed that tumor-infiltrating T cells are largely heterogeneous in tumors, comprising subsets in a continuum of differentiation and functional maturation, and with possibly differential roles in progression of the disease and/or response to immunotherapy^14^. As far as Treg cells are concerned, a subset of CCR8^+^ICOS^+^4-1BB^+^IL1R1^+^ effector Treg cells (in some instances referred to as hyperactivated Treg cells) exerts superior inhibition of CD4^+^ conventional T cell (Tconv) proliferation in vitro when compared to “quiescent” Treg cells not expressing these antigens^11,15–17^. Effector Treg cell activation and function are controlled by specific transcriptional hubs involving interferon regulatory factor 4 (IRF4)^15^, its partner basic leucine zipper transcription factor ATF-like (BATF)^18^, mesenchyme homeobox 1 (MEOX1)^19^ and c-Rel transcription factors^20^. Moreover, their abundance predicts worse prognosis in multiple human cancers^15^, suggesting that preferential targeting of effector Treg cells may result in potentiation of antitumor immunity in the absence of autoimmunity.

Immune checkpoint blockade reshapes the tumor microenvironment, but may result in effector Treg cell accumulation in patients not responding to therapy^21–23^. Similarly, Treg cell depletion results in the accumulation of immunosuppressive Tconv cells acquiring cell-like features^24^. Thus, defining lineage relationships between intratumoral and tissue Treg cells and other populations of Tconv cells is fundamental to better understand possible mechanisms of secondary resistance. Poor T cell receptor (TCR) clonal overlap has been found between Treg cells from melanoma, ovarian or breast tumors and the adjacent tissue^25,26^. Instead, clonal relationships between a small population of CCR8^+^ Treg cells in the blood or metastatic lymph nodes (LN) of patients with breast cancer (BC) and CCR8^+^ Treg cells in tumors would suggest recruitment from other sites rather than differentiation in situ^27,28^. However, these studies, generally involving TCR sequencing of bulk Treg cell populations, did not clarify the lineage relationships between Treg cell subsets with precision.

In this study, we integrate single-cell RNA-sequencing (scRNA-seq) datasets from nine solid tumors and their related adjacent tissue, blood and, when possible, tumor-draining lymph nodes to characterize Treg cell heterogeneity, molecular characteristics, transcription factor usage, and clonal relationships. We identify a common signature of Treg cells across the studied tumors involving 88 genes and extend our knowledge of Treg cell heterogeneity in human cancer by classifying them in four subsets of C-C chemokine receptor 7 (CCR7)^+^ quiescent, CCR8^+^ effector, CD161^+^ and intermediate Treg cells, with different distribution across the body. Data are validated by high-dimensional spectral flow cytometry in breast and lung tumors. Finally, we study the clonality of effector Treg cells in non-small cell lung cancer (NSCLC) by single-cell TCR sequencing, to define the relationship with other intratumoral Treg cell subsets, with Treg cells in the normal adjacent tissue and tumor-draining lymph nodes, and with non-regulatory CD4^+^ Tconv. By high-resolution analyses, we provide insights into the heterogeneity and putative origin of effector Treg cells in human cancer that will be useful to design novel effective immunotherapies.

## Results

### Common signature of human Treg cells across solid tumors

**Fig. 1:**
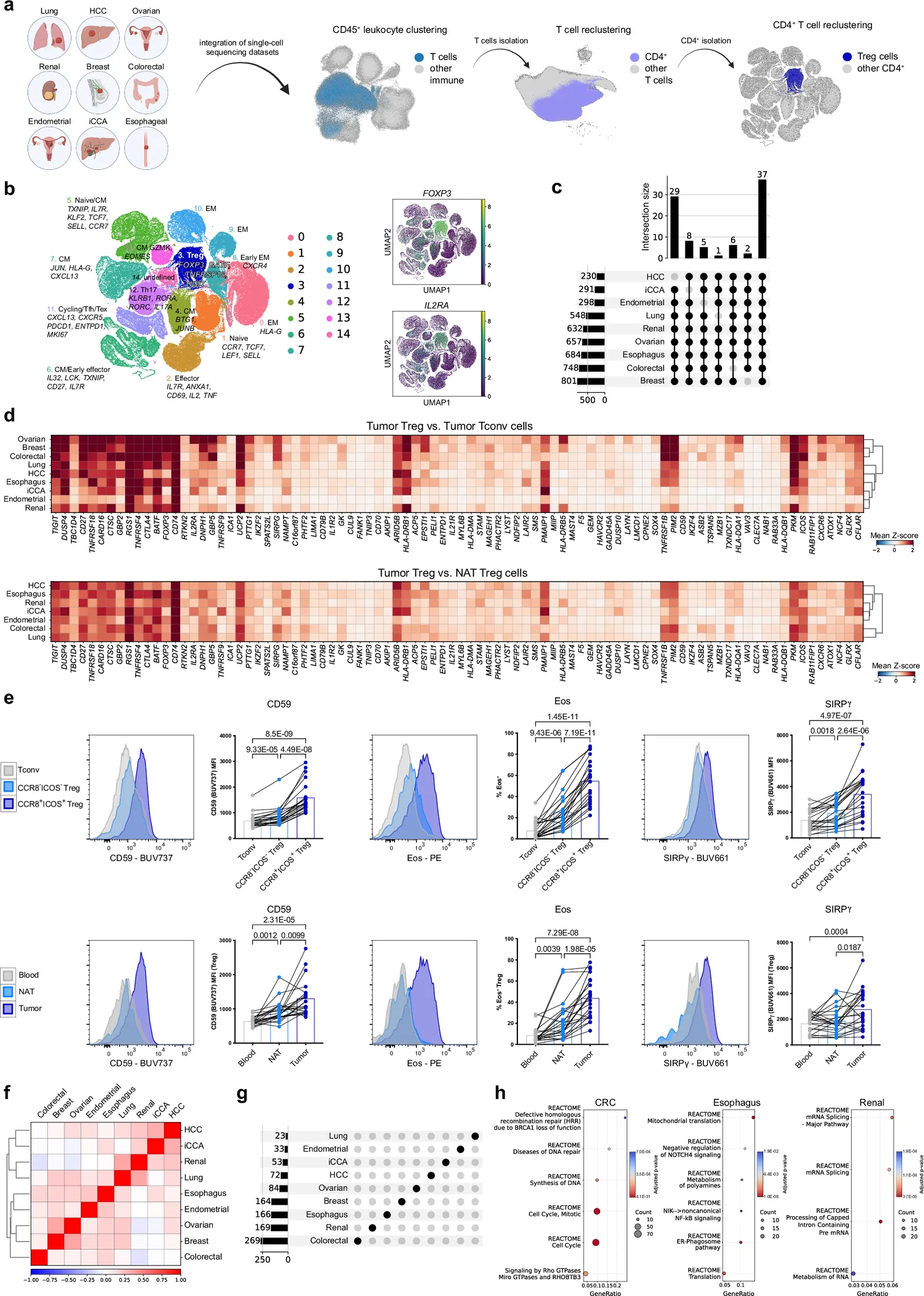
Single-cell atlas of regulatory T cells across nine solid tumors. **a** Schematic workflow of dataset integration and processing to create an atlas of 88,561 CD4^+^ T cells in nine solid tumors (left panel created in BioRender. lugli, E. (2026) https://BioRender.com/cj7af8y; UMAP plots created in this study, as described in Methods). **b** Left: UMAP plot representation of 15 CD4^+^ T cell clusters from integrated single-cell datasets of nine solid tumors (88,561 cells, *n* = 69 patients including *n* = 7 esophagus, *n* = 24 colorectal, *n* = 9 lung, *n* = 3 endometrial, *n* = 6 renal, *n* = 3 breast, *n* = 6 ovarian, *n* = 5 HCC, *n* = 6 iCCA, refers to (**b**–**d**, **f**–**h**). Labels indicate identity of each cluster (marker genes in Supplementary Fig. 1a); signature differentially expressed genes (DEGs) are indicated (FDR <0.05, LFC >1, two-sided Wilcoxon rank-sum test, Bonferroni correction). CM central memory, EM effector memory, Tfh T follicular helper, Tex T exhausted, Th17 T helper 17. Right: UMAP plots demonstrating *FOXP3* and *IL2RA* expression in CD4^+^ T cell clusters, identifying Treg cells. **c** Upset plot indicating numbers of DEGs (FDR <0.05; expressed in ≥20% of cells, two-sided *t*-test_overstim_var, Bonferroni correction) in tumor Treg vs. conventional CD4^+^ T cells (Tconv, rows) that are shared in at least eight of the tumors analyzed (columns). HCC hepatocellular carcinoma, iCCA intrahepatic cholangiocarcinoma. **d** Heatmap showing relative gene expression (mean Z-score) of tumor Treg versus Tconv cells (top) and of tumor Treg vs. NAT Treg cells of the 88 genes identified in (**c**). **e** Representative flow cytometry analysis of CD59, Eos, and SIRPγ on intratumoral Tconv, CCR8^-^ICOS^-^ and CCR8^+^ICOS^+^ Treg cells (top), or on Treg cells from blood, NAT and tumor (bottom) (gated as in Supplementary Fig. 4f). For each, summary of quantification, either as percentage of positive cells or median fluorescence intensity (MFI) of marker expression from 10 patients with BC and 12 with NSCLC is shown. One-way ANOVA with Tukey’s post-test. Numbers on graphs indicate *p* value. **f** The Pearson correlation matrix of Treg cell transcriptomic signatures. A dendrogram indicates similarity. **g** Upset plot indicating number of DEGs (FDR <0.05; expressed in ≥20% of cells, Two-sided *t*-test_overstim_var, Bonferroni correction), upregulated in tumor Treg versus Tconv cells, unique for tumor types. **h** Gene set enrichment analysis (selected REACTOME processes) of Treg cell upregulated genes unique for chosen tumor types, as identified in (**g**). One-sided hypergeometric test, g:SCS correction. For (**b**–**h**), source data are provided as a Source Data file.

To study total CD4⁺ and Treg cells across human cancers, we integrated scRNA-seq datasets from nine tumor types: non-small cell lung cancer (NSCLC), hepatocellular carcinoma (HCC), ovarian, renal, breast, colorectal (CRC), endometrial, esophageal, and intrahepatic cholangiocarcinoma (iCCA), encompassing a total of 69 patients. Samples were collected from four tissue types— primary tumor (77 samples), normal adjacent tissue (NAT, 47 samples), blood (13 samples), and lymph nodes (three samples). From a total of 671,163 CD45⁺ leukocytes, we isolated 200,697 CD3⁺ T cells and, from these, 88,561 single-positive CD4^+^ T cells, which underwent further subclustering (Fig. 1a). Using Harmony^29^ and deep embedded single-cell RNA-seq clustering (DESC)^30^ with a resolution of 0.6, we identified 15 distinct CD4⁺ T cell clusters with different abundance in tissues and cancer types (Fig. 1b, Supplementary Fig. 1a–c, and Supplementary Data 1). From CD4^+^ clusters, we extracted cluster C3 (12,418 cells), expressing higher levels of the Treg cell signature genes *FOXP3*, *IL2RA* (encoding CD25), *BATF*, *TIGIT*, *CTLA4* and members of the TNF receptor superfamily *TNFRSF18* (encoding GITR), *TNFRSF4* (OX40), and *TNFRSF1B* (TNFR2) compared to other clusters (Fig. 1b and Supplementary Fig. 1b), thereby implicating that C3 specifically identifies Treg cells, as supported by additional analyses below.

For each tumor type, we first computed differentially expressed genes between the Treg cell cluster and remaining CD4^+^ T cells (collectively referred to as Tconv; Supplementary Data 2). By further overlapping these lists, we identified genes upregulated in tumor Treg cells across all investigated tumors (Supplementary Data 3). In particular, 37 genes were consistently upregulated in Treg versus Tconv cells in all nine tumors, and 51 genes were upregulated in Treg cells in at least eight out of nine tumors (Fig. 1c). The identified 88 genes contained molecules well recognized as upregulated in activated, tumor-infiltrating Treg cells, such as *TIGIT*, *TNFRSF18*, *TNFRSF4*, *CTLA4*, *BATF*, *FOXP3*, *CD74*, *IL2RA*, *TNFRSF9* (encoding 4-1BB), *IL1R2*, *ENTPD1* (encoding CD39), *IL21R*, *MAGEH1*, *LAYN*, *TNFRSF1B*, *ICOS*, and *CXCR6*, among others. We also identified high expression of genes encoding two members of the Ikaros family of transcription factors, *IKZF2* (Helios) and *IKZF4* (Eos), the HLA-related genes *HLA-DRB1*, *HLA-DRB5*, *HLA-DQA1*, and the costimulatory receptor genes *CD27* and *CD70*. Moreover, we identified genes that have not been previously recognized as intratumoral Treg cell markers, such as *CD59* (inhibiting function of the complement system), *SIRPG* (signaling of phagocytic pathways), *GBP2*, *RGS1*, *GEM* (GTPase activity), *GLRX*, *TXNDC17* (disulfide oxidoreductases), *UCP2*, *PKM* (metabolism), and *ATOX1* (copper homeostasis), among others (Fig. 1d). *CCR8* was significantly upregulated in seven out of nine tumor types and exhibited increasing trend in the remaining 2 (HCC and iCCA) (Supplementary Fig. 1d), coherently with previous findings on its strong specificity for intratumoral Treg cells. Consistent with the identification of Treg cells, C3 exhibited the highest enrichment of the 88 gene signature among CD4^+^ T cell clusters (Supplementary Fig. 2a). Except for C11 of cycling follicular helper/exhausted T cells (Tfh/Tex), equivalent data were obtained when comparing C3 with Tconv clusters individually (Supplementary Data 4). The 88 Treg cell-specific genes were also generally upregulated in Treg cells residing in primary tumors compared to those from NATs from seven tumor types (for which NAT data were available from public datasets; Fig. 1d and Supplementary Data 5). To further explore the specificity, we integrated Treg cell samples from cancer-free donors^31,32^ onto our dataset, observing enrichment of the 88 gene signature in tumor Treg cells compared to Treg cells from blood, lymphoid (bone marrow, spleen, thymus, and lymph nodes) and non-lymphoid tissues (skin, intestine, lung, and fat) (Supplementary Fig. 2b), and to Treg cells from patients developing colitis following immune checkpoint blockade^33^ (Supplementary Fig. 2c). Additionally, the 88 gene signature was preferentially enriched in Treg cells from NSCLC tumors progressing to the same therapy (Supplementary Fig. 2d)^34^. We next validated the expression of a selected subset of markers at the protein level by high-dimensional spectral flow cytometry. In 22 samples of BC and NSCLC, CD59, Eos and SIRPγ were upregulated in Treg cells when compared to FOXP3^–^ Tconv cells, and their expression was highest in the CCR8^+^ICOS^+^ subset of effector regulatory T cells (Fig. 1e). These markers were also consistently upregulated by intratumoral vs. NAT Treg cells (Fig. 1e). In line with previous reports^10,35^, CCR8^+^ICOS^+^ intratumoral Treg cells also expressed increased levels of TNFR2, CXCR6 and IL21R when compared to their CCR8^–^ICOS^–^ counterparts and CD4^+^ Tconv cells, as well as when compared to Treg cells in NAT and blood (Supplementary Fig. 3a, b). CD70, a ligand generally expressed by activated T cells, and CD74, playing a role in motility and organelle/cytoskeleton organization of intratumoral Treg cells^36^, were also upregulated by CCR8^+^ICOS^+^ vs. CCR8^–^ICOS^–^ Treg cells but not by intratumoral vs. NAT Treg cells. On average, only ∼5% of the CCR8^+^ICOS^+^ cells expressed CD74 on the cell surface (Supplementary Fig. 3a, b), suggesting less rationale/limited availability to directly target this receptor in cancer^36^.

We next computed a Pearson correlation matrix based on total gene expression patterns (an approach unbiased by the number of Treg cells used and sampling depth) to compare Treg cells between different tumor types. As observed by hierarchical clustering, Treg cells from CRC differed from those isolated from other tumors, which, instead, were more similar to each other, such as in case of HCC and iCCA (possibly due to their shared localization in the liver niche) or breast and ovarian (possibly due to their predominance in women which would require further studies on Treg cell sex differences) (Fig. 1f). We also identified sets of differentially expressed genes between Treg and Tconv cells for each tumor. CRC Treg cells overexpressed the most unique set of genes when compared to all other tumors, followed by renal, esophagus and breast tumors (Fig. 1g and Supplementary Data 3). Gene set enrichment analysis revealed biological pathways characterizing Treg cells from specific tumors, such as mitosis and DNA synthesis in CRC or polyamine metabolism in esophageal cancer Treg cells (Fig. 1h and Supplementary Data 6). Pathways common to all cancers, emerging from the 88 gene signature, included Treg cell development, NF-κB signaling via TNF receptors, and immune activation (Supplementary Fig. 3c and Supplementary Data 6). Thus, these data identify common and unique features of Treg cells across human solid tumors.

### Intratumoral Treg cells consist of four subsets

**Fig. 2:**
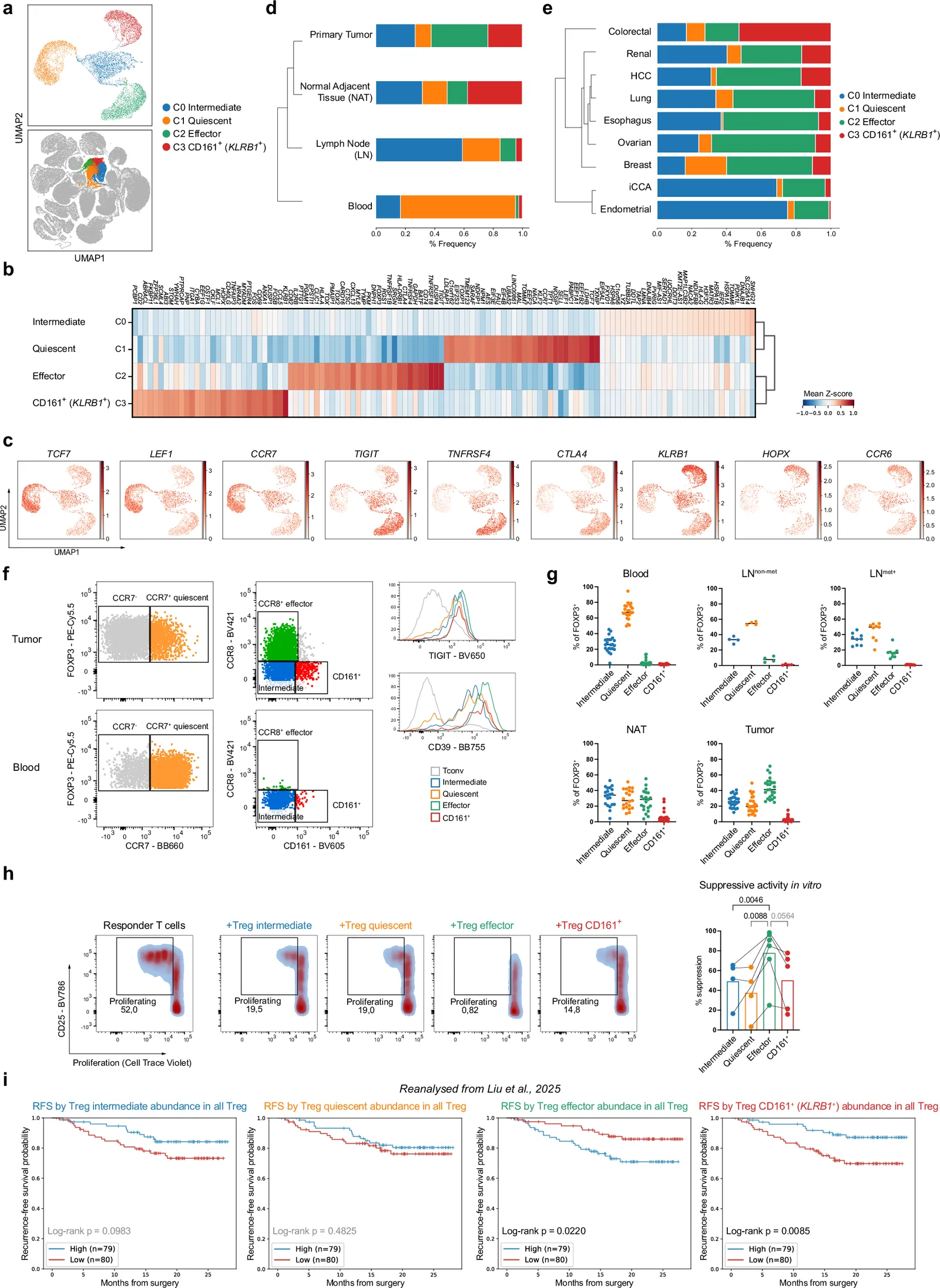
Regulatory T cells in tumors and tissues can be divided into four subsets. **a** Top: UMAP visualization of 12,418 regulatory T cells clustered into four subsets (DESC, 0.5 resolution). Bottom: 4 Treg cell subsets on CD4^+^ T cell UMAP visualization from Fig. 1b. Cluster identity is indicated on the right. **b** Heatmap showing relative gene expression of top 30 DEGs (FDR <0.05; expressed in ≥20% of cells) by four Treg cell clusters (**a**). **c** UMAP visualization of the expression of selected DEGs upregulated in quiescent (C1), effector (C2), and CD161^+^ (*KLRB1*^+^) (C3) Treg cell clusters. **d** Frequency (%) of Treg cell clusters in different tissues analyzed as relative to total Treg cells from each tissue. **e** Frequency (%) of Treg cell clusters in tumor tissue of different cancer types, analyzed as relative to total Treg cells from each tumor. For (**a**–**e**): *n* = 69 patients, including *n* = 7 esophagus, *n* = 24 colorectal, *n* = 9 lung, *n* = 3 endometrial, *n* = 6 renal, *n* = 3 breast, *n* = 6 ovarian, *n* = 5 HCC, *n* = 6 iCCA. **f** Left: Representative flow cytometry gating strategy to identify four Treg cell subsets previously identified by scRNA-seq, on selected blood and tumor samples from a patient with BC. CCR7^+^: quiescent; among CCR7^–^, CCR8^+^CD161^–^: effector; CCR8^–^CD161^+^: CD161^+^; CCR8^–^ CD161^–^: intermediate. Right: Representative flow cytometry analysis of TIGIT, CD39 on four Treg cell subsets in a BC tumor. Treg cells were gated as in Supplementary Fig. 4f. **g** Mean frequency (%) of the data in (**f**) from different tissues collected from patients with BC/NSCLC (*n* = 11 BC blood, 9 BC NAT, 13 BC tumor, 4 BC non-metastatic lymph node, 9 BC metastatic lymph node, 12 NSCLC blood, 12 NSCLC NAT, 12 NSCLC tumor samples). **h** Left: Representative flow cytometry proliferation profiles of CD4^+^ responder cells (Tresp) in an in vitro suppression assay with intratumoral Treg cell subsets from NSCLC. Right: mean % of suppression for the data shown on the left (*n* = 4 intermediate, 4 quiescent, 6 effector, 5 CD161^+^ Treg cells, depending on subset abundance and availability of sorted cells; three independent experiments; lines indicate samples from the same donors). Two-tailed paired *t*-test. Numbers on graphs indicate *p* value. **i** Kaplan–Meier curves showing RFS (recurrence-free survival) stratified by proportion of Treg cell subsets among all Treg cells in tumors (*n* = 159 patients with NSCLC). Two-sided log-rank test. For (**b**, **d**, **e**, **g**–**i**), source data are provided as a Source Data file.

We next dissected the heterogeneity of human Treg cells by performing subclustering of Treg single-cell data. By testing different resolutions, we obtained four clusters of Treg cells (individually labeled as C; Fig. 2a and Supplementary Fig. 4a, b), all expressing high levels of the Treg cell signature genes *FOXP3* and *IL2RA* compared to Tconv (Figs. 2a and 1b). The four Treg cell clusters were characterized by distinct gene expression profiles (Fig. 2b and Supplementary Data 7). When individually compared to the sum of all other clusters, C1, C2, and C3 were each represented by >1000 upregulated DEGs, while C0 contained only 47 upregulated DEGs. C1 expressed higher levels of genes related to naïve and early differentiated memory cells, such as *TCF7*, *LEF1*, *SELL* (encoding CD62L), *CCR7*, and *FOXP1*, thus identifying a cluster of quiescent Treg cells (Fig. 2b, c). C2 overexpressed *TIGIT*, *CTLA4*, *TNFRSF18*, *BATF*, and *TNFRSF4* (Fig. 2b, c), as well as *CCR8*, *ICOS*, and *IRF4* (Supplementary Data 7), reflecting the hyperactivated signature of effector Treg cells. C3 expressed, among others, high levels of *KLRB1* (encoding CD161), *CD69*, *HOPX*. and *CCR6* (Fig. 2b, c), thereby resembling cells with T helper 17 (Th17)-like identity. Genes upregulated in C3 also contained cytokine genes generally expressed by Tconv subsets, such as *IL2*, *TNF*, *IFNG*, and *IL17A*, as well as the Tconv cell transcription factor genes *RORA*, *RORC*, and *TBX21* (encoding T-bet) (Supplementary Data 7). These data suggest that C3 may reflect Treg cells with plasticity of differentiation towards Th1 and Th17 lineages, in some reports referred to as “unstable” or “fragile” Treg cells in mice and humans, primarily in autoimmune diseases^37,38^. Despite being more similar to C1 than C2 and C3 cells by metaclustering, C0 lacked specific genes with known function, precluding a clear definition (hereafter referred to as intermediate). Among these clusters, C2 of effector Treg cells showed preferential enrichment of the intratumoral Treg cell 88 gene signature, as well as of a manually curated signature of functional suppressive molecules (Supplementary Fig. 4c, d).

The four subsets of intratumoral Treg cells exhibited differential distribution across tissues, with quiescent and intermediate clusters predominantly present in blood and LNs and the effector cluster in tumors. Normal adjacent tissue contained a significant number of all four Treg cell subsets, but also contained the largest proportion of CD161^+^ (*KLRB1*^+^) Treg cells (Fig. 2d). We next studied whether the four Treg cell clusters are differently distributed in tumor tissues across the nine cancer types studied. In line with their unique gene expression pattern observed in Fig. 1g, CRC Treg cells were significantly different, with relatively high abundance of the CD161^+^ (*KLRB1*^+^) cluster (Fig. 2e). Instead, other tumor types predominantly contained effector and intermediate Treg cell subsets (Fig. 2e and Supplementary Fig. 4e). By stringently gating Treg cells as CD4^+^CD25^hi^CD127^lo^FOXP3^+^ cells (Supplementary Fig. 4f), we further confirmed the existence of the four Treg cell subsets by flow cytometry and supervised manual gating in our BC and NSCLC cohorts. CCR7 expression enabled the identification of quiescent Treg cells, followed by detection of CCR8^+^ effector and CD161^+^ Treg cells among the CCR7^–^ subset. Finally, CCR7^–^CCR8^–^CD161^–^ cells constituted the intermediate subset (Fig. 2f). All four subsets in tumors expressed higher levels of Treg cell suppressive molecules (TIGIT, CD39), activation markers (OX40, GITR) and lineage markers (FOXP3 and CD25) when compared to Tconv cells, further confirming Treg cell identity (Fig. 2f and Supplementary Fig. 4g). Flow cytometry confirmed scRNA-seq distribution profiles of these subsets according to which quiescent and intermediate Treg cells were most abundant in blood and LNs, effector Treg cells in primary tumor and CD161^+^ Treg cells only in normal adjacent tissue (Fig. 2g). We next performed functional characterization of the four Treg cell subsets purified from NSCLC tumors. In line with their Treg cell identity, all 4 Treg cell subsets efficiently suppressed autologous responder T cell proliferation in vitro, among which CCR8^+^ effector Tregs exhibited the highest suppressive activity (Fig. 2h and Supplementary Fig. 5a, b). Effector Treg cells produced little to no T helper/proinflammatory cytokines, while CD161^+^ Treg cells produced IL-2 and exhibited high INF-γ^+^/TNF^+^ polyfunctionality (Supplementary Fig. 5c). Finally, we studied the prognostic role of the 4 Treg cell subsets by re-analyzing scRNA-seq data of 159 patients with NSCLC^17^ in terms of disease recurrence post-surgical tumor resection. High abundance of effector Treg cells predicted lower recurrence-free survival, while high abundance of CD161^+^ (*KLRB1*^+^) Treg cells correlated with higher recurrence-free survival (Fig. 2i). High abundance of effector Treg cells was also inversely correlated with the presence of all the other Treg cell subsets (Supplementary Fig. 5d).

Overall, we identify four subsets of human regulatory T cells, with differential gene expression, tissue distribution and functional activity, as well as different relevance to NSCLC clinical outcome in terms of post-surgery disease recurrence.

### Differentiation and molecular regulators of four subsets of Treg cells

**Fig. 3:**
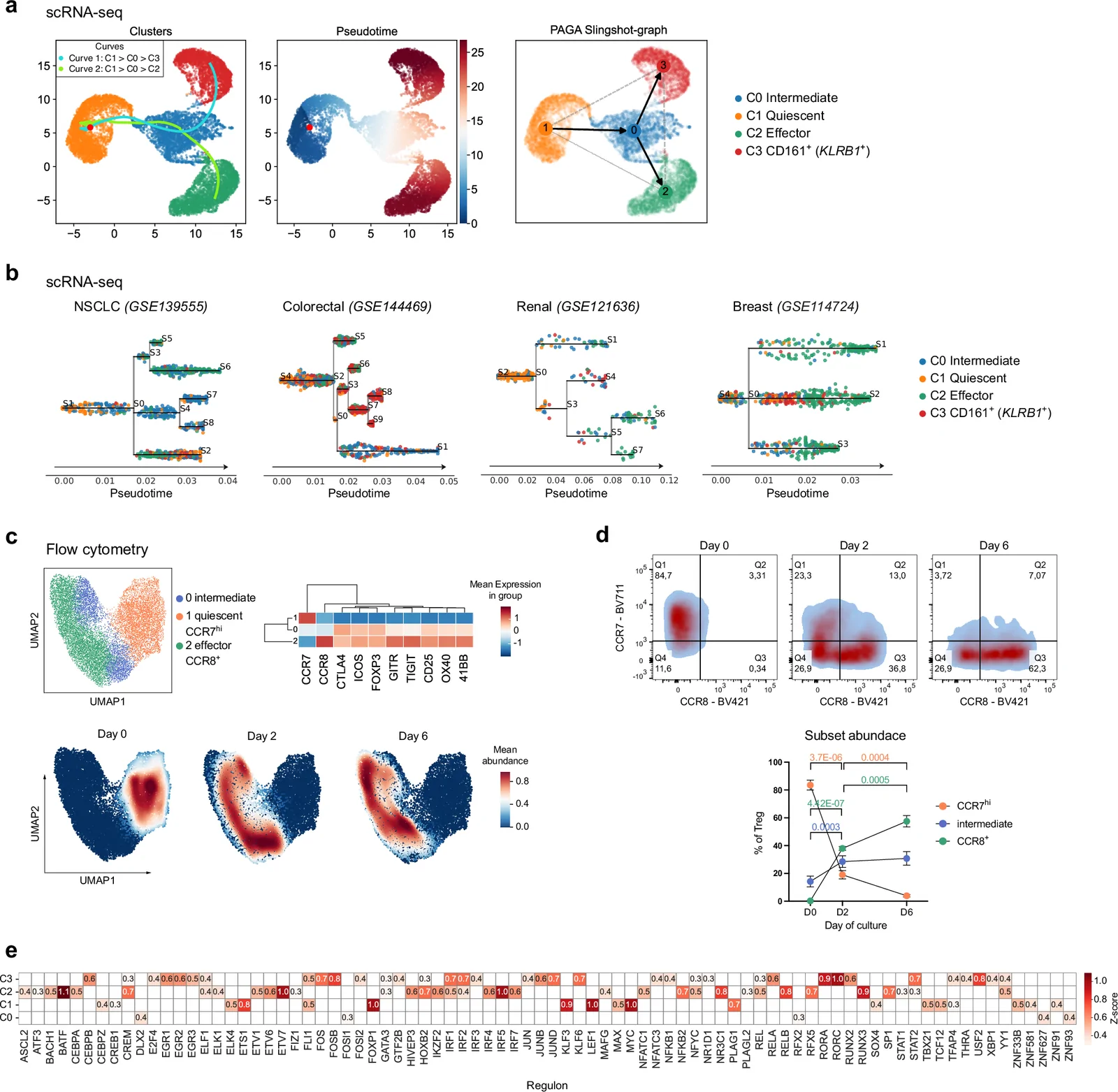
Differentiation and molecular regulators of four subsets of Treg cells. **a** Slingshot differentiation trajectories of Treg cells clustered into four subsets, as in Fig. 2a. Left: two inferred trajectories are marked by light blue and light green lines. Red dot indicates the starting point of inferred trajectories (C1). Middle: pseudotime analysis of four Treg cell subsets. Right: PAGA (Partition-based graph abstraction) Slingshot graphs estimating connectivity of Treg clusters and predicted direction of differentiation. Dashed lines indicate potential differentiation, but at a lower probability. **b** STREAM differentiation trajectories of Treg cells in lung, colorectal, renal, and breast cancer scRNA-seq datasets. GEO repository numbers are indicated in brackets. Identity of Treg cell clusters is indicated. Analysis performed on cells from patients with *n* = 6 NSCLC, *n* = 8 colorectal, *n* = 3 renal, *n* = 3 breast. **c** Top left: UMAP visualization of 15,000 CD4^+^CD25^+^FOXP3^+^ Treg cells clustered into three subsets by the CRUSTY platform and PhenoGraph algorithm. Treg cells were concatenated from 15 samples (*n* = 5 healthy donors, at day 0, 2, 6 of ex vivo cell culture). Top right: Heatmap showing relative expression of markers (columns) by cluster (rows). Bottom: UMAP representation of Treg cell distribution, according to timepoint (day) of ex vivo culture. **d** Top: representative flow cytometry plots of CCR7 and CCR8 on CD4^+^CD25^+^FOXP3^+^ Treg cells, sorted as CCR7^hi^ quiescent and cultured ex vivo, according to timepoint (day) of culture. Bottom: mean ± SD % of indicated subsets among Treg cells, for data shown on top (*n* = 5 healthy donors). For (**c**, **d**), Treg cells were gated as in Supplementary Fig. 6b. Statistics indicate differences between days of culture for each subset, depicted in different  colors (*n* = 5 healthy donors, 2 independent experiments). Two-tailed paired *t*-test. Numbers on graphs indicate *p* value. **e** SCENIC analysis of predicted transcription factor regulon activity in Treg cell subsets. Color scale and values indicate the Z-score of activity. White color: lack of activity. For panels (**a**, **e**), the analysis was performed on cells from *n* = 69 patients, including *n* = 7 esophagus, *n* = 24 colorectal, *n* = 9 lung, *n* = 3 endometrial, *n* = 6 renal, *n* = 3 breast, *n* = 6 ovarian, *n* = 5 HCC, *n* = 6 iCCA. For (**d**, **e**), source data are provided as a Source Data file.

To gain insight into relationships of the four Treg cell subsets, we performed Slingshot^39^ trajectory analysis, setting quiescent cells as the starting point. Slingshot predicted differentiation of quiescent Treg cells into intermediate and eventually into either CD161^+^ (*KLRB1*^+^) or effector Treg cell lineages (Fig. 3a). We next performed analysis of transcriptional trajectories using the STREAM algorithm^40^ to further elucidate the relationship between Treg cell subsets in different tumor types. The identity of the four Treg cell subsets was superimposed on subway map/STREAM plots, indicating differentiation/cell fate in pseudotime. Quiescent cells were used as a starting point for the inferred trajectories. Treg cells generally transitioned through the intermediate state and further separated into different branches featuring intermediate, CD161^+^ (*KLRB1*^+^) or effector cells, supporting the concept that intermediate Treg cells may not be fully committed in human tumors (Fig. 3b). Such relationship was further corroborated by ex vivo differentiation of sorted CCR7^+^ quiescent Treg cells from healthy donors. Anti-CD3/28-mediated activation of quiescent Treg cells in the presence of A549 lung cancer cell conditioned medium led to a gradual loss of CCR7 expression and acquisition of intermediate phenotype following 2 days of culture, with the majority of cells eventually becoming CCR8^+^ effector and a fraction of Treg cells maintaining the intermediate phenotype after 6 days (Fig. 3c, d and Supplementary Fig. 6a, b).

To further delineate the molecular identity of Treg cell subsets, we performed analysis of gene regulatory networks on the Treg cell dataset from tumoral tissue by SCENIC^41^, an algorithm capable of predicting transcription factor activity by analysis of footprints on promoters of expressed genes. Quiescent cells (C1) were clearly defined, among others, by activity of FOXP1, KLF3 and LEF1, known for their role in the long-term maintenance of naïve and stem-like T cells (Fig. 3e). Effector Treg cells (C2) were characterized by increased predicted activities of BATF, IRF4 and RELB, consistent with previous reports^15,18^, and by the less-described ETV7, IRF5 and RUNX3 transcription factors. These cells also had lower predicted activity of MYC, reported to be highly repressed by FOXP3^42^, and whose expression was upregulated compared to other Treg clusters. Consistent with gene expression data, CD161^+^ (*KLRB1*^+^) Treg cells (C3) had increased predicted activities of RORA and RORC, promoting the differentiation to the Th17 lineage, as well as EGR, FOS and JUN family members. We could not identify defined transcriptional regulators of C0 intermediate Tregs, further suggesting the transitional identity of these cells (Fig. 3e).

Overall, we describe two predominant differentiation trajectories and identify predicted molecular drivers of subsets of human intratumoral Treg cells.

### Different effector Treg cell subsets exist in breast and lung tumors, healthy tissue and draining lymph nodes

**Fig. 4:**
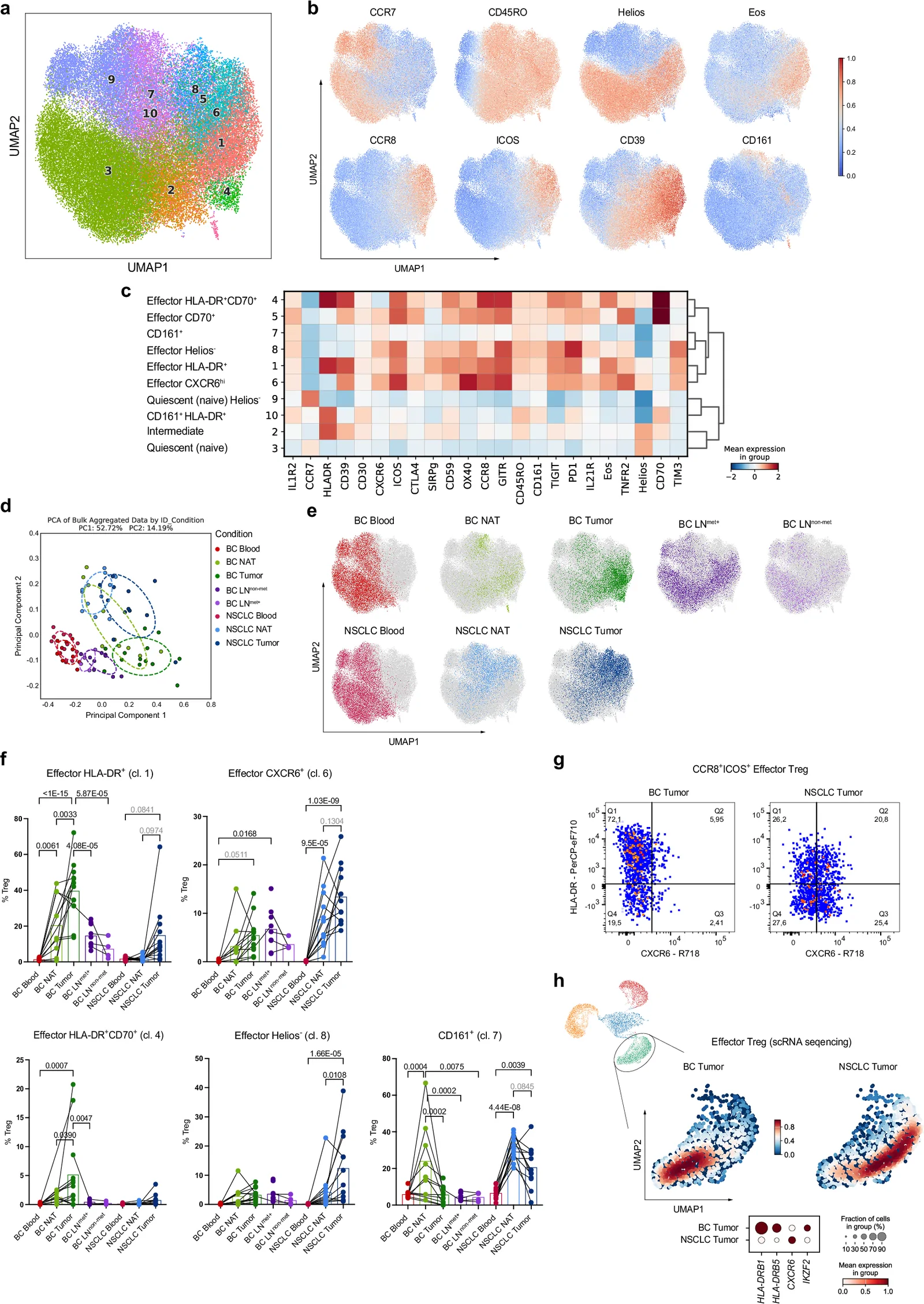
Identification of Treg cell subsets by flow cytometry in breast and lung tumors. **a** UMAP visualization of 55,406 CD3^+^CD4^+^CD25^hi^CD127^lo^FOXP3^+^ Treg cells clustered into 10 subsets by the CRUSTY platform and FlowSOM algorithm. Treg cells were gated as in Supplementary Fig. 4f and concatenated from 82 samples (*n* = 11 BC blood, 9 BC NAT, 13 BC tumor, 4 BC non-metastatic lymph node, 9 BC metastatic lymph node, 12 NSCLC blood, 12 NSCLC NAT, 12 NSCLC tumor samples, for all data in (**a**–**e**)). **b** UMAP visualization of relative expression of selected markers from data in (**a**). **c** Heatmap showing relative expression of markers (columns) by cluster (rows) identified in (**a**). An arbitrary nomenclature of clusters based on the expression of protein markers is reported. **d** Principal component analysis (PCA) of all Treg cell samples based on mean expression of proteins analyzed by flow cytometry. Colors identify samples from each condition (tumor type and tissue). Ellipses indicate similarity of different groups. **e** UMAP representation of Treg cell distribution from (**a**), according to tumor type and tissue. **f** Mean frequency of selected Treg cell clusters, as identified in (**c**), according to tumor type and tissue. Lines indicate samples from the same patients (*n* = 11 BC blood, 9 BC NAT, 13 BC tumor, 4 BC non-metastatic lymph node, 9 BC metastatic lymph node, 12 NSCLC blood, 12 NSCLC NAT, 12 NSCLC tumor samples). One-way ANOVA with Tukey’s post-test. Numbers on graphs indicate *p* value. **g** Representative flow cytometry plots of HLA-DR and CXCR6 on effector Treg cells (identified as CCR8^+^ICOS^+^) in representative BC and NSCLC tumor samples. Orange and red dots correspond to gradually higher cell density. **h** Top: UMAP visualization of intratumoral effector Treg cells (C2, from Fig. 2a), according to tumor type. Bottom: Dot plot showing expression level and frequency of expressing cells for selected genes, in intratumoral effector Treg cells, according to tumor type (*n* = 9 patients with NSCLC, *n* = 3 patients with BC). For (**c**, **d**, **f**, **h**), source data are provided as a Source Data file.

To further study the distribution of Treg cells across tissues and different tumors, as well as dissect heterogeneity of the effector Treg cell subset, we designed a 34-parameter spectral flow cytometry panel including signature markers informed by scRNA-seq (Supplementary Data 8). We profiled T cells from a validation cohort of patients with BC and NSCLC for a total of 23 blood, 21 NAT, 4 non-metastatic, 9 metastatic lymph nodes, and 25 tumor samples. Treg cells were isolated as viable CD3^+^CD4^+^CD25^hi^CD127^lo^FOXP3^+^ (Supplementary Fig. 4f). Consistent with previous reports, both total Treg and CCR8^+^ICOS^+^ effector Treg cells were highly abundant in tumor samples (Supplementary Fig. 6c, d). Data mining using the CRUSTY platform^43^ and FlowSOM clustering identified 10 different Treg cell clusters (Fig. 4a), characterized by varying expression of protein markers (Fig. 4b, c), including multiple markers identified in the conserved transcriptomic tumor Treg cell signature reported in Fig. 1d. The identified Treg cell clusters included subsets with quiescent, intermediate, CD161^+^ and effector identity, the latter comprising five clusters overexpressing CCR8, ICOS, OX40, GITR, TIGIT, PD-1, and CD59 at different intensities. The effector clusters differed predominantly by the expression of CD70, HLA-DR, CXCR6, TIM-3 and Helios (Fig. 4c).

Principal component analysis based on expression of markers analyzed by flow cytometry distinguished tumors from their respective bloods and NATs, although the BC NAT cohort tended to be more heterogeneous than the NSCLC NAT cohort (Fig. 4d). Interestingly, Treg cells infiltrating both NAT and primary tumors exhibited significant differences between patients with BC and NSCLC (Fig. 4d, e), indicating tumor-specific adaptation, as previously suggested by scRNA-seq.

Quantification of specific effector subsets identified by CRUSTY revealed higher prevalence of effector Treg cell clusters expressing HLA-DR (cluster 1 and cluster 4, the latter expressing also CD70) in BC tumor samples, and higher prevalence of HLA-DR^–^CXCR6^hi^ effector Treg cell cluster (cluster 6) in NSCLC, but also in BC metastatic LNs compared to BC blood (Fig. 4f, g and Supplementary Fig. 6e). Although we could not observe major differences between Treg cell populations infiltrating metastatic and non-metastatic LNs, possibly due to the low number of available samples, effector Treg cell clusters were generally more abundant in metastatic lymph nodes, when compared to non-metastatic ones, which instead contained more quiescent-phenotype cells. Finally, an effector Treg cell cluster lacking Helios (cluster 8) was only detected in NSCLC tumor samples (Fig. 4f). Differences in tumor effector Treg cells in BC and NSCLC were similarly observed in scRNA-seq data, with prevalence of expression of *HLA-DRB1*, *HLA-DRB5*, and *IKZF2* (encoding Helios) in BC and *CXCR6* in NSCLC, consistent with flow cytometry data (Fig. 4h and Supplementary Data 9).

These data confirm the occurrence of a common Treg cell signature across cancer types featuring the presence of effector-related markers, but also the acquisition of diverse phenotypes, underlying tumor-specific adaptations.

### Intratumoral *CCR8*-expressing effector Treg cells are clonally expanded and related to neighboring tissues

**Fig. 5:**
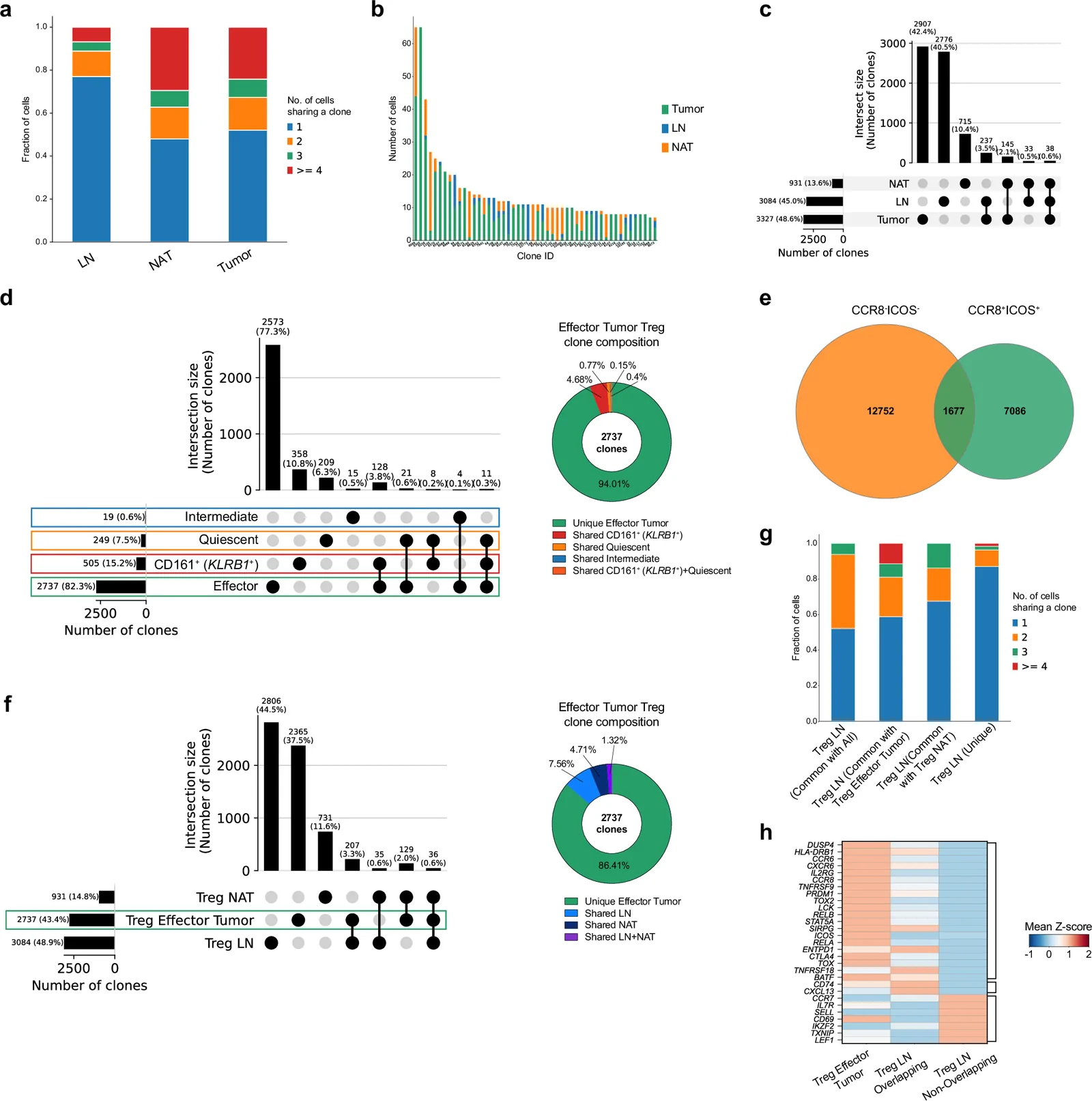
Clonal relationship of intratumoral effector Treg cells and other Treg cell subsets in NSCLC. **a** Number of Treg cells (1, 2, 3, or ≥4) bearing the same TCR clone (expressed in frequency, %) in LN, NAT and tumor. A higher number of cells sharing the same clone indicates clonal expansion. **b** Tissue distribution of the 50 most abundant clones of Treg cells in the analyzed dataset. **c** Shared TCR clonotypes between Treg cells from tumor, NAT and tumor-draining LN. Vertical bar size indicates the amount and fraction (%) of unique or shared clonotypes. Horizontal bar size indicates the total number of clones in a tissue. **d** Left: shared TCR clonotypes between the four subsets of Treg cells (Fig. 2a) inside the NSCLC tumor tissue. Data were expressed as in (**c**). Right: donut graph indicating composition of effector tumor Treg cell clones. Percentages of unique or clones shared with other Treg cell subsets are indicated. **e** Venn diagram showing overlap of detected TCR sequences reconstructed from bulk RNA sequencing of CCR8^+^ICOS^+^ and CCR8^–^ICOS^–^ Treg cells sorted from *n* = 5 patients with NCSLC. **f** Left: shared TCR clonotypes between the effector Treg cells inside the NSCLC tumor tissue and total Treg cells from NAT and tumor-draining LN. Data are expressed as in (**c**). Right: donut graph indicating composition of effector tumor Treg clones. Percentages of unique or NAT/LN-Treg-shared clones are indicated. **g** Number of Treg cells (1, 2, 3, or ≥4) bearing the same TCR clone (expressed in frequency, %) in tumor-draining LN, depending on their overlap with effector tumor Treg and/or NAT Treg cells. A higher number of cells sharing the same clone indicates clonal expansion. **h** Heatmap showing relative gene expression of manually curated differentially expressed genes (FDR <0.05; expressed in ≥20% of cells, Two-sided Wilcoxon rank-sum test, Bonferroni correction) of effector tumor and clonally overlapping/nonoverlapping tumor-draining LN Treg cells, as identified in Supplementary Data 11. For (**a**–**d**, **f**–**h**), cells were analyzed from tumor, NAT and tumor-draining LN from *n* = 3 patients with NCSLC. For (**a**–**h**), source data are provided as a Source Data file.

As ongoing clinical trials are targeting CCR8-expressing Treg cells, it is of crucial importance to dissect the origin of these cells to predict Treg cell dynamics following depletion. To address this aspect with enhanced precision, we studied the origins of the CCR8-expressing effector Treg cell subset in human cancer by coupled scRNA and T cell receptor (TCR) sequencing, taking advantage of a public dataset of T cells from matched NSCLC tumor, tumor-draining LN and NAT from ref. ^34^. A total of 39,542 CD4^+^ T cells (including 9451 Treg cells and 30,091 Tconv cells) from matched tissues of three patients with NSCLC could be retrieved. Initial analysis of the total Treg cell pool revealed that cells in primary tumors and NATs undergo significant clonal expansion, while those in LNs are characterized by unique TCR sequences (Fig. 5a). Among the top 50 expanded Treg clonotypes, most resided preferentially in the tumoral tissue, suggesting robust activation and expansion at this site (Fig. 5b). In some cases, the same Treg cell clone was found to be shared among the three tissues, thus indicating recirculation potential (Fig. 5c). Transcriptomic analysis of these shared TCR clonotypes revealed a different gene expression pattern depending on tissue localization (Supplementary Fig. 7a and Supplementary Data 10), according to which Treg cell clones residing in LN overexpressed the stem-like gene *CCR7*^44^ and the antioxidant genes *GPX4* and *PRDX2*, those in NAT the tissue residency genes *RUNX3* and *CD69* and those in tumors the effector Treg cell-related genes *CCR8*, *CXCR6*, *TNFRSF9*, and *MAGEH1*. These data indicate dynamic circulation of cells between tissues and maturation into effector cells inside the tumor bed.

We next focused on the origin of the *CCR8*-expressing effector Treg cell subset specifically. We isolated Treg cells from NSCLC tumor tissue specimens and overimposed transcriptomic identities of the four Treg cell clusters previously identified in our tumor Treg cell atlas from Fig. 2a, thus enabling to assign the same identity coupled with TCR information. We then sought to investigate three potential sources of effector tumor Treg cells, i.e., development from other intratumoral Treg cell subsets, migration from LN/NAT, or differentiation from CD4^+^ Tconv cells. Effector tumor Treg cells exhibited little clonal relationship with other subsets inside the tumor bed, as only 164 clones out of 2737 (ca. 6%) were shared with other Treg cell subsets, mostly the CD161^+^ (*KLRB1*^+^) subset (Fig. 5d and Supplementary Fig. 7b). We confirmed these results by analyzing a second scRNA/TCR-seq dataset of 231 patients with NSCLC^17^, revealing less than 2% of effector Treg cell clonal overlap with the other subsets inside tumors (Supplementary Fig. 7c). Finally, TCR sequence reconstruction from a bulk RNA-seq dataset using the TRUST4 algorithm^45^ revealed poor clonal overlap between sorted CCR8^+^ICOS^+^ and CCR8^-^ ICOS^-^ Treg cells from NSCLC (Fig. 5e and Supplementary Fig. 7d, e)^15^. These populations, respectively, were transcriptionally equivalent to C2 effector, and C1 quiescent and C3 CD161^+^ (*KLRB1*^+^) clusters of Treg cells identified in this study (Supplementary Fig. 7d). All together, these data confirm results obtained with scTCR-seq.

We then speculated that effector tumor Treg cells may rather originate from tumor-adjacent tissues and acquire effector identity in the tumor bed. In line with this hypothesis, 372 out of 2737 (ca. 14%) effector tumor Treg cell clones were shared with Treg cells in the LN or NAT, the majority of which (207 of 372; ca. 56%) were shared with the LN Treg cells only (Fig. 5f and Supplementary Fig. 8a). Both LN and NAT Treg cells that shared TCR clones with intratumoral effector Treg cells exhibited clonal expansion compared to nonoverlapping clones (Fig. 5g and Supplementary Fig. 8b), suggesting activation and expansion. To further validate this aspect, we computed differentially expressed genes between tumor effector Treg cells and clonally related and unrelated LN Treg cells. Effector tumor Treg cells upregulated 1148 genes, while clonally related and unrelated LN Treg cells upregulated only 51 and 91 genes, respectively (Supplementary Data 11). Genes upregulated by Treg cells in tumors included effector genes such as *DUSP4*, *CXCR6*, *CCR8*, *TNSFRSF9*, *SIRPG*, *ICOS*, *ENTPD1*, *CTLA4*, and *BATF*, while Treg cell clones unique to the LN upregulated naïve/quiescence genes such as *CCR7*, *SELL*, *IL7R*, and *LEF1*. LN Treg cells clonally related to those in tumors lacked naïve genes, but exhibited intermediate expression of effector genes, suggesting gradual maturation already initiated in tumor-draining LNs (Fig. 5h and Supplementary Fig. 8d) that is reminiscent of the quiescent→intermediate→effector Treg cell differentiation model reported in Fig. 3a–d. A similar trend of effector gene upregulation could be observed in NAT Treg cells clonally related to tumor Treg cells compared to unrelated ones (Supplementary Data 12 and Supplementary Fig. 8c). Overall, these data suggest that intratumoral effector Treg cells might originate, at least in part, from adjacent tissues, especially tumor-draining LNs.

### A subset of CD4^+^ Tconv cells phenotypically resembles and is clonally related to intratumoral effector Treg cells

**Fig. 6:**
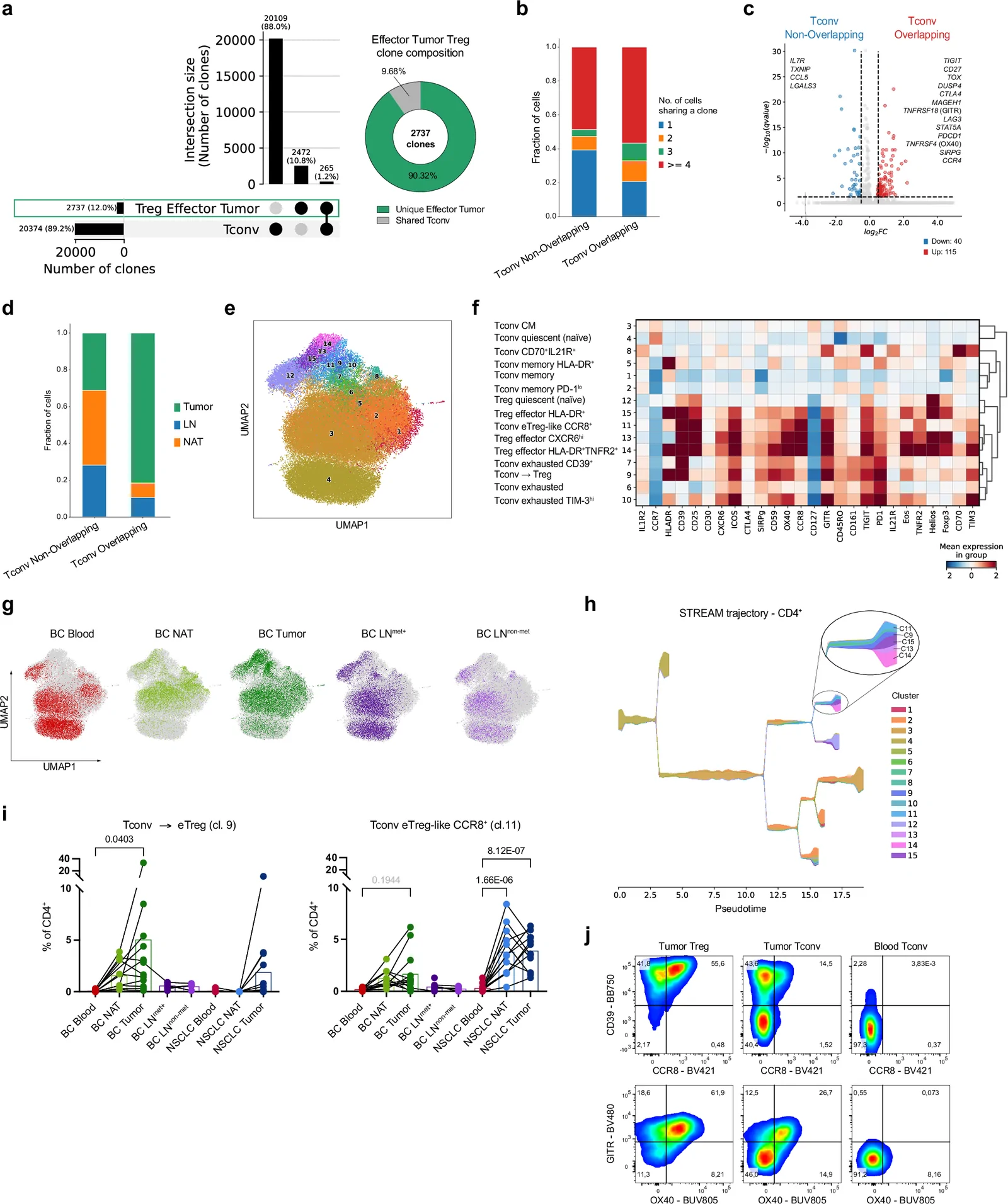
Conventional T cells can exhibit a clonal relationship with effector Treg cells. **a** Left: shared TCR clonotypes between intratumoral effector Treg cells and total conventional CD4^+^ T cells (Tconv) from tumor, NAT and tumor-draining LN from *n* = 3 patients with NCSLC. Vertical bar size indicates the amount and fraction (%) of unique or shared clonotypes. Horizontal bar size indicates the total number of clones in each condition. Right: donut graph indicating the composition of effector tumor Treg cell clones. **b** Number of CD4^+^ Tconv cells (1, 2, 3, or ≥4) bearing the same TCR clone (%) depending on their overlap with intratumoral effector Treg cells. Data refer to samples from *n* = 3 patients with NCSLC. **c** Volcano plot of differentially expressed genes between CD4^+^ Tconv cells from (**a**, **b**). Genes with LFC >0.5 and FDR <0.05 (two-sided Wald test, Benjamini–Hochberg correction) were considered significant. Selected DEGs of interest are indicated. **d** Tissue distribution of CD4^+^ Tconv cells from (**a**, **b**). **e** UMAP visualization of 76,774 total CD3^+^CD4^+^ T cells clustered into 15 subsets by the CRUSTY platform and FlowSOM algorithm. CD4^+^ T cells were gated as in Supplementary Fig. 4f and concatenated from 82 samples (*n* = 11 BC blood, 9 BC NAT, 13 BC tumor, 4 BC non-metastatic LN, 9 BC metastatic LN, 12 NSCLC blood, 12 NSCLC NAT, 12 NSCLC tumor samples). **f** Heatmap showing relative expression of markers (columns) by cluster (rows) identified in (**e**). An arbitrary nomenclature of clusters based on the expression of markers is reported. **g** CD4^+^ T cell distribution in different tissues from patients with BC from (**e**). **h** Pseudotime STREAM trajectory of CD3^+^CD4^+^ T cell clusters identified in (**e**, **f**). **i** Mean frequency of selected Tconv cell clusters identified in (**e**, **f**), according to tumor type and tissue. Lines indicate samples from the same patients (*n* = 11 BC blood, 9 BC NAT, 13 BC tumor, 4 BC non-metastatic LN, 9 BC metastatic LN, 12 NSCLC blood, 12 NSCLC NAT, 12 NSCLC tumor samples). One-way ANOVA with Tukey’s post-test. Numbers on graphs indicate *p* value. **j** Representative flow cytometry plots of CD39/CCR8, GITR/OX40, on different CD4^+^ T cell populations from a representative patient with BC (gated as in Supplementary Fig. 4f). For (**a**–**d**, **f**, **i**), source data are provided as a Source Data file.

Previous studies of TCR clonality of bulk intratumoral human Treg cells have identified a degree of clonal relationship with FOXP3^–^ Tconv cells in solid tumors^25,26^. However, whether this relationship occurs directly between Tconv and CCR8-expressing effector Treg cells has not been elucidated. Moreover, a recent study in mice revealed that, upon Treg cell depletion in tumor-bearing animals, a subset of CCR8^+^ Tconv cells may expand and acquire effector Treg cell-like features, while remaining Foxp3^–^. The same study reported that CCR8^+^FOXP3^–^ Tconv cells can be found in human NSCLC^24^. On the basis of these observations, we tested whether tumor effector Treg cells are clonally related to CD4^+^ Tconv cells. In the Caushi et al. dataset, 265 out of 2737 (ca. 10%) effector tumor Treg cell clones were also detected among CD4^+^ Tconv cells (Fig. 6a) and in 2/3 of the patients (Supplementary Fig. 9a). In the Liu et al. dataset of 231 NSCLC, the overlap could be detected in 2.6% of effector Treg cell clones and in ca. 76% of tumors, with varying degrees (Supplementary Fig. 9b, c). These overlapping Tconv cells were more clonally expanded (Fig. 6b) and upregulated the effector Treg cell-related genes *TIGIT*, *DUSP4*, *CTLA4*, *TNFRSF18*, *TNFRSF4*, and *SIRPG* compared to the nonoverlapping ones (Fig. 6c, Supplementary Fig. 9d, e, and Supplementary Data 13 and 14) and resided predominantly in the tumoral tissue (Fig. 6d).

Next, we validated whether we could observe a similar population of Tconv cells that is phenotypically related to effector Treg cells in our cohort of 33 patients with BC and NSCLC, analyzed by high-dimensional flow cytometry. Using CRUSTY analysis and FlowSOM clustering of CD4^+^ T cell data, we identified 15 clusters, including four clusters of CD25^hi^FOXP3^+^ Treg cells (C12, C13, C14, and C15) and four clusters of FOXP3^–/lo^Helios^–^PD-1^hi^TIGIT^hi^ CD4^+^ Tconv cells (C6, C7, C9, and C10), resembling exhausted cells (Fig. 6e–g). C11 was FOXP3^lo^ but phenotypically resembled effector Treg cells, expressing high levels of CD25, CCR8, CD39, GITR, and OX40 (Fig. 6f). We speculated that some clusters correspond to Tconv that would eventually convert into Treg/Treg-like cells, or vice versa. To gain more insights into this relationship, we applied the STREAM algorithm to flow cytometry data. In line with our hypothesis, clusters 9 and 11 of Tconv cells established one trajectory node together with effector Treg cell clusters 13, 14, and 15 (Fig. 6h). Among clusters of exhausted cells, C9 expressed intermediate FOXP3 (at a level comparable to quiescent Treg cells, C12), and proteins encoding genes upregulated in Tconv clonally overlapping with effector Treg cells (Fig. 6c, f). Consistent with scRNA/TCR-seq analysis, C9 and C11 Tconv cells were observed predominantly in the tumor tissue of some, but not all, patients with BC and NSCLC (Fig. 6i). We confirmed that a subset of Tconv expressed CCR8, CD39, OX40 and GITR by manual gating of flow cytometry data (Fig. 6j). These data demonstrate a clonal relationship between subsets of Tconv and effector Treg cells, potentially suggesting interconversion in the tumor microenvironment.

## Discussion

By compiling an atlas of single-cell gene expression data across nine different tumor types and four different tissues, we uncovered several aspects related to the biology of Treg cells in human cancer.

First, we identified universal overexpression of multiple genes that we define as the core signature of intratumoral Treg cells. These genes are uniformly overexpressed when compared to healthy, tissue-resident Treg cells, with the notable exception of Treg cells from the skin and the intestine. Among these genes, we identified surface receptors and transcription factors previously associated with Treg-cell function, such as BATF, Helios and Eos (encoded by *IKZF2* and *IKZF4*, respectively), but also less recognized markers, such as the receptors CD59 and SIRPγ or the metabolic regulators UCP2 or PKM. Specific Helios and Eos inhibitors are currently being tested in early clinical trials. Targeting receptors responsible for specific microenvironmental interactions could provide a promising approach to attenuate Treg cell activity specifically in the tumor. Blockade of CD47, primarily to interfere with SIRPα interaction on myeloid cells, has attracted significant (pre)clinical interest, but the role of SIRPγ on Treg cells or its interaction with CD47 on tumor cells has not been thoroughly studied^46^. Our data also suggest that reinvigoration of antitumor immunity via UCP2 targeting may also have additional effects on Treg cells beyond tumor cell-intrinsic ones that should be tested with dedicated experiments^47^. Thus, targeting these molecules could have a broad benefit for the treatment of human cancers, also in the context of resistance to immune checkpoint inhibitors (ICI), where the 88 gene signature was enriched in intratumoral Treg cells from non-responder patients. Further studies are necessary to identify specific targets among the 88 genes that could be leveraged for cancer immunotherapy. Technologies, such as bispecific antibodies, could be further used to bind multiple Treg cell molecules at the same time, and to enhance specificity and efficiency of Treg cell depletion^4,48^.

Second, we extended the definition of Treg cell heterogeneity beyond known subsets of CCR8^+^ICOS^+^4-1BB^+^IL1R1^+^ effector and quiescent cells^11,15,19,49^. In the nine epithelial tumors analyzed, we reported the presence of CD161^+^ “unstable” Treg cells expressing the proinflammatory transcription factors *RORC* and *TBX21*, producing IL-2, IFN-γ, and TNF, and whose abundance is positively associated with better NSCLC prognosis. These cells are reminiscent of CD161^+^ Treg cells, originally reported in the synovial fluid of pediatric patients with inflammatory arthritis and capable of producing IL-17 and IFN-γ^50^, and of intratumoral “fragile”, IFN-γ-secreting Treg cells, that facilitated tumor regression in mice^51,52^. Similarly, FOXP3^lo^ Treg cells, previously observed in patients with CRC, were equally characterized by higher production of proinflammatory cytokines compared to standard FOXP3^hi^ Treg cells, had limited suppressive capacity, and their abundance correlated with improved survival^53^. The CD161^+^ Treg cells identified here in NSCLC displayed suppressive capacity comparable to CCR7^+^ quiescent and intermediate Treg cell subsets, and expressed effector Treg cell-related molecules such as TIGIT, CD39, OX40, and GITR, possibly suggesting that CD161^+^ Treg cells may differ across tumor types. At the same time, it is possible that the standard in vitro suppression assay may not fully reflect the “proinflammatory” features these cells might exert in the tumor microenvironment under specific local stimuli. By scTCR-seq, we identified limited, but detectable clonal overlap between CD161^+^ (*KLRB1*^+^) and CCR8^+^ effector Treg cells. Trajectory analysis further supported such a dynamic relationship, suggesting that CCR8^+^ effector Treg cell reprogramming towards the CD161^+^ (*KLRB1*^+^) state might be achieved to inflame the tumor microenvironment, as suggested by recent preclinical studies in mice^54–56^. Further studies would thus be needed to better characterize CD161^+^ (*KLRB1*^+^) cells, especially in terms of the precise signals driving their differentiation and fate in tumors.

Further experiments are also required to define the precise identity of the fourth subset of intermediate Treg cells identified in our study. Additional heterogeneity may also emerge in specific conditions, such as following treatment with anti-PD-1/PD-L1 antibodies, where a subset of *MKI67*^+^ (encoding Ki-67) proliferating Treg cells exhibiting effector features was further distinguished in NSCLC^17^. Although our study could be biased by the sample size affecting the identification of differentially expressed genes, we also observed differences in the transcriptome of Treg cells across the nine studied tumors, as well as different effector Treg subsets existing in breast and lung tumors, suggesting further adaptation to specific tissues and disease. In this regard, Treg cells in NSCLC were enriched in CXCR6^hi^ cells, suggesting a response to chemokine CXCL16, previously implicated in resident CD8^+^ T cell infiltration into lung tumors^57^.

Third, we found that effector Treg cells in tumors are clonally related to those in LNs rather than to other intratumoral Treg cell subsets. ICOS^+^PD-1^+^ Treg cells were shown to reside in human metastatic LNs^27^, and inhibition of lymphocyte egress from LNs with FTY720 in a mouse model of melanoma treated with anti-PD-1 decreased Treg cell numbers inside the tumor^22^. Recent work (released as a preprint) has identified a discrete *CXCL13*-expressing “follicular regulatory T cell” subset, residing in germinal center-like niches in breast cancer draining LNs, that is clonally related to effector Treg or *CXCL13*-expressing Tconv cells in tumors^58^. Consistently, also in our study, LN Treg cells clonally related to effector tumor Treg cells overexpressed *CXCL13* and effector Treg cell genes, but not naïve-related genes compared to other Treg cells in LNs, possibly suggesting an ongoing process of maturation that initiates in LNs and concludes in tumors. Genetic tracing tools in vivo in mice will be required in the future to confirm this hypothesis.

The signals at the basis of Treg cell activation in tumor-draining LNs are still to be elucidated, but identification of phenotypic differences between Treg cells from metastatic and non-metastatic LNs suggests that the presence of metastatic tumor cells is crucial^27^. Effector Treg cell gene expression is dependent on TCR signaling^15,59^, thus self-antigens or tumor neoantigens, recently shown in human melanoma to prime and expand tumor-infiltrating Treg cells^25,60^, could be involved in this regard. Additionally, studies in a mouse TRAMPC1 prostate tumor model revealed that TCF1^+^ stem-like CD4^+^ T cells in tumor-draining LN can differentiate into various lineages, including the Treg cell lineage, in response to Treg cell-polarizing conditions^61^. LNs have been recently identified as a tissue source of stem-like progenitor CD8^+^ T cells that sustain long-term antitumor immunity upon checkpoint blockade inhibiton^62,63^. Treg cells may thus behave in a similar fashion according to which LN precursors are recruited to replenish the effector Treg cell pool^22^ in response to effector Treg cell-depleting therapies, such as with anti-CCR8 antibodies. Future studies will have to address whether these dynamics might contribute to possible mechanisms of resistance to novel cancer immunotherapy strategies involving Treg cell depletion^64,65^.

Finally, we reported that a clonal relationship between a subset of Tconv and effector Treg cells can occur in the majority of patients with NSCLC, with a varying frequency of overlap, possibly depending on tumor characteristics, such as neoantigen load^60^. These Tconv and Treg cells shared similar gene expression and phenotypic identity except for the expression of high FOXP3 and Helios by flow cytometry, thereby suggesting that Tconv-Treg cell conversion is potentially possible in tumors. Our results may also suggest an alternative interpretation, i.e., that uncommitted precursors differentiate into either Tconv or Treg cell subsets depending on the tumor microenvironmental conditions. As a fraction of Treg and Tconv cells can naturally share TCR sequences^66^ or recognize the same tumor neoantigens^60^, the dynamics and directionality of this process will require further studies, e.g., using fate-mapping experiments in mice. As Helios usually marks thymus-derived, self-antigen-specific Treg cells, the lack of Helios could thus identify foreign antigen-specificity, including neoantigen-specific Treg cells^25,60^. We observed a higher abundance of Helios^–^ effector Treg cells in NSCLC compared to BC, in accordance with a higher mutational burden found in NSCLC^67^.

In summary, we provide a high-resolution definition of intratumoral Treg cells in human cancers across several tissue sites. This is emphasized by the recent findings that Treg cells may be involved in the response or resistance to immune checkpoint blockade in humans^21–23^. CCR8^+^ Tregs are currently being targeted for depletion in early-phase clinical trials, showing evidence of antitumor activity in some trials^64,65^. The clonal and differentiation characteristics refined here will help to elucidate Treg cell dynamics occurring upon Treg cells depletion and contribution to combination therapy resistance.

## Methods

### Study approval and patient samples

Experimental procedures were performed in accordance with the ethical standards of the institutional and national research committees and with the Declaration of Helsinki (1964). Informed consent was obtained from all patients. Buffy coats from healthy donors were collected and used under approval by Humanitas Clinical and Research Center Institutional Review Board (approval no. 27/11/2020). The use of human samples from patients with NSCLC and BC was approved by the Humanitas Research Hospital Institutional Review Board under protocols 1506 and 2578 (NSCLC) and ONC-OSS-02-2017 (BC). Human samples were collected at IRCCS Humanitas Research Hospital and destroyed following analysis. A total of 45 treatment-naive patients with cancer (30 female and 15 male) were included in the study, aged 39–81 years old and used for flow cytometry and functional analyses. When available, tumor subtype information (LUAD for NSCLC, luminal A/B/TNBC and HER2 status for BC) for studied patients were reported in the source data file.

### Cell isolation and storage

Blood samples of patients with breast and lung cancer were collected into Vacutainer EDTA Tubes (BD), and peripheral blood mononuclear cells (PBMCs) were obtained by density gradient centrifugation using Lympholyte H (Cederlane), at 400×*g*, 30 min, RT^68^. Tumor, NAT and LNs from patients with BC were processed and digested with 0.32 U/mL Liberase TL, for 30 min at 37 °C^69^. Tumor and NAT samples from patients with NSCLC were processed using gentleMACS dissociator (Miltenyi Biotec) and human Tumor Dissociation kit (excluding enzyme R, Miltenyi Biotec), according to manufacturer’s instructions^68^. Following the obtaining of single-cell suspensions, samples were cryopreserved in FBS with 10% DMSO and stored in liquid nitrogen until staining for flow cytometry.

### Full-spectrum flow cytometry and cell sorting

Flow cytometry was performed as described previously^70^ and according to guidelines of the International Society for Advancement of Cytometry^71^. In brief, cryopreserved samples were thawed into complete RPMI 1640 medium, supplemented with 20 μg/ml DNase (Sigma-Aldrich) and further stained. Dead cells were excluded based on Zombie Aqua fixable viability dye (BioLegend) staining. Fluorochrome-labeled monoclonal antibodies from commercial vendors were used to stain surface and intracellular markers (Supplementary Data 8). All antibodies were titrated prior to the experiment to define optimal concentration. Staining of intracellular epitopes was performed using eBioscience Foxp3/Transcription Factor Staining Buffer set (Invitrogen), according to the manufacturer’s instructions. All samples were acquired using spectral mode on BD FACS Symphony A5 SE cytometer with FACS Diva Software Version 9.9 (BD Biosciences), equipped with five lasers and 50 detectors. Machine underwent daily standardization using Rainbow Calibration Particles (eight peaks; BD Biosciences), to avoid batch effect and establish reliable analysis of independent experiments by computational approaches. BD CompBeads, single-stained with fluorescent antibodies, were used as reference controls for spectral unmixing. FCS 3.0 files were imported into FlowJo version 10.8.1 (FlowJo) and further analyzed. Cell sorting was performed using a FACSAria III cell sorter (BD), using a 70-μm nozzle set at 4 °C. Gating strategies for specific FACS sorting experiments are shown in Supplementary Figs. 5a, b and 6a.

### Unsupervised analysis of flow cytometry data

FCS 3.0 files were preprocessed in FlowJo and went through quality control. Spectral unmixing and compensation were first checked and adjusted, if needed. Further preprocessing included removal of cell doublets, aggregates, dead cells and pre-gating on CD3^+^CD4^+^ events (to cluster total CD4^+^ T cells) and CD3^+^CD4^+^CD25^hi^CD127^lo^FOXP3^+^ events (to cluster Treg cells). Data were biexponentially transformed for optimal marker resolution. Each sample was downsampled using the DownSample plug-in in FlowJo, up to 1000 events, and further exported as.csv file and imported into the CRUSTY platform^43^. Different clustering resolutions using the FlowSOM algorithm^72^ were tested to obtain optimal clustering of cells.

### In vitro suppression assay

Human T cells were cultured in RPMI 1640 medium (Euroclone), supplemented with 10% FBS, 100 U/ml penicillin, 100 μg/ml streptomycin and 2 mM L-glutamine (hereafter referred to as R10), unless indicated otherwise. For suppression assays, cryopreserved NSCLC infiltrating tumor leukocytes were subjected to 40:70% discontinuous Percoll gradient centrifugation (790×*g*, 30 min at RT), stained and intratumoral Treg cell subsets were FACS sorted according to the gating strategy in Supplementary Fig. 5a. Autologous responder T cells (conventional CD4^+^CD25^-^ T cells) were FACS sorted from cryopreserved PBMC samples (Supplementary Fig. 5b) and labeled with Cell Trace Violet (CTV) proliferation dye (Invitrogen), according to the manufacturer’s protocol. Suppression assay cultures were performed in 96 U-bottom well plates (coated with 5 μg/ml anti-CD3 (OKT-3 clone; BioLegend)) with anti-CD28 antibodies (1 μg/ml final concentration; BioLegend) and IL-2 (50IU/ml, Miltenyi Biotech), at a 1:1 Treg:Tresp ratio, for 96 h, followed by flow cytometry analysis. Data were analyzed in FlowJo software (BD Biosciences). Suppressive activity was calculated as a percentage (%) of suppression, according to the formula: (1-% proliferating Tresp cultured with Treg/% proliferating Tresp cultured alone)*100.

### Intracellular cytokine analysis

Intratumoral Tconv and Treg cell subsets were FACS sorted from cryopreserved NSCLC-infiltrating tumor leukocytes, following purification by 40:70% discontinuous Percoll gradient centrifugation (790×*g*, 30 min at RT), according to the gating strategy in Supplementary Fig. 5a. Following sorting, cells were restimulated in R10 medium with 10 ng/ml PMA and 500 ng/ml ionomycin for 3 h, in the presence of Golgi Plug/Golgi Stop (concentrations recommended by the manufacturer). Intracellular staining of cytokines and transcription factors was performed using the eBioscience Foxp3/Transcription Factor Staining Buffer set (Invitrogen) (Supplementary Data 8), according to the manufacturer’s protocol, followed by flow cytometry and analysis in FlowJo (BD Biosciences).

### Treg differentiation assays

Treg cells were first selected from healthy donor PBMCs by immunomagnetic selection using EasySep™ Human CD4^+^CD127^low^CD25^+^ Regulatory T Cell Isolation Kit (StemCell Techologies) and further sorted as non-naïve, quiescent CCR7^+^CD45RA^-^ (Supplementary Fig. 6a). 50 × 10^3^ cells were plated onto 96 U-bottom well plates (coated with 5 μg/ml anti-CD3 (OKT-3 clone; Biolegend)), with anti-CD28 antibodies (1 μg/ml final concentration; Biolegend) and IL-2 (50IU/ml, Miltenyi Biotech) in R10 medium mixed 50:50 with conditioned medium from NSCLC cell line A549 (ATCC CCL-185). After 4 days, cells were further replated under the same conditions. On day 0 (following isolation and sorting), day 2 and day 6, cells were stained and analyzed by flow cytometry. For unnsupervised analysis, using the PhenoGraph algorithm^73^ as described above, Treg cells were gated as CD4^+^CD25^+^FOXP3^+^. (Supplementary Fig. 6b).

### Single-cell RNA-sequencing (scRNA-seq) data retrieval and preprocessing

Publicly available single-cell RNA-seq datasets were retrieved from the GitHub repository (https://github.com/ncborcherding/utility/)^74^ and selected based on cancer type and tissue of origin. Specifically, we included the following GEO/ENA datasets: GSE114724^75^ (breast cancer), GSE121636^76^ and PRJNA705464^77^ (renal cancer), GSE145370^78^ (esophageal cancer), GSE162500^79^ (lung cancer), and GSE139555^80^ (multiple tumor types; including colorectal, endometrial and renal cancers), GSE145370^78^ (head and neck squamous cell carcinoma, HNSCC), GSE164522^81^ (colorectal cancer), and GSE212217^82^ (endometrial cancer). Additionally, we incorporated two external datasets not listed in the repository: (i) GSE171899^19^, a multimodal single-cell dataset of intrahepatic cholangiocarcinoma (iCCA) and (ii) a hepatocellular carcinoma (HCC) dataset^83^ available via the online portal accessible at: http://cancer-pku.cn:3838/HCC/. To provide non-malignant reference conditions, healthy/normal and inflammatory settings datasets were integrated. Specifically, healthy Treg cells were derived from the Tabula Sapiens resource^32^ after restricting the analysis to cells from blood, large/small intestine, fat, lung, lymph node, skin, spleen and thymus, as well as from ref. ^31^ (GSE206507), for cells from blood, bone marrow, spleen, lymph nodes, skin, jejunum and lung. In addition, a GSE206298 dataset^33^ was included for comparative integration analyses of patients developing immune checkpoint inhibitor-related colitis. For all included studies, raw data were provided as Seurat objects and were converted and processed using Scanpy (v1.9.6). Metadata such as tissue type, cancer classification, and patient identifiers were retrieved from the original studies or repository annotations.

### Batch-effect correction and unsupervised clustering

All CD45⁺ cells were merged across samples and processed using Scanpy (v1.9.6)^84^. To reduce batch effects prior to clustering, we retained 18,226 genes detected across all datasets. Count matrices were normalized using sc.pp.normalize_total(adata, target_sum = 1e4) and log-transformed with sc.pp.log1p. Highly variable genes (HVGs) were identified using sc.pp.highly_variable_genes with parameters min_mean=0.0125, max_mean = 3, and min_disp = 0.25. Principal component analysis (PCA) was performed with sc.tl.pca using svd_solver = “arpack”, and the top components were retained for downstream analysis. Batch-effect correction was carried out using the Harmony integration algorithm (v0.4.2) (sce.pp.harmony_integrate) with “Tissue” and “Sample” as batch keys^29^. A shared nearest-neighbor graph and UMAP embedding were generated from the Harmony-corrected PCA space, and clusters were identified using the Leiden algorithm. Cell identities were assigned using a hierarchical CellTypist (v1.6.0)-based strategy^85^. Specifically, the Immune_All_High model was first applied to annotate broad immune lineages and identify the T cell population. T cells were then re-clustered and re-annotated using the Immune_All_Low model to achieve finer resolution of T cell subtypes. On this basis, cells annotated as Tem/Trm cytotoxic T cells, Tem/Temra cytotoxic T cells, Trm cytotoxic T cells, and Tcm/Naive cytotoxic T cells were grouped as CD8⁺ T cells, whereas cells annotated as Regulatory T cells, Tcm/Naive helper T cells, Type 1 helper T cells, and Type 17 helper T cells were grouped as CD4⁺ T cells. CD3⁺CD4⁺ T cells were extracted and subjected to a first round of subclustering using DESC (Deep Embedded Single-cell RNA-seq Clustering)^30^ with the following parameters: tol = 0.005, n_neighbors = 10, batch_size = 256, learning_rate = 200. Clustering was performed over a range of Louvain resolutions (0.2–1.2), and resolution 0.6 was selected to define major CD4⁺ T cell subtypes. Treg cells were isolated from the annotated CD4^+^ T cell compartment and re-analyzed independently to resolve finer transcriptional heterogeneity within this population. For this subset-specific analysis, highly variable genes were recalculated, dimensionality reduction was repeated, and a new latent representation was learned using DESC (v2.1.2). Treg cells were then clustered using DESC at a resolution of 0.5. Additional DESC runs at different resolution parameters were used to assess the stability of the inferred Treg substructure (Supplementary Fig. 4b). To investigate the transcriptional identity of Treg cell states in an independent cohort with paired TCR data, we used our integrated dataset as a reference to annotate the dataset from ref. ^34^ dataset via Seurat’s FindTransferAnchors function (v3.2.0)^86^. This approach enabled the transfer of our cluster labels to the Caushi et al. dataset, thereby leveraging its TCR information to explore clonal features associated with predicted Treg cell populations (cluster 5).

### Signature enrichment and AUC score calculation

Gene-signature enrichment was performed at the single-cell level using functions implemented in the omicverse package^87^. Predefined gene sets (88 core Treg gene signature; signature of suppressive molecules) representing the transcriptional programs of interest were scored across individual cells to quantify signature activity within the integrated datasets. The resulting enrichment scores were used to compare cell populations and biological conditions. To evaluate the ability of each signature to discriminate the target population from other cells, the area under the curve (AUC) was calculated based on the corresponding signature scores. Higher AUC values indicated stronger performance and greater specificity of the signature for the cell state of interest.

### Recurrence-free survival analysis

To assess the clinical relevance of the identified Treg cell states, recurrence-free survival (RFS) analysis was performed using the single-cell cohort of neoadjuvant chemotherapy/anti-PD-1-treated NSCLC (GSE243013)^17^. Treg cells were extracted according to annotations of the original study, and the identity of the four Treg cell clusters (Fig. 2a) was annotated via Seurat’s FindTransferAnchors function^86^. For each sample, the relative abundance of individual Treg cell subclusters was calculated as the proportion of cells within the total Treg cell compartment. These sample-level measurements were integrated with clinical annotations containing follow-up time (RFS_months_new) and recurrence status (RFS_status_new). Kaplan–Meier survival curves were generated using the lifelines Python package after stratification of samples into low- and high-abundance groups based on median cutoffs, and statistical significance was evaluated using the log-rank test. Cox proportional hazards regression was additionally applied to estimate the relationship between cell-state abundance and recurrence risk. To correlate subset abundance, pairwise Pearson correlation was performed.

### Transcriptional trajectory analysis

Lineage inference and pseudotime analysis were performed using the Slingshot framework through its Python implementation, pyslingshot (v0.2.0)^39^. Slingshot infers global lineage structure from cluster relationships embedded in a reduced-dimensional representation of the data and assigns each cell a pseudotime value along the inferred trajectories. In our analysis, low-dimensional coordinates from the integrated single-cell object, together with cell-cluster annotations, were provided as input to reconstruct trajectories across transcriptionally related T cell states. This approach enabled the identification of putative transitional paths and the ordering of cells along continuous differentiation or activation axes. Pseudotime-dependent changes were subsequently used to interpret dynamic transcriptional programs associated with cell-state progression.

To further reconstruct differentiation trajectories and explore the transcriptional dynamics of CD4⁺ Treg cells, pseudotime analysis using the STREAM algorithm (v1.1)^40^ was performed. Gene expression data were first normalized based on library size using st.normalize with method = “lib_size”, then log-transformed using st.log_transform. Mitochondrial genes were removed from the analysis using st.remove_mt_genes. Dimensionality reduction was performed with Modified Locally Linear Embedding (MLLE) using st.dimension_reduction with method = “se” (spectral embedding), selecting the top variable genes (feature = “var_genes”) and projecting the data into three dimensions using 15 neighbors. Trajectory inference was carried out using the Elastic Principal Graph (ElPiGraph) approach with parameters epg_alpha = 0.01, epg_mu = 0.05, and epg_lambda = 0.05, allowing robust reconstruction of complex branching structures in the transcriptional landscape. The resulting principal graph was used to assign pseudotime values and infer branching events. Cells were projected onto the tree structure to assess their position along the inferred differentiation paths. This approach enabled visualization of the transitions between transcriptional states and identification of putative lineage bifurcations within the CD4⁺ Treg cell compartment. Transition and diverging genes associated with distinct branches were explored along the pseudotime axis using STREAM’s internal visualization functions.

### SCENIC analysis

Transcription factor activity was inferred using the single-cell regulatory network inference and clustering (SCENIC) framework^41^, implemented via the Python-based pySCENIC (v0.12.1) within the Docker container. The analysis was performed on the integrated CD4⁺ Treg cells to identify active regulons across transcriptional states. Gene regulatory networks were inferred using the grn command with the GRNBoost2 algorithm, which identifies co-expression relationships between transcription factors and potential target genes. The input was a normalized expression matrix and a list of human transcription factors. Motif enrichment and regulon refinement were then performed using the ctx module. Two cis-regulatory motif ranking databases were used for the human genome: hg19-tss-centered-5kb-7species and hg19-tss-centered-10kb-7species. Only targets with enriched transcription factor-binding motifs were retained to define direct regulons. Regulon activity scores were then computed at single-cell resolution using the AUCell module. This yielded an AUCell score matrix indicating the activity of each regulon across individual cells. To identify celltype–specific regulators, Z-scores were calculated by standardizing the mean AUCell score of each regulon in a given cell type relative to the global mean and standard deviation of that regulon’s activity across all cells. Regulons with Z-score ≥3.0 were considered specifically enriched in the corresponding population. The Z-score matrix was visualized using a heatmap generated in Python with the Seaborn package (sns.heatmap), with regulons as columns and cell types as rows.

### Pathway and gene ontology enrichment analysis

Pathway and gene ontology enrichment analysis was performed using g:Profiler^88^. Gene sets of interest from each cluster were input as gene symbols, and enrichment was assessed using Reactome gene sets. The statistical significance of enrichment was evaluated using the built-in g:SCS correction (set size-adjusted multiple testing correction method), and adjusted *P* values were used for filtering. For each cluster, the top enriched terms were identified, and a curated subset of biologically relevant terms was selected for visualization. Enrichment values were visualized as −log10(adjusted *P* value), with a significance threshold corresponding to −log10(0.05). Only terms passing this threshold were retained for downstream interpretation and plotting.

### Single-cell TCR sequencing (scTCR-seq) analysis

T-cell receptor (TCR) sequencing data were analyzed using the Scirpy package (v0.11.2)^89^. Quality control of receptor chains was performed using ir.tl.chain_qc, and chain pairing status was assessed by plotting group abundance across tissue types (ir.pl.group_abundance). Cells lacking productive receptor information or containing ambiguous chain pairing annotations (e.g., no IR, orphan VDJ, orphan VJ, extra VDJ, extra VJ) were excluded from downstream analysis. Clonotypes were defined using the amino acid sequence of the CDR3 region. Initial clonotype assignment was performed with ir.tl.define_clonotypes using the receptor_arms = “all“ and dual_ir = “primary_only” settings. To identify clonotype clusters based on sequence similarity, pairwise distances were calculated using the alignment metric (ir.pp.ir_dist), followed by clustering with ir.tl.define_clonotype_clusters. Clonal expansion was visualized across tissues and clusters using ir.pl.clonal_expansion, with a clip_at threshold of 4 to cap outliers. Plots were customized using Matplotlib to generate high-resolution figures, including a summary of clonal expansion by tissue type. Repertoire overlap between tissues (e.g., lymph nodes and tumor) was assessed with ir.pl.repertoire_overlap. To evaluate clonal sharing across tissues, an UpSet plot was constructed using the upsetplot Python library (v0.9)^90^.

### TCR reconstruction from bulk RNA sequencing and clonotype overlap analysis

T cell receptor (TCR) sequences were reconstructed from bulk RNA-seq data using TRUST4 (v1.0.13), a tool designed for de novo assembly of TCR and BCR contigs from unselected transcriptomic datasets^91^. TRUST4 identifies and assembles full-length V(D)J recombination products, including CDR3 regions, and maps them to the IMGT reference database to determine V, D, and J gene usage. Paired-end FASTQ files from sorted CD4⁺ Treg cell subsets (CCR8⁺ICOS⁺ and CCR8⁻ICOS⁻ populations) from ref. ^15^ were processed using run-trust4 with the hg38_bcrtcr.fa genome and IMGT + C.fa annotation. TCR repertoires were parsed and analyzed in R using the immunarch package (v0.6.7). TRUST4 output was loaded using repLoad with.mode = “single”, and clonotypes associated with immunoglobulin chains were filtered out using repFilter to retain only T cell–specific clonotypes. To assess inter-sample clonal sharing, we implemented a custom Python script inspired by the “Shared clonotype abundance plot” from VDJtools. Amino acid CDR3 sequences shared between two samples were identified and ranked by abundance. The top shared clonotypes were visualized individually, with all remaining shared clonotypes aggregated into a single NotShown category, and nonoverlapping (private) clonotypes grouped into a NonOverlapping category. Clonotype CDR3 amino acid sequences were plotted along the x-axis, ordered by the sample in which each clone reached its maximum abundance. Clone frequencies were visualized as stacked bars, scaled by the number of supporting reads per sample. Colors were assigned using a curated palette to distinguish clonotype groups. This approach allowed for detailed visualization of highly expanded public TCRs while maintaining context about the overall repertoire composition, highlighting clonal convergence and private expansions between T cell subsets.

### Reporting summary

Further information on research design is available in the Nature Portfolio Reporting Summary linked to this article.

## Supplementary information

Supplementary Information

Description of Additional Supplementary Files

Supplementary Data 1

Supplementary Data 2

Supplementary Data 3

Supplementary Data 4

Supplementary Data 5

Supplementary Data 6

Supplementary Data 7

Supplementary Data 8

Supplementary Data 9

Supplementary Data 10

Supplementary Data 11

Supplementary Data 12

Supplementary Data 13

Supplementary Data 14

Reporting Summary

Transparent Peer Review file

## Source data

Source Data

## Data Availability

Publicly available single-cell RNA-sequencing datasets were retrieved from the GitHub repository [ttps://github.com/ncborcherding/utility]. Original single-cell RNA-sequencing datasets used in this study are available in the Gene Expression Omnibus database under accession codes: GSE114724, GSE121636, GSE145370, GSE162500, GSE139555, GSE145370, GSE164522, GSE212217, GSE171899, GSE206507, GSE206298, and GSE243013; in the NCBI BioProject database under accession code: PRJNA705464; online in the Tabula Sapiens resource: https://tabula-sapiens.sf.czbiohub.org. The publicly available bulk RNA-sequencing dataset used in this study is available in the Gene Expression Omnibus database under accession code GSE128822. The publicly available single-cell TCR-sequencing dataset used in this study is available in the Gene Expression Omnibus database under accession code GSE176022. The remaining data are available within the Article, Supplementary Information or Source Data file. Source data are provided with this paper.

## Code availability

All custom codes used in this study, including preprocessing, clustering, annotation, trajectory inference, and TCR analysis, have been deposited on GitHub and are available at https://github.com/luglilab/Treg-diversity-in-human-tumors-.
