# Supplementary material for: Molecular heterogeneity and clonal origin of CCR8+ effector regulatory T cells in human cancer
Source: Nat Commun. 2026 Aug 21;17:9994. doi: 10.1038/s41467-026-76670-6 (PMC13590533; doi:10.1038/s41467-026-76670-6)
Supplement: Supplementary file 1 [file 41467_2026_76670_MOESM17_ESM.pdf]

nature portfolio

Corresponding author(s): Enrico Lugli
Julian Swatler

Last updated by author(s): Jul 24, 2026

Reporting Summary

Nature Portfolio wishes to improve the reproducibility of the work that we publish. This form provides structure for consistency and transparency in reporting. For further information on Nature Portfolio policies, see our Editorial Policies and the Editorial Policy Checklist.

Statistics

For all statistical analyses, confirm that the following items are present in the figure legend, table legend, main text, or Methods section.

n/a	Confirmed	
☐	☒	The exact sample size (*n*) for each experimental group/condition, given as a discrete number and unit of measurement
☐	☒	A statement on whether measurements were taken from distinct samples or whether the same sample was measured repeatedly
☐	☒	The statistical test(s) used AND whether they are one- or two-sided<br>*Only common tests should be described solely by name; describe more complex techniques in the Methods section.*
☒	☐	A description of all covariates tested
☐	☒	A description of any assumptions or corrections, such as tests of normality and adjustment for multiple comparisons
☐	☒	A full description of the statistical parameters including central tendency (e.g. means) or other basic estimates (e.g. regression coefficient) AND variation (e.g. standard deviation) or associated estimates of uncertainty (e.g. confidence intervals)
☐	☒	For null hypothesis testing, the test statistic (e.g. *F*, *t*, *r*) with confidence intervals, effect sizes, degrees of freedom and *P* value noted<br>*Give P values as exact values whenever suitable.*
☒	☐	For Bayesian analysis, information on the choice of priors and Markov chain Monte Carlo settings
☒	☐	For hierarchical and complex designs, identification of the appropriate level for tests and full reporting of outcomes
☒	☐	Estimates of effect sizes (e.g. Cohen's *d*, Pearson's *r*), indicating how they were calculated

Our web collection on statistics for biologists contains articles on many of the points above.

Software and code

Policy information about availability of computer code

Data collection	Flow cytometry data were collected using BD FACS Symphony SE, equipped with FACS Diva Software Version 9.9 (BD Biosciences).<br><br>scRNA-seq data used in this study were retrieved from publicly available repositories.

Data analysis	Flow cytometry: Flow cytometry standard (FCS) 3.0 files were analyzed with FlowJo (BD) version 10.8.1. Unsupervised analyses were performed using CRUSTY web platform. Clusters were determined by FlowSOM or PhenoGraph algorithm.<br><br>scRNASeq: Single-cell RNA sequencing analysis was performed using Scanpy (v1.9.6). Dataset integration was carried out with DESC (v2.1.2) and Harmony (v0.4.2). Cell type classification was conducted with CellTypist (v1.6.0), while trajectory inference was performed using Slingshot (v0.2.0) and STREAM (v1.1). Gene regulatory network reconstruction and transcription factor activity inference were performed with pySCENIC (v0.12.1). Enrichment analysis was performed using g:Profiler online version.<br><br>TCR-Seq: Bulk TCR sequencing data were processed with TRUST4 (v1.0.13). Single-cell TCR sequencing analysis was performed using Scirpy (v0.11.2) in combination with UpSetPlot (v0.9). TCR information was paired with integrated object with Seurat's FindTransferAnchors function (v3.2.0).<br><br>Code been deposited on GitHub and is available at https://github.com/luglilab/Treg-diversity-in-human-tumors-

For manuscripts utilizing custom algorithms or software that are central to the research but not yet described in published literature, software must be made available to editors and reviewers. We strongly encourage code deposition in a community repository (e.g. GitHub). See the Nature Portfolio guidelines for submitting code & software for further information.

Data

Policy information about availability of data

All manuscripts must include a data availability statement. This statement should provide the following information, where applicable:

- Accession codes, unique identifiers, or web links for publicly available datasets
- A description of any restrictions on data availability
- For clinical datasets or third party data, please ensure that the statement adheres to our policy

Publicly available single-cell RNA-seq datasets were retrieved from the GitHub repository https://github.com/ncborcherding/utility/ and selected based on cancer type and tissue of origin. Specifically, we included the following GEO/ENA datasets: GSE114724 (Breast cancer), GSE121636 and PRJNA705465 (Renal cancer), GSE145370 (Esophageal cancer), GSE162500 (Lung cancer), GSE139555 (Multiple, including Colorectal, Endometrial, and Renal cancers), GSE145370 (Head and Neck Squamous Cell Carcinoma, HNSCC), GSE164522 (Colorectal cancer), and GSE212217 (Endometrial cancer). In addition, two external datasets not listed in the repository were incorporated: (i) GSE171899, a multimodal single-cell dataset of intrahepatic cholangiocarcinoma (ICC), and (ii) a hepatocellular carcinoma (HCC) dataset available via the Cancer-PKU portal, as well as a single-cell RNA + TCR dataset from Caushi et al. (Nature, 2021; PMID: 34290408). Caushi et al. dataset was retrieved directly from the authors. For TCR reconstruction analysis using TRUST4, a dataset from Alvisi et al. (J Clin Invest. 2020, PMID: 32125291, GSE128822) was used.

To provide non-malignant reference conditions, healthy/normal and inflammatory settings datasets were integrated, from the Tabula Sapiens resource, GSE206507 and GSE206298 datasets. To assess the clinical relevance of the identified Treg states, recurrence-free survival (RFS) analysis was performed using the single-cell cohort of neoadjuvant chemotherapy/anti-PD1-treated non-small cell lung cancer (GSE243013).

Source data generated in this study are provided with this paper.

Research involving human participants, their data, or biological material

Policy information about studies with human participants or human data. See also policy information about sex, gender (identity/presentation), and sexual orientation and race, ethnicity and racism.

Reporting on sex and gender	A total of 45 treatment-naive cancer patients (30 female, 15 male) were included in the study, aged 39-81 years old and used for flow cytometry analyses.<br>Buffy coat samples were collected as anonymous, therefore it is impossible to recover personal information.<br>Sex was not considered as a variable in the study.
Reporting on race, ethnicity, or other socially relevant groupings	Race and ethnicity were not considered in this study.
Population characteristics	Samples from patients with breast cancer (23, female, 39-78 years old) and non-small cell lung cancer (22, 7 female, 15 male, 53-81 years old) were used in the study. No compensation was provided for participation in the study.
Recruitment	Healthy donors (buffy coats, anonymous) were obtained from the Humanitas Clinical and Research Center. Patients with breast and non-small cell lung cancer were obtained from Humanitas Clinical and Research Center. No compensation was provided for participation in the study. No self-selection bias or other biases of the sample collection or choice for analysis was present.
Ethics oversight	Humanitas Clinical and Research Center IRB approved sample and data collection. Buffy coats from healthy donors were collected and used under approval by Humanitas Clinical and Research Center Institutional Review Board (approval no. 27/11/2020). Breast cancer and lung cancer specimens were collected under approvals ONC/OSS-02/2017 and 2578, respectively, following written informed consent and in accordance with Declaration of Helsinki.

Note that full information on the approval of the study protocol must also be provided in the manuscript.

Field-specific reporting

Please select the one below that is the best fit for your research. If you are not sure, read the appropriate sections before making your selection.

- [x] Life sciences
- [ ] Behavioural & social sciences
- [ ] Ecological, evolutionary & environmental sciences

For a reference copy of the document with all sections, see nature.com/documents/nr-reporting-summary-flat.pdf

Life sciences study design

All studies must disclose on these points even when the disclosure is negative.

Sample size	No statistical methods were used to calculate sample size, but these are similar to those reported in previous studies of the Laboratory.
Data exclusions	For flow cytometry experiments in Fig. 1e, only samples from patients with collected each blood, normal adjacent tissue (NAT) and tumor were considered to performed paired comparative analyses. 1 patient was excluded as only 1 Treg event in a NAT sample was acquired by flow cytometry.<br><br>In flow cytometry experiments in Fig. 2f, 4 and 6e-i, Treg were subclustered or gated into multiple populations. 3 samples of normal adjacent tissue (NAT) from breast cancer patients were excluded as less than 50 Treg events were acquired by flow cytometry.
Replication	Flow cytometry samples were acquired in 5 different batches. Flow cytometer has undergone standarization to establish stability across batches, further verified by comparing same samples of healthy donor PBMCs stained across experiments. Wet lab experiments were performed according to standardized protocols, antibody staining consistency was confirmed across lots and optimized by titration for each lot. All experimental replicates were successful and no experiments were excluded.
Randomization	Patients samples were randomly allocated for flow cytometry staining.
Blinding	N/A, all methods used were absolute measures by machines and not subjective to human error.

Reporting for specific materials, systems and methods

We require information from authors about some types of materials, experimental systems and methods used in many studies. Here, indicate whether each material, system or method listed is relevant to your study. If you are not sure if a list item applies to your research, read the appropriate section before selecting a response.

Materials & experimental systems

n/a	Involved in the study	
[ ]	[x]	Antibodies
[x]	[ ]	Eukaryotic cell lines
[x]	[ ]	Palaeontology and archaeology
[x]	[ ]	Animals and other organisms
[ ]	[x]	Clinical data
[x]	[ ]	Dual use research of concern
[x]	[ ]	Plants

Methods

n/a	Involved in the study	
[x]	[ ]	ChIP-seq
[ ]	[x]	Flow cytometry
[x]	[ ]	MRI-based neuroimaging

Antibodies

Antibodies used

B346965 Flow cytometry:
Name and fluorophore Clone Company Catalog Host Lot Dilution
hCCR8 BV421 433H BD Biosciences 566379 mouse _0049998 1.25 µl in 100 µl
hCCR7 BB660 150503 BD Biosciences 625454 mouse _0265641 1.25 µl in 100 µl
hCXCR6 R718 13B 1E5 BD Biosciences 751975 mouse 3051044 2.5 µl in 100 µl
hCD3 BUV496 UCHT1 BD Biosciences 612940 mouse 1347983 1.25 µl in 100 µl
hCD4 BUV615 SK3 BD Biosciences 612987 mouse 2202199 0.3 µl in 100 µl
hCD8 BV750 RPA-T8 BD Biosciences 747385 mouse 3051007 0.08 µl in 100 µl
hCD25 RB780 2A3 BD Biosciences 568688 mouse 2259586 1.25 µl in 100 µl
hCD127 eFluor450 eBioRDR5 Invitrogen 48-1278-42 mouse 2402903 2.5 µl in 100 µl
hTIGIT BV650 741182 BD Biosciences 747840 mouse _0337002 1.25 µl in 100 µl
hGITR BV480 V27-580 BD Biosciences 747658 mouse 2270530 1.25 µl in 100 µl
hOX40 BUV805 ACT35 BD Biosciences 749285 mouse 3051033 2.5 µl 100 µl
hCTLA-4 BUV563 BNI3 BD Biosciences 624284 mouse 3054451 1.25 µl in 100 µl
hTNFR2 PE-Dazzle594 3G7A02 Biolegend 358414 rat B366401 2.5 µl in 100 µl
hCD74 BB630 LN2 BD Biosciences 624294 mouse 3054454 1.25 µl in 100 µl
hCD30 APC BerH8 BD Biosciences 563500 mouse 2285357 1.25 µl in 100 µl

hICOS BUV395 DX29 BD Biosciences 564777 mouse 2283298 0.63 µl in 100 µl
hCD161 BV605 HP-3G10 Biolegend 339916 mouse B265242 2.5 µl in 100 µl
hSIRPG BUV661 OX-119 BD Biosciences 750490 mouse 3123871 5 µl in 100 µl
hCD59 BUV737 p282 BD Biosciences 752541 mouse 3051045 0.63 µl in 100 µl
hIL1R2 FITC 34141 Invitrogen MA5-23662 mouse YA3816174 5 µl in 100 µl
hCD70 PE-Cy7 113-16 Biolegend 355112 mouse B374800 1.25 µl in 100 µl
hCD39 BB755 A1/CD39 BD Biosciences 624391 mouse 3054463 0.63 in 100 µl
hIL21R BV786 17A12 BD Biosciences 742422 mouse 3073910 1.25 µl in 100 µl
hTIM-3 PE-Fire810 F38-2E2 Biolegend 345059 mouse B384651 0.63 µl in 100 µl
hHLA-DR PerCP-eFluor710 L243 Invitrogen 46-9952-42 mouse 2410947 2.5 µl in 100 µl
hPD-1 BV711 EH12.1 BD Biosciences 564017 mouse 2265953 2.5 µl in 100 µl
hCD45RO BV570 UCHL1 Biolegend 304226 mouse B361465 0.63 µl in 100 µl
hFOXP3 PE-Cy5.5 PCH101 Invitrogen 35-3776-42 rat 2542089 1.25 µl in 100 µl
hHelios PE-Cy5 22F6 Invitrogen 15-9883-42 armenian Hamster 2487971 1.25 µl in 100 µl
hEos PE W7-486 BD Biosciences 566747 rat 1209110 0.63 µl in 100 µl
hGranzymeB APC-R700 GB11 BD Biosciences 624348 mouse 3054468 0.3 µl in 100 µl
hCD3 BV605 SK7 BD Biosciences 563219 mouse 9014634 1.25 µl in 100 µl
hCD4 FITC M-T477 BD Biosciences 556615 mouse 3297730 1.25 µl in 100 µl
hCD25 BV786 2A3BD Biosciences 741035 mouse 2174176 0.15 µl in 100 µl
hCD127 APC-R700 HIL-7R-M21 BD Biosciences 565185 mouse 7136631 0.63 µl in 100 µl
hCCR7 PE-CF594 562381 BD Biosciences 562381 mouse 5142601 2.5 µl in 100 µl
hCD161 PE-Cy5 HP-3G10 Biolegend 339951 mouse B385311 2.5 µl in 100 µl
hCD25 BV650 M-A251 BD Biosciences 563719 mouse 1098368 1.25 µl in 100 µl
hCD4 BV570 RTA-T4 Biolegend 300534 mouse B346965 2.5 µl in 100 µl
hCD127 PE-Cy5.5 eBioRDR5 Invitrogen 35-1278-42 mouse 2955479 1.25 µl in 100 µl
hCD39 APC-Cy7 A1 Biolegend 328226 mouse B453667 1.25 µl in 100 µl
hGITR BV605 V27-580 BD Biosciences 747664 mouse 5275188 1.25 µl in 100 µl
hOX40 BV711 Ber-ACT35 Biolegend 350030 mouse B452853 5 µl in 100 µl
hCCR7 BV711 G043H7 Biolegend 353228 mouse B257292 2.5 µl in 100 µl
hCD45RA APC-H7 HI-11 BD Biosciences 560674 mouse 3177508 2.5 µl in 100 µl
hCD25 BUV563 2A3 BD Biosciences 612918 mouse 4256301 0.3 µl in 100 µl
h4-1BB PE 4B4-1 Biolegend 309804 mouse B367290 1.25 µl in 100 µl
hCTLA-4 PE-eFluor610 14D3 Invitrogen 61-1529-42 mouse 2383373 0.3 µl in 100 µl
hTNFR2 PE-Cy7 3G7A02 Biolegend 358412 rat B347757 0.63 µl in 100 µl
hIFNg BV711 B27 BD Biosciences 564039 mouse 157427 1.25 µl in 100 µl
hTNF BUV395 MAb11 BD Biosciences 563996 mouse 5076911 1.25 µl in 100 µl
hIL2 APC MQ1-17H12 BD Biosciences 554567 rat 1244250 0.31 µl in 100 µl
hIL10 PE JES3-9D7 BD Biosciences 564049 rat 4185495 2.5 µl in 100 µl
hIL17 PE eBio64DEC17 Invitrogen 12-7179-42 mouse E10705-1634 2.5 µl in 100 µl
hRORgt AF488 Q21-559 BD Biosciences 563621 rat 5067266 2.5 µl in 100 µl
hGATA3 RB705 L50-823 BD Biosciences 570618 rat 5191420 0.63 µl in 100 µl
hT-bet PE-Cy7 eBio4B10 (4B10) Invitrogen 25-5825-82 mouse 2338686 0.3 µl in 100 µl

Validation

Flow cytometry antibodies were validated according to previously described procedures (Brummelman et al., 2019, Nat Protoc). In brief, antibodies went titration using serial dilutions (1:1, 1:2, 1:4, 1:8, 1:16, 1:32, 1:64) and were tested on positive and negative biological controls. Titration included comparison of different lots to establish staining consistency across experiments.

Clinical data

Policy information about clinical studies
All manuscripts should comply with the ICMJE guidelines for publication of clinical research and a completed CONSORT checklist must be included with all submissions.

Clinical trial registration

n/a

Study protocol

n/a

Data collection

Samples from cancer patients were obtained in the operating room in a sterile field by the operating surgeon. Blood samples were collected by venipuncture before anaesthesia induction.

Outcomes

n/a

Plants

Seed stocks	n/a
Novel plant genotypes	n/a
Authentication	n/a

Flow Cytometry

Plots

Confirm that:

- [x] The axis labels state the marker and fluorochrome used (e.g. CD4-FITC).
- [x] The axis scales are clearly visible. Include numbers along axes only for bottom left plot of group (a 'group' is an analysis of identical markers).
- [x] All plots are contour plots with outliers or pseudocolor plots.
- [x] A numerical value for number of cells or percentage (with statistics) is provided.

Methodology

Sample preparation	Cells were collected from patients' samples, processed, frozen and later stained for flow cytometry, following thawing into complete RPMI 1640 media, supplemented with 20 µg/ml DNase. First, samples were stained with ZOMBIE Aqua viability dye (Biolegend, for human samples), for 15 minutes at room temperature, followed by antibody staining. Staining was performed with fluorochrome-conjugated monoclonal antibodies purchased from BD Biosciences, Biolegend, Thermo Fisher Scientific, following titration to obtain optimal staining concentrations. Staining panels and gating strategy were as described in Supplementary Tables and Extended Data Figures. Chemokine receptors were stained first by incubation with antibodies for 30 minutes at 37°C, followed by surface marker staining for 30 minutes at room temperature. Intracellular staining was performed using eBioscience Foxp3/Transcription Factor Buffer Set (Invitrogen/Thermo Fisher), according to manufacturer's instructions. Intracellular staining was performed for 30 minutes at room temperature.
Instrument	Flow cytometry data were collected using BD FACS Symphony SE, equipped with FACS Diva Software Version 9.9 (BD Biosciences). Spectral unmixing was performed using single-stained controls prepared with antibody capturing beads (BD Biosciences). Cell sorting was performed using FACSAria III cell sorter (BD), using 70 µm nozzle.
Software	FlowJo version 10.8.1 was used for analysis of FCS 3.0 files obtained following acquisition. For unsupervised analyses, cell populations of interest were pre-gated (exclusion of cell aggregates, antibody aggregates, dead cells, irregular events) and proper biexponential transformation of axes was performed. Samples were downsampled up to 1.000 events, exported as .csv files and analyzed using CRUSTY online platform (https://crusty.ba.itb.cnr.it), using FlowSOM or PhenoGraph algorithm.
Cell population abundance	n/a
Gating strategy	Gating strategy for all experiments are indicated in detail in Extended Data Figures 4f, 5a,b, 6a,b. For initial gating in all experiments:<br>1. FSC/SSC gate was used to identify lymphocytes.<br>2. FSC-height/FSC-area gate was used to exclude cell doublets/aggregates.<br>3. Time gate was used to ensure signal stability along acquisition.<br>4. Dead cell exclusion was performed.

- [x] Tick this box to confirm that a figure exemplifying the gating strategy is provided in the Supplementary Information.
