# Supplementary material for: Molecular heterogeneity and clonal origin of CCR8+ effector regulatory T cells in human cancer
Source: Nat Commun. 2026 Aug 21;17:9994. doi: 10.1038/s41467-026-76670-6 (PMC13590533; doi:10.1038/s41467-026-76670-6)
Supplement: Supplementary file 2 [file 41467_2026_76670_MOESM18_ESM.pdf]

nature portfolio

Peer Review File

Molecular heterogeneity and clonal origin of CCR8+ effector regulatory T cells in human cancer

Corresponding Author: Dr Enrico Lugli

This file contains all reviewer reports in order by version, followed by all author rebuttals in order by version.

Version 0:

Reviewer comments:

Reviewer #1

(Remarks to the Author)
In this manuscript by J. Swatler et al, the authors perform a comprehensive integration of published scRNAseq data from 9 tumor types in order to identify Treg cell specific signatures that will allow the precise targeting of intratumoral Treg cells, which will hold an enormous therapeutic benefit. Towards this they identified a core signature consisted of 88 genes to be highly expressed gy Treg cells in those tumors and they also describe 4 Treg cells subsets with distinct tissue distribution. The concept is highly novel, since there is an urgent need to identify tumor-specific Treg cell signatures that will allow their ablation which will promote tumor regression but will also facilitate better responses during cancer immunotherapy. Nevertheless, my major concern in this manuscript, is that the authors do not take into account the tissue specific Treg cell signatures that are present in healthy situations. It is well accepted that Treg cells present tissue specific adaptations during homeostasis (https://doi.org/10.1016/j.it.2025.12.001), which I would expect to influence or shape the tumor microenvironment.

Specific comments.
1. How different healthy Treg cells (circulating or tissue resident) are, regarding the core signature, described in this study.
2. What are the suppressive signatures expressed by the 4 Treg cells clusters, via which they may exert their function? How do they contribute to tumor development?
3. Considering the important role of Treg cells in the maintenance of the immune homeostasis but also on their influence in the development of immune-related adverse events, It is very important (similar to comment 1) to describe whether you also have expression of the core signature by naive Treg cells (circulating) and if this signature maybe altered in patients developing irAEs after immunotherapy.
4. In a similar manner, it would be of great interest to analyze whether the presence of the core signature (or another) by Treg cells, may dictate responses to immune checkpoint inhibitors.
5. How do the Authors envision the targeting of the 88 core signature? In other words, in the clinic, what may be the advantages of the 88 core signature compared to targeting of CCR8+ Tregs, in a therapeutic setting?

Reviewer #2

(Remarks to the Author)
Tregs are an immunosuppressive subset of CD4+ T cells that play beneficial roles in normal physiology but are highly deleterious in cancer by blocking effective cancer immunity. Immunotherapies targeting Tregs can be efficacious but often induce dose-limiting toxicities linked to autoimmunity. In order to identify potential markers of tumor-specific Tregs and to better understand their ontogeny, this study focuses on a large cohort of scRNAseq of T cells infiltrating tumors and normal adjacent tissue across 9 tumor types assembled from the literature. The authors perform extensive flow cytometry profiling of an inhouse cohort of lung and breast cancer patients for validation and investigate a public scRNA/TCRseq dataset of TILs from NSCLC to study migration and differentiation at the TCR clone/lineage level.
The data appears to be of high quality and assembling this large cohort across different studies is a valuable and efficient approach. However, the analyses need to be extended significantly to make the conclusions robust and self-consistent and conclusions concerning differentiation pathways should be tested experimentally, eg with sorting and in vitro differentiation.

Major comments

Figure 1b, this clustering and annotation is not fully convincing and I have concerns that more Treg cells might be in the dataset as subsets of clusters other than cluster 3. It is surprising that no cycling cluster is identified. There are 3 clusters annotated only as "undefined" and several others only labelled by a few marker genes as opposed to a formal cell type/cell state. The top filtered marker genes for cluster 10 in supplemental table 1 all look like myeloid genes. Could this be contamination ? Or T:myeloid doublets ?
The definition of Treg marker genes (Fig 1d) should be complemented with all pairwise comparisons of Tregs vs each Tconv subcluster. The current approach of cluster 3 vs everything else can easily miss genes that are in fact highly expressed on minor Tconv subclusters.
Fig 1d, why visualize Z score instead of fold-change or log fold-change ? How exactly was z-score calculated ?
Fig 1f,g. Are patterns of similarity and difference between tumor types related to sample size per tumor type and/or tissue ?
Pages 6/7, lines 174-175 hypothesizes that clustering of Breast and Ovarian Tregs is due to predominance of female patients. Can this be validated with female patients of other tumor types ? ChrX/Y gene expression should be usable to infer sex even if it's not in clinical annotations.
Figure 2h, STREAM analysis to confirm C0 as an intermediate/transitional state seems tenuous. Do other pseudotime/trajectory algorithms validate this ? Relatedly, what if C3 are considered as root (ie as induced/peripheral Tregs) ? The later TCR sharing analysis is also used to address the question of exTregs or induced Tregs but the conclusions are not fully clear to me. Sorting specific populations and ex vivo differentiation would be highly informative.
In general the TCR sharing analyses do not seem to be conditioned on the number of cells per cluster or sample, which can have a large effect when not accounted for.
Fig 4f. It is hard to interpret this figure. It seems like there is a lot of sharing (ie everything except the lightest gray), especially of more expanded clones. Not finding sharing for lowly expanded clones could easily be a power issue.
Fig 5c. Complement this by Tconv vs Treg differential expression volcano plots for the shared TCRs to be sure that the Tconv label is accurate (ie that they aren't misclassified Tregs)

Minor comments
Although Fig 1a is a schematic, it would still be useful to explain what genes/signatures are used to identify T cells amongst CD45+ leukocytes, CD4+ T cells amongst total T cells, and Tregs amongst CD4+ T cells.
Page 5 bottom paragraph clarify if the analysis is at the tumor (ie individual patient) or tumor-type level.
Fig 3g. What does point color (blue/red/orange) mean ?
Page 11 line 305, are there 39,542 total CD4+ T cells or specifically Tconv ?

Reviewer #3

(Remarks to the Author)
This manuscript entitled "Molecular heterogeneity and clonal origin of CCR8+ effector regulatory T cells in human cancer" systematically delineates the heterogeneity of intratumoral regulatory T cells (Tregs) by integrating single-cell RNA sequencing and T cell receptor sequencing data from nine solid tumor types. The authors subdivided Tregs into four subtypes (quiescent, effector, unstable, and intermediate), and revealed that the clonally expanded CCR8+ effector Tregs show limited lineage relationships with other intratumoral Tregs but are clonally related to Tregs in adjacent normal tissues or draining lymph nodes and even share clones with some conventional CD4+ T cells. The authors aimed to provide insights for developing targeted Treg-depletion therapies.

Below are my questions and concerns to the authors:

1. CD74 is upregulated by CCR8+ICOS+ vs CCR8-ICOS- Treg, but it is only expressed by less than 5% CCR8+ICOS+ cells. Why CD74 upregulation matters in CCR8+ICOS+ Treg? Can it be useful as a therapy target in specific tumor type rather than a general target candidate?

2. In Fig1f, it's hard to claim Treg in colorectal cancer is more different while in other tumors they are similar. As in renal and iCCA, it seems Treg cells are less similar to other tumor types. Moreover, in Fig2e, the Treg subset fraction looks most different between colorectal and iCCA, but in Fig1f, it is colorectal vs renal showing lowest similarity.

3. The author claimed a subpopulation of unstable Treg because cytokine genes (IL2, TNF, IFNG, IL17A and TBX21) are expressed. Since no evidence showing this Treg subset tends to differentiate into Th1 or Th17, it's misleading to name it as unstable cells. Similarly, CD4 T cells with granzyme B and perforin expressing is just cytotoxic CD4 T, not "unstable" cells becoming cytotoxic CD8 T cells.

4. Flow cytometry analysis defined diverse effector Treg in lung and breast tumors. Is any of those diversity consistent with single cell data?

5. By integrating scTCR data, the author concluded tumor effector Treg cells are coming from TDLNs and NAT (ca. 14%), instead of other Treg clusters in the tumor (ca. 6%). This is reasonable because they migrate between tumor and LNs during tumor antigen-triggered immune response. But in fig2h, how should we think about the trajectory results showing four Treg subsets have complicated differentiation relationship?

6. Peripheral naïve CD4 T cells can differentiate into pTreg in certain conditions. However, analysis only based on scTCR data is not enough to conclude conventional CD4 T cells is the progenitor of tumor effector Treg. Tconv and Treg can

recognize the same tumor neoantigens and undergo clonal expansion. The authors have to rule out this probability to make their conclusions.

Version 1:

Reviewer comments:

Reviewer #1

(Remarks to the Author)
The authors have addressed all my comments/concerns
two minor comments for consideration.
1. Although I appreciate the performance of the suppression assay, I would expect a comment on the suppressive activity of unstable Treg cells which appeared to be similar to other Treg cell subsets
2. Since IFNg is expressed by unstable Treg cells could they belong to the previously reported Fragile Treg cells? (PMID: 31844326, PMID: 30068755. This may be included in the discussion.

Reviewer #2

(Remarks to the Author)
The authors have done an excellent job with revisions and the manuscript is much improved. My remaining comments are listed below.

Major comments :

From Fig 1d/page 6 lines 149-158, it appears CCR8 is not in the 88 gene signature ? This is a bit surprising given the literature on CCR8+ Tregs and the use of FACS sorted CCR8+ Tregs elsewhere in this study. Can the authors see why that is (eg is it not significantly differentially expressed at all ? Or in too few tumor types ?) At least discussing this is important

From the Methods section (page 22 line 674-675) it appears that the Treg specific subclustering (ie Fig 2a) only redid the clustering step, whereas it is generally considered best practice in “high resolution” analyses like this to recalculate highly variable genes, redo PCA, etc on the selected subset of cells. It is important to verify that this more thorough approach would not change important conclusions. Relatedly, I am confused by the two umaps in extended data figure 4 b. If only clustering has changed, why have the positions of the cells in the umap space also changed ?

Minor comments :

Fig 1f and Page 7 line 187. Pearson correlations are not intrinsically guaranteed to be unaffected by sample size differences and it would be useful to indicate, as they did in the previous round of referee responses, that the correlation patterns in Fig1f are not associated with the number of Treg cells / tumor type.

To help interpret Fig2i it would be helpful to have some visualization or table showing whether high/low for one Treg subpopulation is (anti-)correlated with high/low for another. Eg, are the results for Treg effector and Treg unstable “just” because if a tumor is high for one it is low for the other.

The color scale for Fig 3e is misleading. Since they have chosen to only show positive Z scores (justifiable since the point is to highlight TFs specifically active in a given cluster), they should use a single gradient, not something that goes from one color (here blue) to white to another color (here red).

Is tumor subtype available for the BC (luminal vs basal, or HR+ vs HER2+ vs TNBC) and NSCLC (LUAD vs LUSC) samples analyzed in Fig 4 ? If so, please include.

Reviewer #3

(Remarks to the Author)
The term “unstable Treg” should be avoided. An unstable cell could be interpreted in many ways; for instance, a cell undergoing apoptosis could be considered unstable in terms of survival. The authors provided transcriptional-level and flow-based evidence, but all of these could simply indicate that Treg cells are functionally heterogeneous, which is well known in the literature. More experimental data are needed to support the claim that Treg cells are on different differentiation trajectories.

Apart from this, I have no other questions.

Version 2:

Reviewer comments:

Reviewer #2

(Remarks to the Author)
The authors have addressed all of my concerns, I have no further suggestions to make.

Open Access This Peer Review File is licensed under a Creative Commons Attribution 4.0 International License, which permits use, sharing, adaptation, distribution and reproduction in any medium or format, as long as you give appropriate credit to the original author(s) and the source, provide a link to the Creative Commons license, and indicate if changes were made.
In cases where reviewers are anonymous, credit should be given to 'Anonymous Referee' and the source.
The images or other third party material in this Peer Review File are included in the article's Creative Commons license, unless indicated otherwise in a credit line to the material. If material is not included in the article's Creative Commons license and your intended use is not permitted by statutory regulation or exceeds the permitted use, you will need to obtain permission directly from the copyright holder.
To view a copy of this license, visit https://creativecommons.org/licenses/by/4.0/

REVIEWER COMMENTS

We thank all the Reviewers for the positive and constructive feedback on our manuscript. We addressed all possible concerns in the point-by-point below, by including new functional and computational analyses. We think that the manuscript has substantially improved and hope that it will be now acceptable for publication.

Reviewer #1 (Remarks to the Author):

In this manuscript by J. Swatler et al, the authors perform a comprehensive integration of published scRNAseq data from 9 tumor types in order to identify Treg cell specific signatures that will allow the precise targeting of intratumoral Treg cells, which will hold an enormous therapeutic benefit. Towards this they identified a core signature consisted of 88 genes to be highly expressed gy Treg cells in those tumors and they also describe 4 Treg cells subsets with distinct tissue distribution.
The concept is highly novel, since there is an urgent need to identify tumor-specific Treg cell signatures that will allow their ablation which will promote tumor regression but will also facilitate better responses during cancer immunotherapy. Nevertheless, my major concern in this manuscript, is that the authors do not take into account the tissue specific Treg cell signatures that are present in healthy situations. It is well accepted that Treg cells present tissue specific adaptations during homeostasis (doi.org/10.1016/j.it.2025.12.001), which I would expect to influence or shape the tumor microenvironment.

We thank the Review for positive perception of our manuscript and the constructive feedback on the data. We address all the concerns under specific questions below.

Specific comments.
1. How different healthy Treg cells (circulating or tissue resident) are, regarding the core signature, described in this study.
Response: We have addressed this by integrating 2 independent scRNA-sequencing datasets of Treg cells coming from "healthy" donors (brain-dead organ donors, not suffering from cancer) (PMID 36658238 , 35549404) into our atlas of Treg cells from 9 tumors. We compared enrichment of the core intratumoral 88 gene signature between Treg cells from 9 tumors (blood, NAT and tumor separately) and healthy Treg cells from blood, lymphoid organs (bone marrow, spleen, lymph nodes, thymus) and tissue (skin, lung, jejunum, intestine, fat) in **Extended Data Fig. 2b**. In general, while tissue Treg cells are enriched for expression 88 genes compared to blood, they have lower enrichment than intratumoral Treg cells, and comparable enrichment to Treg cells from normal adjacent tissues (NAT) of tumor patients. Therefore the 88 gene core signature enables preferential targeting of tumor Treg while sparing healthy counterparts. This aspect is emphasized in the Discussion.

2. What are the suppressive signatures expressed by the 4 Treg cells clusters, via which they may exert their function? How do they contribute to tumor development?
Response: We have checked the enrichment of the 88 gene core signature among 4 Treg clusters in tumors (showing blood as reference), as well as manually curated signature of genes related to suppressive function, as suggested by the Reviewer. We report this in **Extended Data Fig. 4c, d**, showing that all tumor Treg subsets have substantial enrichment of both signatures compared to blood, and the effector subset (C2) exhibits highest enrichment. Moreover, we validated this experimentally in a functional *in vitro* suppression assay by sorting the 4 Treg subsets from NSCLC tumors and report the data in **Fig. 2h**. All subsets inhibited Tconv proliferation, with the CCR8^{+} effector subset exhibiting superior suppressive activity and the CCR7^{+} subset showing the weakest. To dissect contribution to tumor development, we reanalyzed a dataset of intratumoral immune cells in NSCLC which also contained data on disease recurrence post-surgery (PMID 40147443) and correlated abundance of 4 clusters among the Treg population with recurrence free survival (RFS), reported in **Fig. 2i**. Consistently, abundance of *CCR8*^{high} effector Treg cells predicts lower RFS, while *KLRB1*^{high} unstable Treg cells are beneficial to higher RFS.

3. Considering the important role of Treg cells in the maintenance of the immune homeostasis but also on their influence in the development of immune-related adverse events, It is very important (similar to comment 1) to describe whether you also have expression of the core signature by naive Treg cells (circulating) and if this signature maybe altered in patients developing irAEs after immunotherapy.

Response: As mentioned in response to comment 1, we have checked enrichment of the 88 gene signature in circulating (blood) Treg cells, but also Treg cells early in development in the thymus (**Extended Data Fig. 2b**). As requested by the Reviewer we have further checked this in the context of immune checkpoint inhibitor-related Colitis (irColitis; PMID 38724705), the most common irAE post immunotherapy and report the data in **Extended Data Fig. 2c**. The signature in blood cells is consistently low, also during irColitis, but colon-resident Treg cells acquire degree of expression during irColitis, comparable to Treg cells in the normal adjacent tissue during colorectal cancer. This suggests acquisition of the 88 gene signature during inflammation, consistently with the role of Treg cells in inflammation resolution. The enrichment is still lower than in intratumoral Treg cells.

4. In a similar manner, it would be of great interest to analyze whether the presence of the core signature (or another) by Treg cells, may dictate responses to immune checkpoint inhibitors.
Response: The role of Treg cells in responses (and resistance) to immune checkpoint inhibitors has been described recently (PMID 37713507, PMID 37548431), where other groups have identified that presence of highly activated (GITR^{hi}OX-40^{hi}) Treg subsets correlated with lack of response to checkpoint inhibitors in lung tumors. Consistently with that, we checked the enrichment of our 88 gene signature in a well defined cohort of responder and non-responder patients, report results in **Extended Data Fig. 2d** and further describe in the Discussion. These data suggest that targeting genes of the core signature could have application in resistance to checkpoint inhibition. This is matter of further investigation in our lab in a second manuscript.

5. How do the Authors envision the targeting of the 88 core signature? In other words, in the clinic, what may be the advantages of the 88 core signature compared to targeting of CCR8+ Tregs, in a therapeutic setting?
Response: We further explain the rationale of the analysis in the paper Discussion. We explain that our analysis can serve as a resource to target new molecules overexpressed on Treg cells which of course would need further studies. We also propose technologies such as bispecific antibodies targeting two effector Treg molecules at the same time as a solution to increase specificity in the tumor setting.

Reviewer #2 (Remarks to the Author):

Tregs are an immunosuppressive subset of CD4+ T cells that play beneficial roles in normal physiology but are highly deleterious in cancer by blocking effective cancer immunity. Immunotherapies targeting Tregs can be efficacious but often induce dose-limiting toxicities linked to autoimmunity. In order to identify potential markers of tumor-specific Tregs and to better understand their ontogeny, this study focuses on a large cohort of scRNAseq of T cells infiltrating tumors and normal adjacent tissue across 9 tumor types assembled from the literature. The authors perform extensive flow cytometry profiling of an inhouse cohort of lung and breast cancer patients for validation and investigate a public scRNA/TCRseq dataset of TILs from NSCLC to study migration and differentiation at the TCR clone/lineage level.
The data appears to be of high quality and assembling this large cohort across different studies is a valuable and efficient approach. However, the analyses need to be extended significantly to make the conclusions robust and self-consistent and conclusions concerning differentiation pathways should be tested experimentally, eg with sorting and in vitro differentiation.

We thank the Reviewer for appreciation of our manuscript and the constructive suggestions on the data improvement. We have significantly extended bioinformatic analyses as requested, including new methods and datasets, as well as performed experimental differentiation assays on sorted Treg cells, to validate differentiation pathways. We address all the concerns under specific questions below.

Major comments
1. Figure 1b, this clustering and annotation is not fully convincing and I have concerns that more Treg cells might be in the dataset as subsets of clusters other than cluster 3. It is surprising that no cycling cluster is identified. There are 3 clusters annotated only as "undefined" and several others only labelled by a few marker genes as opposed to a formal cell type/cell state. The top filtered marker genes for cluster 10 in supplemental table 1 all look like myeloid genes. Could this be contamination ? Or T:myeloid doublets ?
Response: In response to the Reviewer's request, we have improved and explained several aspects of CD4$^+$ T cell clustering (**Fig. 1b**).

1) We have re-annotated the clusters based on expression of marker genes of T cell differentiation (new dot plot shown in **Extended Data Fig. 1a**), assessing memory status (naïve, central memory CM, effector memory EM) or functional specification (effector, Treg, Th17, cycling, etc.). Cluster 11 expresses *MKI67* and *TOP2A*, in addition to *CXCL13*, *PDCD1*, *HAVCR2*, thereby identifying cycling CD4$^+$ T cells with T follicular helper cell/exhausted T cell identity.
2) We checked lineage identity of all CD4$^+$ Tconv clusters, and report the data below as supporting information for the Reviewer. All clusters had high and comparable *CD3E* expression, confirming their T cell identity. On the other hand, none of the clusters had clear *CD14* or *CD68* expression, excluding contamination with myeloid cells, despite, as Reviewer noticed, some expression of myeloid genes in cluster 10, according to the gene table (**Supplementary Table 1**). For reference we show another dataset of intratumoral CD45$^+$ leukocytes (generated in house, unpublished), where myeloid clusters (such a C2, 3, 4, 12) have very different expression of *CD14* and *CD68*, but no *CD3E*. Possibly, increased expression of myeloid genes in C10 compared to other clusters is due to contamination with residual myeloid RNA or some kind of cell aggregates, that still passed the bioinformatic filtering that we applied.
3) Finally, regarding that clusters other than C3 can contain Treg cells, please also see response to the next concern by the Reviewer, where we found that the core signature of 88 genes was always upregulated with high significance in Treg cells (C3) when compared to all other clusters individually, suggesting little Treg "contamination" in Tconv clusters. A noticeable exception was C11 of cycling Tfh/exhausted Tconv. Seminal work of Sakaguchi group (PMID 19464196) has shown that some CD4$^+$ T cells can transiently express FOXP3 or CD25, but are not bona fide Treg cells. Therefore, if we attempted to find such cells in clusters other than C3, we would, firstly, lose the unsupervised nature of clustering and, secondly, potentially include cells that are not actually Treg, potentially present only in some tumors or patients, and introduce artifacts in downstream analyses. We stress this aspect also in the corrected manuscript.

a. *CD3E*, *CD14* and *CD68* expression in CD4 clusters from **Fig. 1b**.

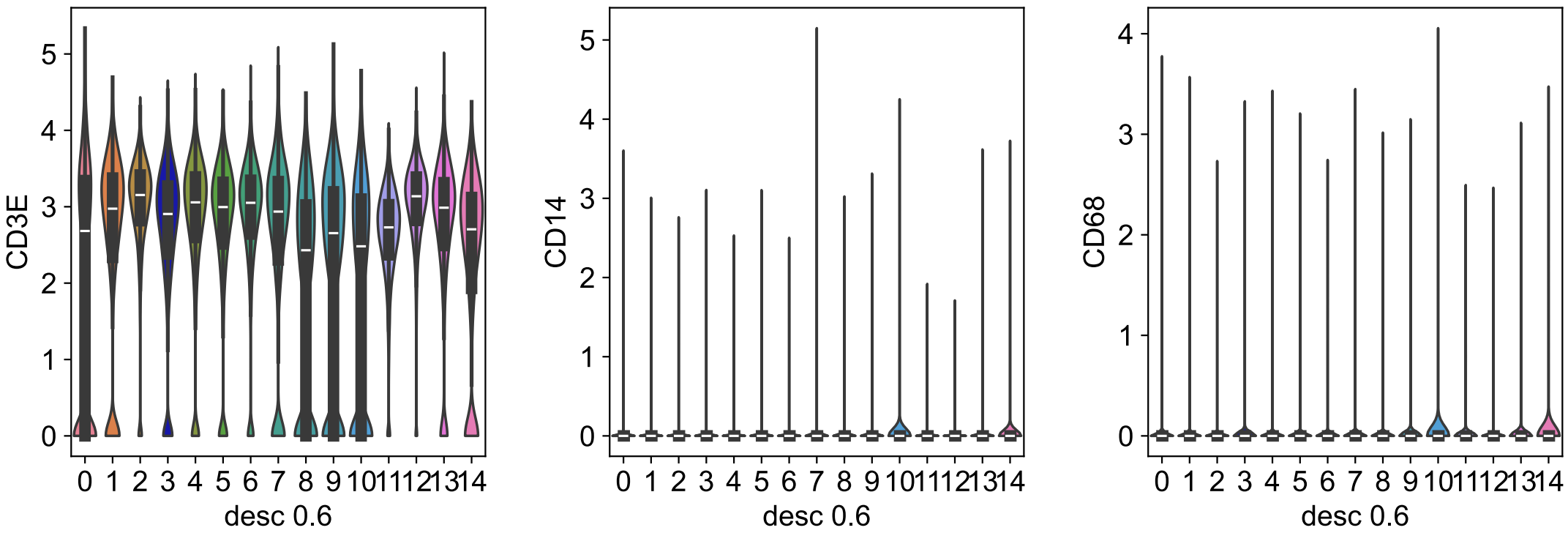

b. *CD3E*, *CD14* and *CD68* expression in CD45 intratumoral leukocyte clusters from a reference in-house dataset.

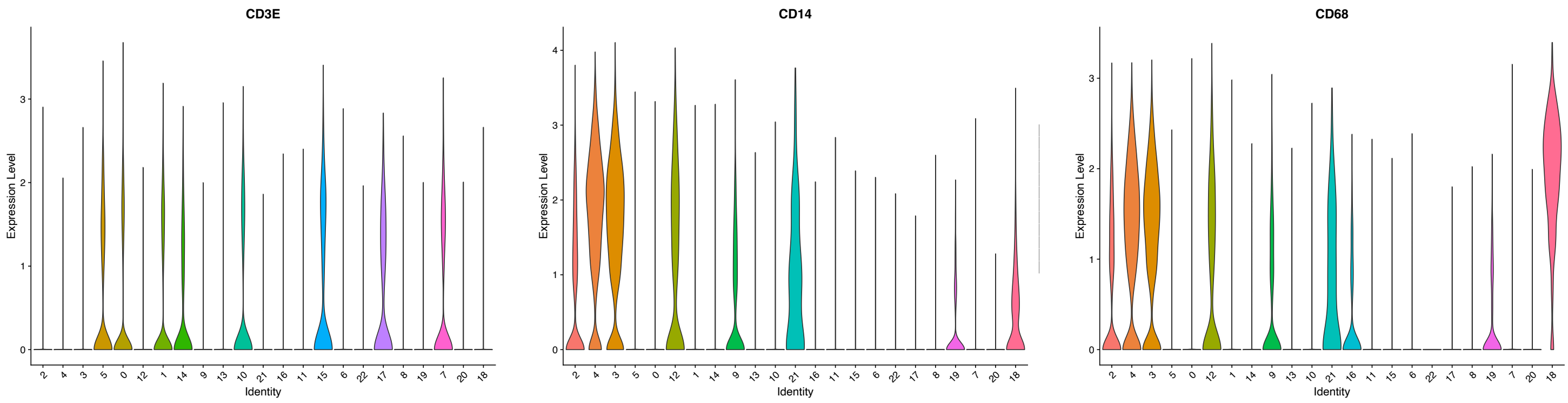

2. The definition of Treg marker genes (Fig 1d) should be complemented with all pairwise comparisons of Tregs vs each Tconv subcluster. The current approach of cluster 3 vs everything else can easily miss genes that are in fact highly expressed on minor Tconv subclusters.

Response: The initial approach we took was aimed at identifying molecules that could be used to target Treg cells while sparing the vast majority of the CD4$^+$ T cell pool. The approach proposed by the Reviewer indeed enables identification of even more specific or universal targets. We first checked the general enrichment of 88 gene signature in all CD4$^+$ T cell clusters, showing superior AUC score in Treg (C3), also computing statistic to clusters which have shown minor enrichment (C11, C12, C14) (**Extended Data Fig. 2a**), confirming specificity to C3. As suggested by the Reviewer, we also computed differential gene expression between C3 and each Tconv subcluster, shown in **Supplementary Table 4**. As discussed in the corrected manuscript, all 88 genes were significantly upregulated in every comparison, with notable exception of C11. As these cells are cycling/highly activated, some activation markers were upregulated in C11 vs C3. Nevertheless, the majority of 88 genes were still upregulated in C3 vs C11. We hope adding this data makes our resource more comprehensive and enables more critical and precise interpretation of the data.

3. Fig 1d, why visualize Z score instead of fold-change or log fold-change ? How exactly was z-score calculated ?

Response: In **Fig. 1d**, we chose to visualize z-scores rather than fold-change or log fold-change because the purpose of this panel was to summarize the relative strength and consistency of the differential transcriptional signal across cancer types, in a directly comparable way across many genes. Fold-change/log fold-change values can vary substantially in scale depending on baseline expression and, in our opinion, are less suitable for side-by-side visualization across heterogeneous genes. By contrast, z-scores place all genes on a common standardized scale, allowing the heatmap to emphasize whether a given gene shows a relatively stronger or weaker enrichment in tumor Treg cells across indications. Specifically, for each comparison, the value shown in the heatmap corresponds to the standardized differential-expression statistic (z-score) for each gene, with positive values indicating relative enrichment in tumor Treg cells compared with the reference population. Mean z-scores were generated with “scanpy.pp.scale” function (for gene expression standardization) and “sc.pl.matrixplot” (for visualization) function of scanpy workflow. The plotted value represents the mean z-score for each gene within each cancer type. The code and pipeline for

calculation of Z-scores and data visualization are included in GitHub code availability for this paper, under "https://github.com/luglilab/Treg-diversity-in-human-tumors-" (Part8_Fig1d_plot).

4. Fig 1f,g. Are patterns of similarity and difference between tumor types related to sample size per tumor type and/or tissue ?

Response: We appreciate this important comment, and acknowledge limitations of approaches used. In **Fig. 1f**, we computed Pearson correlation based on (average) gene expression matrix, therefore the data is unbiased by sample size. In **Fig. 1g**, we computed differential gene expression analysis in each tumor vs. everything else, so naturally this can be biased by sample sizes, as more cells generate more statistical power. However, we need to state that assembling datasets with exactly the same number of cells is virtually impossible. Also, we double-checked amount of single tumor Treg cells used for each tumor, as following: colorectal: 2406; lung: 1543; breast: 1282; esophagus: 741; ovarian: 661; endometrial: 265; iCCA: 151; renal: 149; HCC: 129. Hence, the number of tumor specific DEGs does not directly follow the pattern of cell number, as for example lung has fewest DEGs, but 2nd highest number of cells, while renal has 2nd highest amount of DEGs, while 8th number of cells. We transparently stated in the manuscript that **Fig. 1f** is unbiased approach, while **Fig. 1g** can be potentially skewed by sample size. In any case, to strengthen our conclusions on differences between tumors, we also provide new scRNA-seq data (**new Fig. 4h**) to confirm tumor-specific differences in Treg phenotypes previously observed by flow cytometry (also in response to Reviewer #3 point 4 below). While breast cancer Treg cells upregulate *HLADR* genes, NSCLC Tregs upregulate *CXCR6*, suggesting differential response to CXCL16 chemoattractant.

5. Pages 6/7, lines 174-175 hypothesizes that clustering of Breast and Ovarian Tregs is due to predominance of female patients. Can this be validated with female patients of other tumor types ? ChrX/Y gene expression should be usable to infer sex even if it's not in clinical annotations.

Response: We have only speculated about sex as a factor that influences Treg cell profile in breast and ovarian patients, as studying sex differences has never been the aim of this paper. We amended the text, stating that dissecting sex differences in Treg biology is a topic that would require more extensive studies, beyond the scope of the current study.

6. Figure 2h, STREAM analysis to confirm C0 as an intermediate/transitional state seems tenuous. Do other pseudotime/trajectory algorithms validate this ? Relatedly, what if C3 are considered as root (ie as induced/peripheral Tregs) ? The later TCR sharing analysis is also used to address the question of exTregs or induced Tregs but the conclusions are not fully clear to me. Sorting specific populations and ex vivo differentiation would be highly informative.

Response: To further confirm C0 as intermediate cells, we have performed trajectory analysis using a second algorithm, Slingshot, and include the data in a new **Fig. 3a**. We detected 2 differentiation trajectories, where quiescent cells (C1) turn into intermediate (C0) and then can bifurcate into either unstable (C3) or effector (C2) cells. Also pseudotime analysis shows the intermediate nature of C0. In all algorithms, we always chose C1 as starting point, as these cells express markers of quiescence/naïve cells (*TCF7, LEF1, SELL, CCR7, KLF2*) and thus are earliest in differentiation according to well established dogmas of T cell differentiation/memory. Instead, C3 are considered differentiated cells incapable to "revert" to quiescent/naïve state, thus poorly justifying the use of C3 as root. As requested, we validated the "quiescent>intermediate>effector" trajectory experimentally, by studying fate of sorted, healthy donor, CCR7^{hi} quiescent Treg cells in real time, by differentiation *ex vivo* following activation with anti-CD3/28 and in the presence of tumor conditioned medium (in part mimicking conditions of the tumor micronevironment; new **Fig. 3c, d Extended Data Fig. 6a, b**). Indeed, upon *ex vivo* culture, these cells lose quiescent identity, while gradually becoming CCR8^{hi} effector. A fraction of CCR7^{-}CCR8^{-} intermediate cells remains uncommitted, reflecting observations using Slingshot and STREAM trajectories in **Fig. 3a, b**.
Regarding the clonal overlap studies between effector tumor Treg and Tconv, we have amended the analyses (**Extended Data Fig. 9a-c**) by including another scRNA/TCRseq dataset and amended our conclusions. We refer only to clonal overlap, which could leave room to different interpretations, including that the process of Treg to Tconv switching could occur vice versa and its exact dynamics would need to be dissected using fate-mapping experiments in mice, beyond the scope of this study.

7. In general the TCR sharing analyses do not seem to be conditioned on the number of cells per cluster or sample, which can have a large effect when not accounted for.

Response: We acknowledge this doubt of the Reviewer and we have extended the data in the manuscript to show a better, more transparent and extensive analyses of TCR repertoires. As we have initially used scRNA/TCR-seq data of 3 patients, for each analysis we present upset plots for each patient separately, in **Extended Data Fig. 7b, 8a, 9a**. Specifically:

1) For overlap between 4 intratumoral subsets of Treg cells, data from individual patients confirm that effector Treg cells present predominantly unique TCR clonality and little overlap with other Treg clusters. Furthermore, we validate this in a second, larger cohort of 231 NSCLC patients (PMID: 40147443) in **Extended Data Fig. 7c**, confirming that effector Treg present unique TCR clonality. These results are also confirmed with TCR clonality analysis in bulk RNA sequencing data of 5 patients, present already at first submission (**Fig. 5e**).
2) For overlap between tumor effector Treg and Treg in tissues, the clonal overlap with the LN Treg has again been observed in all 3 patients, and overlap with NAT Treg in 2/3 patients. This is biased by small amount of cells obtained in patient MD01-004. For overlap with LN Treg cells, we discuss that similar data were obtained by the Piaggio group in a preprint (published as preprint, https://www.biorxiv.org/content/10.1101/2025.09.17.674622v1), hence confirming robustness of our observations.
3) For overlap between tumor effector Treg and Tconv $CD4^+$ T cells, we only observe overlap in 2/3 patients. Analysis of a second, larger cohort of 231 NSCLC patients (PMID: 40147443) revealed that clonal overlap in 76% of tumors (presented in new **Extended Data Fig. 9b, c**), confirming that Tconv-Treg relationships are detected in human tumors, although not in all patients. We amended our observations and discuss these new data in the manuscript, emphasizing that the Tconv-Treg relationship should be clarified in the future with more specific experiments.

Overall, we hope the more extensive presentation of the data and inclusion of new cohorts for studies of effector tumor Treg origins is now more comprehensive and robust.

8. Fig 4f. It is hard to interpret this figure. It seems like there is a lot of sharing (ie everything except the lightest gray), especially of more expanded clones. Not finding sharing for lowly expanded clones could easily be a power issue.
Response: We thank the Reviewer for this comment and observation. In this type of analysis of TCR overlap by TRUST4 algorithm, reconstructed from bulk RNA sequencing (not normalized per cell number), it is not possible to assess how many cells exactly expressed a certain clone. Therefore, while we are able to count the number of TCRs and number of overlapping TCRs (as in **Fig. 5e, Extended Data Fig. 7e**), the clonally expanded cells may most contribute to the total TCR reads and "dominate" the type of visualization we initially put in Fig. 4f (first submission). We acknowledge the limitation of the figure and decided to remove it from the manuscript. The remaining data from scTCR-sequencing analyses still provide insight on clonal expansion of effector tumor Treg cells.

9. Fig 5c. Complement this by Tconv vs Treg differential expression volcano plots for the shared TCRs to be sure that the Tconv label is accurate (ie that they aren't misclassified Tregs)
Response: We have performed this important control experiment by comparing differential gene expression between TCR-overlapping Tconv and TCR-overlapping tumor effector Treg, as suggested by the Reviewer. We report the data in **Extended Data Fig. 9d** volcano plot and **Supplementary Table 14**. Treg specific genes such as *FOXP3, IL2RA, BATF, CTLA4* etc. are significantly higher in effector Treg, confirming that overlapping Tconv are not misclassified Tregs.

Minor comments
10. Although Fig 1a is a schematic, it would still be useful to explain what genes/signatures are used to identify T cells amongst CD45+ leukocytes, CD4+ T cells amongst total T cells, and Tregs amongst CD4+ T cells.
Response : **Fig. 1a** is intended as a schematic overview, but we agree that it is important to clarify the strategy used to define the different T-cell populations. In the revised manuscript (methods section "Batch-effect correction and unsupervised clustering"), we now state more explicitly that cell identities were assigned using a hierarchical annotation approach based on CellTypist and canonical marker expression. Specifically, broad immune populations, including T cells among $CD45^+$ leukocytes, were first identified using the CellTypist Immune_All_High reference model, supported by expression of canonical pan-T-cell markers such as *CD3D*, *CD3E*, and *TRAC*. The T cell compartment was then further refined using the Immune_All_Low model to resolve finer T cell subsets. Within this framework, $CD4^+$ T cells were defined based on CellTypist annotation together with *CD4* expression, while Treg cells were identified among $CD4^+$ T cells by their CellTypist assignment and expression of the canonical regulatory T-cell program, including *FOXP3*, *IL2RA* and others (**Fig. 1b, Extended Data Fig. 1a, b**). We have now clarified this point in the revised figure legend and Methods section.

11. Page 5 bottom paragraph clarify if the analysis is at the tumor (ie individual patient) or tumor-type level.

Response: The analysis was performed on tumor-type level. We clarified this in the corrected manuscript.

12. Fig 3g. What does point color (blue/red/orange) mean ?
Response: It is the standard interface of FlowJo flow cytometry software and color enrichment scale of pseudocolor plots. Orange and red correspond to gradually higher cell density in a certain area of the plot. We clarify this in the Figure legend.

13. Page 11 line 305, are there 39,542 total CD4+ T cells or specifically Tconv ?
Response: It refers to total CD4+ T cells, including Treg cells. We clarified in the text that this number includes 9,451 Treg cells.

Reviewer #3 (Remarks to the Author):

This manuscript entitled "Molecular heterogeneity and clonal origin of CCR8+ effector regulatory T cells in human cancer" systematically delineates the heterogeneity of intratumoral regulatory T cells (Tregs) by integrating single-cell RNA sequencing and T cell receptor sequencing data from nine solid tumor types. The authors subdivided Tregs into four subtypes (quiescent, effector, unstable, and intermediate), and revealed that the clonally expanded CCR8+ effector Tregs show limited lineage relationships with other intratumoral Tregs but are clonally related to Tregs in adjacent normal tissues or draining lymph nodes and even share clones with some conventional CD4+ T cells. The authors aimed to provide insights for developing targeted Treg-depletion therapies.

Below are my questions and concerns to the authors:

1. CD74 is upregulated by CCR8+ICOS+ vs CCR8-ICOS- Treg, but it is only expressed by less than 5% CCR8+ICOS+ cells. Why CD74 upregulation matters in CCR8+ICOS+ Treg? Can it be useful as a therapy target in specific tumor type rather than a general target candidate?
Response: We document high RNA enrichment of *CD74* on tumor Treg cells (**Fig. 1d**), but lower cell surface expression (**Extended Data Fig 3a, b**). Our observations in **Fig. 1d** show that it is equally highly expressed across all studied tumors, making it a general target. We have amended our manuscript by sharing observations from a recent paper on this topic (PMID 38702311): while lowly expressed on the surface (and thus less available e.g. for targeting with an antibody), CD74 still has an important role in intratumoral Treg biology by regulating cell motility, actin cytoskeleton and organelle organization/cell ultrastructure.

2. In Fig1f, it's hard to claim Treg in colorectal cancer is more different while in other tumors they are similar. As in renal and iCCA, it seems Treg cells are less similar to other tumor types. Moreover, in Fig2e, the Treg subset fraction looks most different between colorectal and iCCA, but in Fig1f, it is colorectal vs renal showing lowest similarity.
Response: In **Fig. 1f**, we claim colorectal is most different based on hierarchical clustering analysis (left side of the graph), where colorectal is a single node, most separate from the others. Indeed, this is reflected in the analysis of differential gene expression in **Fig. 1g**, where colorectal has most unique upregulated genes. We underlined this in the corrected manuscript. The graph in **Fig. 2e** reports division into 4 subsets, and their abundance in the tumor tissue rather than differential gene expression as a whole, hence conveying different information than **Fig. 1f**. Nevertheless, also in this case, please note that colorectal cancer Treg cells show the highest frequency of *KLRB1*^{high} unstable Tregs, and for this reason belonging to a separate node compared to other tumor-infiltrating Tregs. These aspects have been clarified in the manuscript.

3. The author claimed a subpopulation of unstable Treg because cytokine genes (IL2, TNF, IFNG, IL17A and TBX21) are expressed. Since no evidence showing this Treg subset tends to differentiate into Th1 or Th17, it's misleading to name it as unstable cells. Similarly, CD4 T cells with granzyme B and perforin expressing is just cytotoxic CD4 T, not "unstable" cells becoming cytotoxic CD8 T cells.
Response: We have performed additional functional experiments to assess the identity of these cells. Firstly, by demonstrating suppressive function in a standard suppression assay (new data in **Fig. 2h**), we confirm their functional Treg identity. However, at the same time, intracellular cytokine staining (IL-2, IFN-γ, TNF) on sorted 4 subsets of NSCLC Treg cells following PMA/ionomycin stimulation (new data in **Extended Data Fig. 5c**) implicates acquisition of Thelper properties by the unstable cells, especially when compared to the effector subset. Furthermore, we observed that these cells corelate with beneficial survival in lung cancer in terms of disease recurrence following surgery and neoadjuvant chemotherapy/anti-PD-1 (**Fig. 2i**). Altogether, with transcriptomic data and flow cytometry data on low FOXP3 expression (**Extended Data Fig. 4g**), our data suggest that these cells are reminiscent of Treg instability/fragility, described predominantly in autoimmune disease (PMID 29925983, 40494998, 23355538), but also recently in successful immunotherapy outcomes in mice (PMID 41932343, 28552348, 33588426). Hence, we named them as "unstable". However, if the Reviewer and Editor further believe this name is unsuitable, we will be happy to amend our manuscript.

4. Flow cytometry analysis defined diverse effector Treg in lung and breast tumors. Is any of those diversity consistent with single cell data?
Response: We provide new scRNA-seq data in **Fig. 4h** that validates differences between lung and breast tumors observed by flow cytometry (samples from **Fig. 2a**). We show that effector Treg cells

(cluster 2) from breast and NSCLC tumors distribute differently in the UMAP space (similar to observations from flow cytometry, **Fig. 4e**). By flow cytometry we observed higher abundance of HLA-DR^{+} (cluster 1, 4) and lower abundance of $Helios^{-}$ (cluster 8) and $CXCR6^{+}$ (cluster 6) effector Treg clusters in breast tumors when compared to NSCLC. Consistently, gene expression of *HLA-DRB1, HLA-DRB5* and *IKZF2* (encoding Helios) is higher in BC, while *CXCR6* is higher in NSCLC (full list of differential genes included in **Supplementary Table 9**). Therefore, our observations are consistent between different patient cohorts and technologies.

5. By integrating scTCR data, the author concluded tumor effector Treg cells are coming from TDLNs and NAT (ca. 14%), instead of other Treg clusters in the tumor (ca. 6%). This is reasonable because they migrate between tumor and LNs during tumor antigen-triggered immune response. But in fig2h, how should we think about the trajectory results showing four Treg subsets have complicated differentiation relationship?
Response: We have performed another trajectory analysis of genomic data (for all tissues together), using Slingshot algorithm, to provide a simpler, clearer model for differentiation relationships of 4 Treg subsets that we describe, and included the data in a new **Fig. 3a**. We observe 2 trajectories of differentiation, including one from quiescent, through intermediate, towards effector cells (C1>C0>C2). This also enables clearer understanding of relationships between lymph node and tumor Treg cells, as inquired by the Reviewer (**Fig. 5h**) which we now explain in the manuscript. LN Tregs that are non-clonally related to tumor Tregs are characterized by the expression of quiescent genes (*CCR7*, *IL7R*, *SELL*, *LEF1*), whilst tumor Tregs are characterized by the expression of effector genes (*CCR8*, *TNFRSF9*, *BATF* etc.). The LN Tregs that exhibit clonal overlap with those in tumors exhibit intermediate expression of these genes, suggesting loss of quiescence properties, but not full commitment to effectors. With these data, we conclude that upon maturation from LN to the tumor, Treg follow the trajectory described in **Fig. 3a**, with additional layer of migration between tissues.

6. Peripheral naïve CD4 T cells can differentiate into pTreg in certain conditions. However, analysis only based on scTCR data is not enough to conclude conventional CD4 T cells is the progenitor of tumor effector Treg. Tconv and Treg can recognize the same tumor neoantigens and undergo clonal expansion. The authors have to rule out this probability to make their conclusions.
Response: We thank the Reviewer for this crucial suggestion. We toned down our message and refer only to clonal overlap, which could leave room to different interpretations. One of these is indeed that some Treg and Tconv cells with the same TCR can exist and recognize the same tumor neoantigens (PMID 31924652, 35508657). We further amended the paper Discussion explaing that eTreg:Tconv conversion could occur vice versa or that these cells could have a common progenitor in the tumor. The exact dynamics would need to be dissected using fate-mapping experiments in mice, beyond the scope of this study.

REVIEWER COMMENTS

We thank the Reviewers for the positive feedback on our first manuscript revision and further constructive comments. We addressed all concerns in the point-by-point below, by including new computational analyses, amending the methods and discussion. We think that the manuscript has improved and hope that it will be now acceptable for publication.

Reviewer #1 (Remarks to the Author):

The authors have addressed all my comments/concerns
two minor comments for consideration.

We are happy to have satisfied all the comments and concerns of the Reviewer. We have amended the paper accordingly, to address the remaining comments below.

1. Although I appreciate the performance of the suppression assay, I would expect a comment on the suppressive activity of unstable Treg cells which appeared to be similar to other Treg cell subsets

Response: We agree that this result may be surprising. We also reported the data that the unstable (now CD161$^+$) Treg cells maintain high expression of effector molecules TIGIT, CD39, OX40 and GITR in tumors (**Fig. 2f, Extended Data Fig. 4g**), indicating that they may still suppress comparably to quiescent or intermediate cells (but less than effectors, as shown). As suggested, we have discussed this aspect, indicating that functionality of these cells may be different in NSCLC when compared to literature reports for CRC (such as PMID 27111280, also referred in our manuscript) or that the "gold standard" suppression assay may not capture all their "proinflammatory" features, e.g. modulation of myeloid cells by IFNγ. The predictive value for recurrence-free survival (**Fig. 2i**) further indicates that these CD161$^+$ Treg cells may contribute to anti-tumor, rather than pro-tumoral responses.

2. Since IFNg is expressed by unstable Treg cells could they belong to the previously reported Fragile Treg cells? (PMID: 31844326, PMID: 30068755. This may be included in the discussion.

Response: We agree with this suggestion and further elaborate on this in the paper Discussion, citing the papers suggested by the Reviewer.

Reviewer #2 (Remarks to the Author):

The authors have done an excellent job with revisions and the manuscript is much improved. My remaining comments are listed below.

We appreciate the positive feedback on our revision work and manuscript improvements. We address the remaining concerns below and amended the manuscript accordingly.

Major comments :

1. From Fig 1d/page 6 lines 149-158, it appears CCR8 is not in the 88 gene signature ? This is a bit surprising given the literature on CCR8+ Tregs and the use of FACS sorted CCR8+ Tregs elsewhere in this study. Can the authors see why that is (eg is it not significantly differentially expressed at all ? Or in too few tumor types ?) At least discussing this is important

Response: We thank the Reviewer for pointing this out. We added data on *CCR8* expression across 9 tumor types in **Extended Data Fig. 1d**, showing z-score (as for other genes in **Fig. 1d**), as well as report score, log fold-change (LFC) and adjusted p-value from the original data in **Supplementary Table 2**. *CCR8* is significantly upregulated in 7 out of 9 tumors. In HCC and iCCA *CCR8* is also upregulated, but the data does not reach statistical significance, explaining why *CCR8* does not meet criteria of 88 gene signature (at least 8 out of the 9 tumors analyzed). We clarified this in the manuscript.

2. From the Methods section (page 22 line 674-675) it appears that the Treg specific subclustering (ie Fig 2a) only redid the clustering step, whereas it is generally considered best practice in "high resolution" analyses like this to recalculate highly variable genes, redo PCA, etc on the selected subset of cells. It is important to verify that this more thorough approach would not change important

conclusions. Relatedly, I am confused by the two umaps in extended data figure 4 b. If only clustering has changed, why have the positions of the cells in the umap space also changed ?
Response: We thank the Reviewer for raising this important point. We clarified this aspect in the revised Methods section. For the Treg-specific subclustering analysis, we did not simply repeat the clustering step on a fixed low-dimensional embedding. Rather, after isolating the Treg cells, we repeated the full analysis workflow on this selected cell subset, including recalculation of highly variable genes, dimensionality reduction, and clustering, using DESC (PMID 32393754) at a resolution of 0.5. Therefore, the analysis in the paper has been performed as suggested by the Reviewer. This is now better clarified in the Methods. Regarding **Extended Data Fig. 4b**, the two panels shown in this figure correspond to DESC analyses performed at different resolution parameters, rather than to alternative cluster labels projected onto the same precomputed UMAP. Because DESC jointly learns a latent representation during clustering, changing the resolution parameter can affect both the final cluster assignments and the latent space used for UMAP visualization. As a result, the relative positions of cells in the UMAP can differ between the desc_0.5 and desc_0.7 analyses, opposite to what happens when applying e.g. Seurat. We have clarified this point in the revised Methods.

Minor comments :
1. Fig 1f and Page 7 line 187. Pearson correlations are not intrinsically guaranteed to be unaffected by sample size differences and it would be useful to indicate, as they did in the previous round of referee responses, that the correlation patterns in Fig1f are not associated with the number of Treg cells / tumor type.
Response: We thank the Reviewer for this helpful comment. We agree that Pearson correlation values are not intrinsically independent of sample size and may, in principle, be influenced by differences in the number of Treg cells available for each tumor type. In case of **Fig. 1f** we have used gene expression matrix but, as requested, also verified number of cells used for the analysis (as previously): colorectal: 2406; lung: 1543; breast: 1282; esophagus: 741; ovarian: 661; endometrial: 265; iCCA: 151; renal: 149; HCC: 129. For example, despite low cell numbers, the differences between renal and colorectal cancer Treg are well visible, but not between renal and NSCLC which are very similar. We found no evidence that tumor types with higher or lower Treg cell numbers showed systematically different correlation profiles than tumor types represented by fewer Treg cells, indicating that the observed similarities across tumor types were not driven by differences in sampling depth. Thus, the structure observed in **Fig. 1f** is unlikely to be driven by differences in Treg cell abundance or sampling depth across tumor types, and we state this in the manuscript.

2. To help interpret Fig2i it would be helpful to have some visualization or table showing whether high/low for one Treg subpopulation is (anti-)correlated with high/low for another. Eg, are the results for Treg effector and Treg unstable "just" because if a tumor is high for one it is low for the other.
Response: We thank the Reviewer for this suggestion. We have plotted abundance (as % of total Treg cells) of each subset versus another and show the new data in **Extended Data Fig. 5d**. We also describe in the text that effector subset is significantly anti-correlated with all the other ones. As intermediate and quiescent subset abundances do not significantly predict RFS (**Fig. 2i**), it means that this anti-correlation does not necessarily predict relevance for survival, although, as the Reviewer pointed out, it is a valid expectation for the unstable ($CD161^+$) subset.

3. The color scale for Fig 3e is misleading. Since they have chosen to only show positive Z scores (justifiable since the point is to highlight TFs specifically active in a given cluster), they should use a single gradient, not something that goes from one color (here blue) to white to another color (here red).
Response: We corrected the graph in **Fig. 3e**, using only a gradient of red, to show positive Z-scores.

4. Is tumor subtype available for the BC (luminal vs basal, or HR+ vs HER2+ vs TNBC) and NSCLC (LUAD vs LUSC) samples analyzed in Fig 4 ? If so, please include.
Response: We indicated these information for most patients (though it was not always available) in the original source data file attached to the manuscript and report this availability in the Methods section. If available, we report if NSCLC patients were LUAD, and for BC we report if patients were HR+ luminal A/B or TNBC subtype, as well as HER2 status, if available.

Reviewer #3 (Remarks to the Author):

The term “unstable Treg” should be avoided. An unstable cell could be interpreted in many ways; for instance, a cell undergoing apoptosis could be considered unstable in terms of survival. The authors provided transcriptional-level and flow-based evidence, but all of these could simply indicate that Treg cells are functionally heterogeneous, which is well known in the literature. More experimental data are needed to support the claim that Treg cells are on different differentiation trajectories.
Response: We appreciate these advice and we therefore removed the term “unstable” as a name for this Treg cell subset. We refer to these cells based on their identity/phenotype as “CD161$^+$” (or “CD161$^+$ (*KLRB1*$^+$)” when we refer to genomic data and *KLRB1* transcript, encoding CD161). We only discuss the potential unstable/fragile state (in terms of function) of these cells in the light of existing literature, as also suggested by Reviewer 1. We also amended the Discussion that further studies are required on differentiation trajectory of these cells, especially in terms of signals inducing this subset and its fate.

Apart from this, I have no other questions.
Response: We are happy that we were able to address all the other concerns and comments.

REVIEWER COMMENTS

Reviewer #2 (Remarks to the Author):

The authors have addressed all of my concerns, I have no further suggestions to make.

Response: We are happy to have satisfied all the comments and concerns of the Reviewer.
