# Supplementary material for: Molecular heterogeneity and clonal origin of CCR8+ effector regulatory T cells in human cancer
Source: Nat Commun. 2026 Aug 21;17:9994. doi: 10.1038/s41467-026-76670-6 (PMC13590533; doi:10.1038/s41467-026-76670-6)
Supplement: Supplementary file 3 [file 41467_2026_76670_MOESM1_ESM.pdf]

SUPPLEMENTARY INFORMATION FOR:

Molecular heterogeneity and clonal origin of CCR8^{+} effector regulatory T cells in human cancer

by Julian Swatler[1,*,#], Simone Puccio[1,2,*], Emanuele Voulaz[1,3], Giuseppe Marulli[1,3], Matthew Wheeler[4], Sharmila Sambanthamoorthy[4], Eloise Scamardella[1], Simona Feno[1], Sabina Testa[1], Chiara Camisaschi[1], Valentina Errico[1], Matteo Simonelli[1,3], Agnese Losurdo[1], Emilia Maria Cristina Mazza[1] and Enrico Lugli[1,#]

1 IRCCS Humanitas Research Hospital, Rozzano, Milan, Italy
2 Institute of Genetic and Biomedical Research, UoS Milan, National Research Council, Rozzano, Milan, Italy
3 Department of Biomedical Sciences, Humanitas University, Pieve Emanuele, Milan, Italy
4 Discovery Biology, Bristol Myers Squibb Company, Redwood City, California, United States

*These authors contributed equally

#Corresponding authors: Enrico Lugli, Julian Swatler, Laboratory of Translational Immunology, IRCCS Humanitas Research Hospital, Via Manzoni 56, Rozzano (MI), Italy, Phone: +39 02 8224 5143/+39 02 8224 5161. E-mail: enrico.lugli@humanitasresearch.it; julian.swatler@humanitasresearch.it

SUPPLEMENTARY FIGURE 1. Characteristics and abundance of CD4+ T cell subsets across 9 tumors.

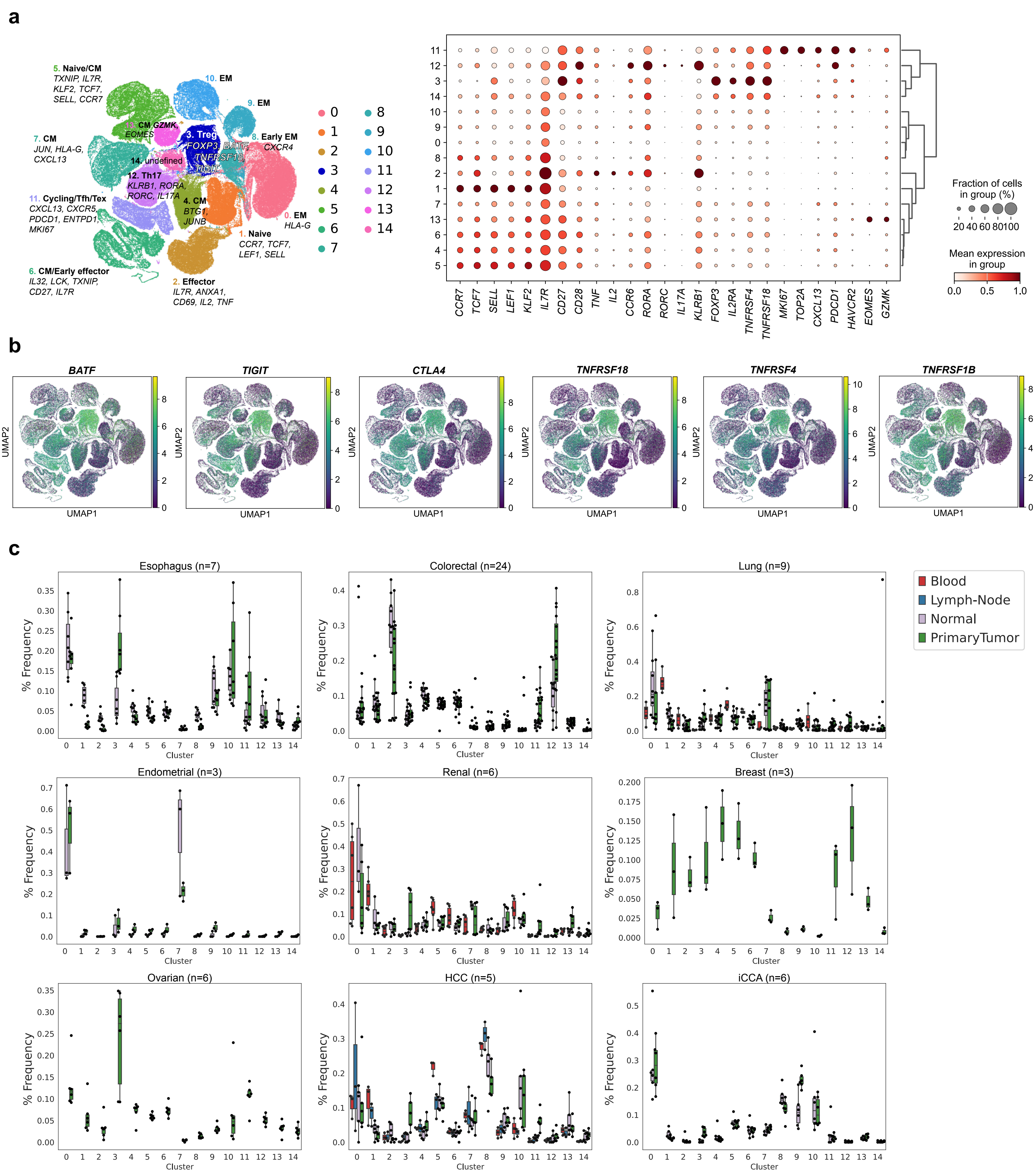

d

	Score	LFC	Adjusted p-value
Ovarian	16.94	4.85	3.46E-62
Breast	16.11	4.91	7.59E-57
Colorectal	14.37	5.44	1.09E-45
Lung	20.02	4.55	2.54E-87
HCC	1.53	25.74	0.71
Esophagus	12.12	4.23	8.03E-32
iCCA	1.89	4.79	0.62
Endometrial	4.1	6.41	0.001
Renal	15.97	6.67	1.01E-54

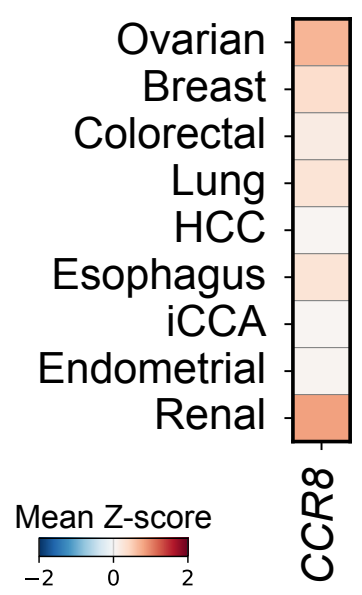

SUPPLEMENTARY FIGURE 1. Characteristics and abundance of $CD4^+$ T cell subsets across 9 tumors.

a. Right: Dot plot showing expression level and frequency of expressing cells for selected genes, in 15 clusters of $CD4^+$ T cells from integrated single-cell datasets of 9 solid tumors (as in Fig. 1b, shown on the left for reference).
b. UMAP plots demonstrating *BATF, TIGIT, CTLA4, TNFRSF1B* (encoding TNFR2), *TNFRSF4* (encoding OX40) *and TNFRSF18* (encoding GITR) expression in $CD4^+$ T cell clusters (as in Fig. 1b).
c. Frequency of $CD4^+$ T cell clusters identified in main text Fig. 1b, according to tumor type and tissue. The number of patients per tumor type is indicated. Bars indicate the interquartile range, lines the standard deviation. Single data points represent samples from separate patients.
d. Heatmap showing relative *CCR8* gene expression (mean Z-score) of tumor Treg versus tumor Tconv cells. Score, log fold change (LFC) and adjusted p-value (Two-sided t-test_overstim_var, Bonferroni correction) are reported on the right for the analysis in each tumor type (as in Supplementary Data 2). For (a-d): n=69 patients including n=7 esophagus, n=24 colorectal, n=9 lung, n=3 endometrial, n=6 renal, n=3 breast, n=6 ovarian, n=5 HCC, n=6 iCCA.

For (a, c, d), source data are provided as a Source Data file.

SUPPLEMENTARY FIGURE 2. 88 gene signature of Treg across different conditions.

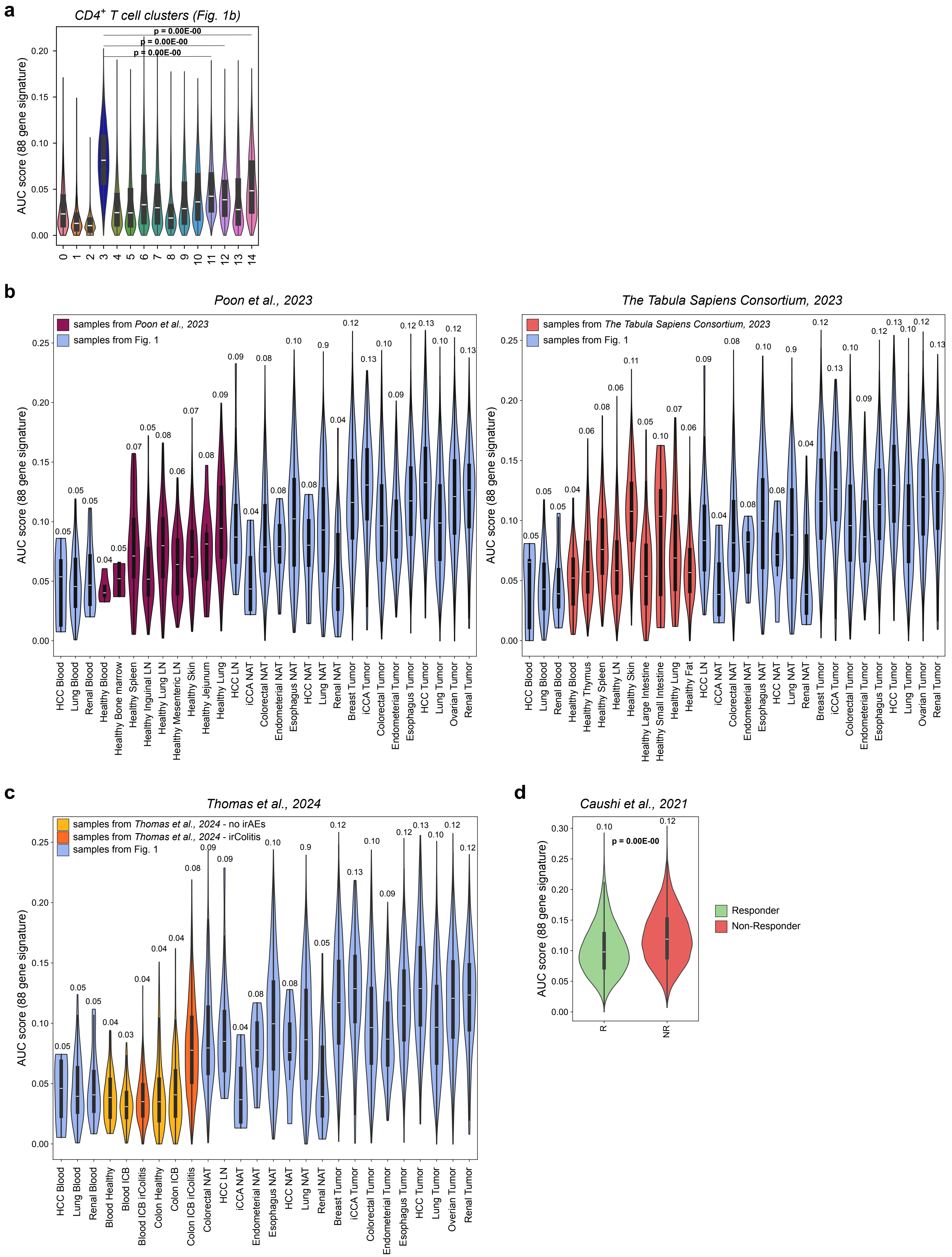

SUPPLEMENTARY FIGURE 2. 88 gene signature of Treg across different conditions.

a. Enrichment (AUC score) of 88 Treg cell transcriptional signature in 15 clusters of $CD4^+$ T cells from integrated single-cell datasets of 9 solid tumors (as in Fig. 1b). Mean values are indicated with white line. One-sided Mann-Whitney test. P-values of the enrichment between C3 (Treg) and C11, C12, C14 are indicated (n=69 patients including n=7 esophagus, n=24 colorectal, n=9 lung, n=3 endometrial, n=6 renal, n=3 breast, n=6 ovarian, n=5 HCC, n=6 iCCA).
b. Enrichment (AUC score) of 88 Treg cell transcriptional signature in samples of Treg from blood, NAT and tumor from Fig. 1 (in blue, n=69 patients including n=7 esophagus [n=7 NAT, tumor], n=24 colorectal [n=10 NAT, n=16 tumor], n=9 lung [n=4 blood, n=8 NAT, n=9 tumor], n=3 endometrial [n=3 NAT, tumor], n=6 renal [n=6 blood, n=3 NAT, n=6 tumor], n=3 breast [n=3 tumor], n=6 ovarian [n=6 tumor], n=5 HCC [n=3 blood, n=3 LN, n=5 NAT, n=5 tumor], n=6 iCCA [n=6 NAT, tumor]) and Treg of integrated healthy tissue samples from *Poon et al.*[1] (in burgundy, left, n=2 donors [n=1 blood, BM, n=2 spleen, inguinal, lung, mesenteric LN, skin, jejunum, lung]) or from *The Tabula Sapiens Consortium*[2] (in red, right, n=6 donors [n=4 blood, n=2 thymus, skin, small, large intestine, fat, n=3 spleen, LN, lung]). Mean values are indicated with white line and reported above violins.
c. Enrichment (AUC score) of 88 Treg cell transcriptional signature in samples of Treg from blood, NAT and tumor samples from Fig. 1 (in blue, n=69 patients as in (b)) and Treg of integrated blood and colon samples from *Thomas et al.*[3] (in orange, irColitis in dark orange, n=8 healthy donors [n=8 colon, n=3 blood], n=6 ICB [n=6 colon, n=5 blood], n=13 irColitis [n=13 colon, n=12 blood]). Mean values are indicated with white line and reported above violins. ICB: immune checkpoint blockade; irColitis: immune checkpoint inhibitor induced colitis.
d. Enrichment (AUC score) of 88 Treg cell transcriptional signature in samples of intratumoral Treg from ICB responder (R, in green) and non-responder (NR, in red) patients from *Caushi et al.*[4] (n=6 responder, 9 non-responder patients with NSCLC). Mean values are indicated with white line and reported above violins. One-sided Mann-Whitney test. P-value is indicated.

SUPPLEMENTARY FIGURE 3. Validation of Treg cells core signature genes by flow cytometry.

a

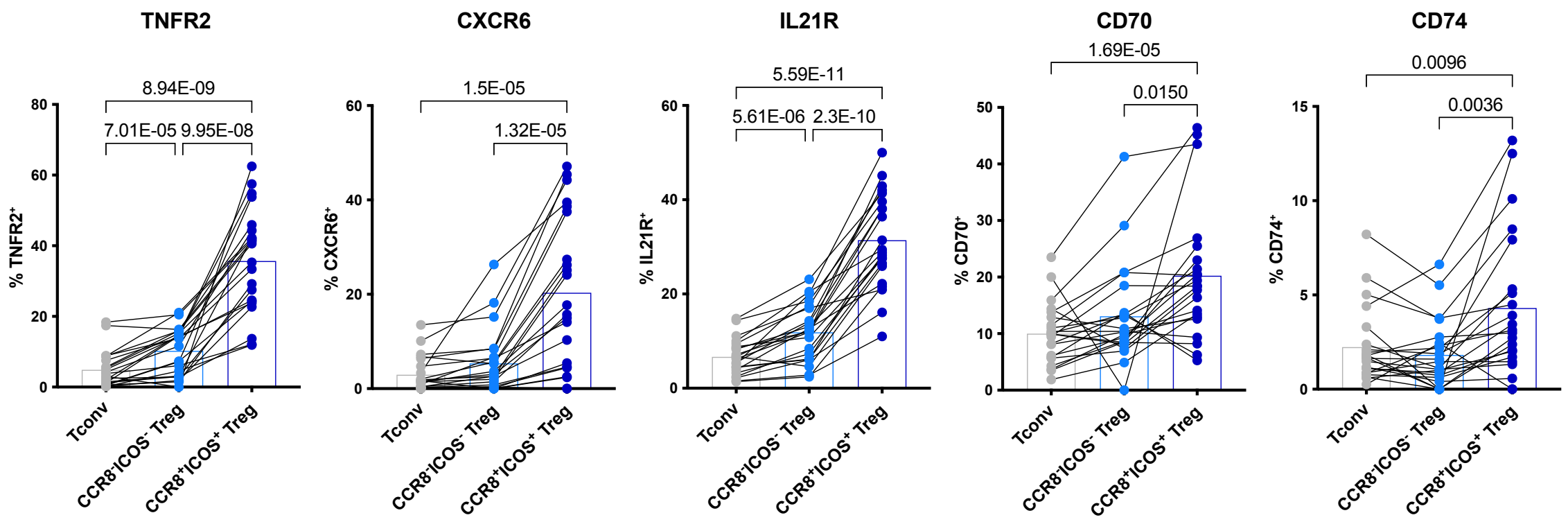

b

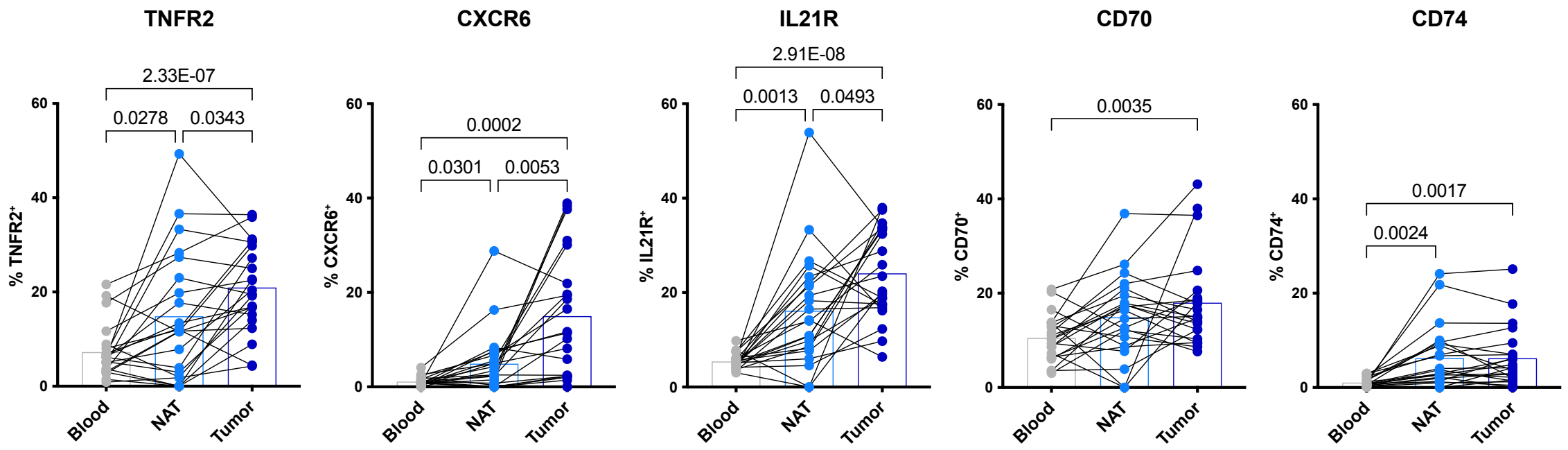

c

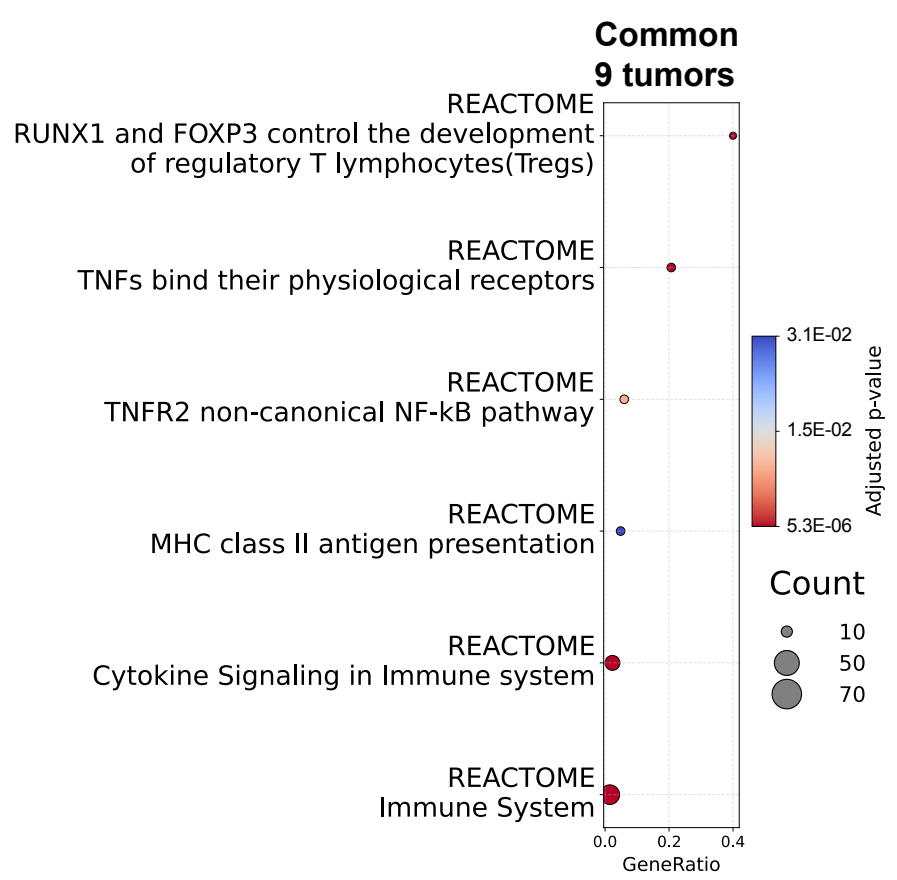

SUPPLEMENTARY FIGURE 3. Validation of Treg cells core signature genes by flow cytometry.

a. Analysis of TNFR2, CXCR6, IL21R, CD70, CD74 on subsets of intratumoral $CD4^+$ Tconv, and $CCR8^-ICOS^-$ and $CCR8^+ICOS^+$ Treg cells, by flow cytometry. For each marker, the summary of quantification, as percentage of positive cells, from 10 patients with BC and 12 with NSCLC is shown. One-way ANOVA with Tukey's post-test. Numbers on graphs indicate p-value.
b. Analysis of TNFR2, CXCR6, IL21R, CD70, CD74 on Treg cells from blood, NAT and tumor, by flow cytometry. For each marker, the summary of quantification, as percentage of positive cells, from 10 patients with BC and 12 with NSCLC is shown. One-way ANOVA with Tukey's post-test. Numbers on graphs indicate p-value.
c. Gene set enrichment analysis of common 88 Treg upregulated genes, as identified in main text Fig. 1c-d (n=69 patients including n=7 esophagus, n=24 colorectal, n=9 lung, n=3 endometrial, n=6 renal, n=3 breast, n=6 ovarian, n=5 HCC, n=6 iCCA). Processes from REACTOME collection are indicated. One-sided hypergeometric test, g:SCS correction.

For (a-c), source data are provided as a Source Data file.

SUPPLEMENTARY FIGURE 4. Identification of 4 subsets of regulatory T cells in tumors and tissues.

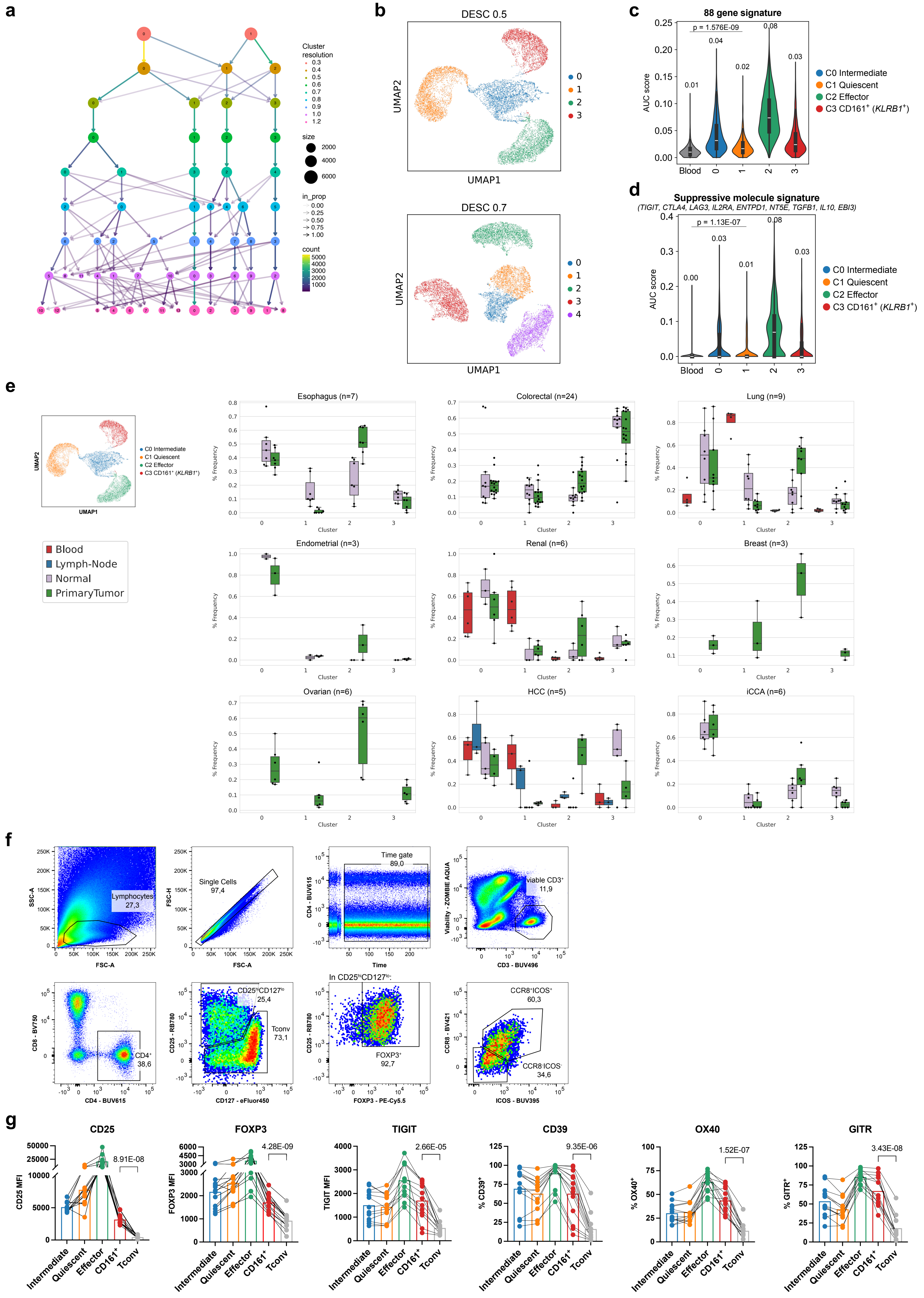

SUPPLEMENTARY FIGURE 4. Identification of 4 subsets of regulatory T cells in tumors and tissues.

a. Clustree plot of Treg cells clustering using DESC, to assess cluster stability at different clustering resolutions from 0.3 to 1.2.
b. UMAP visualization of 12,418 regulatory T cells, comparing cluster distribution at different DESC clustering resolutions of 0.5 (leading to identification of 4 clusters) and 0.7 (leading to identification of 5 clusters).
c. Enrichment (AUC score) of 88 Treg cell transcriptional signature in blood Treg cells and in 4 clusters of tumor Treg cells from Fig. 2a. Mean values are indicated with white line and reported above violins. One-sided Mann-Whitney test. P-value of the enrichment between C1 (quiescent tumor Treg) and blood Treg is indicated.
d. Enrichment (AUC score) of suppressive activity genes signature (indicated in header) in blood Treg cells and in 4 clusters of tumor Treg cells from Fig. 2a. Mean values are indicated with white line and reported above violins. One-sided Mann-Whitney test. P-value of the enrichment between C1 (quiescent tumor Treg) and blood Treg is indicated.
e. Frequency of Treg cells clusters identified in main text Fig. 2a, according to tumor type and tissue. The number of patients per tumor type is indicated. Bars indicate the interquartile range, lines the standard deviation. Single data points represent samples from separate patients. For (a-e): n=69 patients including n=7 esophagus, n=24 colorectal, n=9 lung, n=3 endometrial, n=6 renal, n=3 breast, n=6 ovarian, n=5 HCC, n=6 iCCA.
f. Representative flow cytometry gating strategy, on a selected tumor sample from a patient with BC. T cells were first identified based on physical parameters, dead cell exclusion and positive CD3 expression. $CD4^{+}CD8^{-}$ T cells were identified, followed by identification of $CD25^{hi}CD127^{lo}FOXP3^{+}$ Treg cells and $CCR8^{+}ICOS^{+}$ effector Treg cells.
g. Expression of selected phenotypic markers (either as mean fluorescence intensity [MFI] or percentage [%] of expressing cells) in 4 subsets of Treg cells and conventional T cells in tumor tissue, from 4 patients with BC and 9 with NSCLC. Two-tailed paired t-test to compare $CD161^{+}$ cells and Tconv was performed. Numbers on graphs indicate p-value.

For (e, g), source data are provided as a Source Data file.

SUPPLEMENTARY FIGURE 5. Characterization of 4 subsets of regulatory T cells in tumors and tissues.

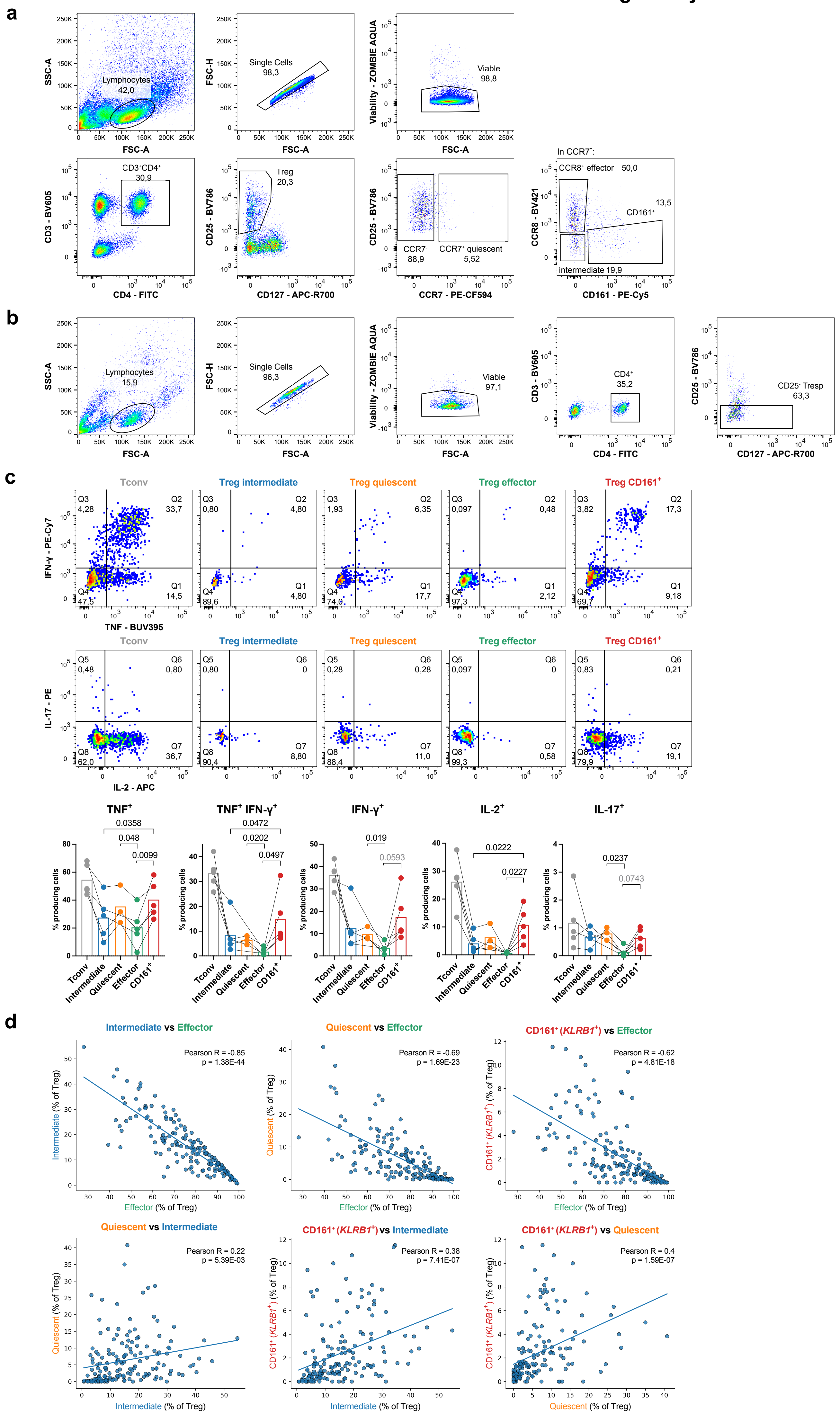

SUPPLEMENTARY FIGURE 5. Characterization of 4 subsets of regulatory T cells in tumors and tissues.

a. Representative flow cytometry sorting gating strategy, on a selected tumor sample from a patient with NSCLC, to obtain Treg for *in vitro* suppression assay and intracellular cytokine analysis. $CD4^+$ T cells were first identified based on physical parameters, dead cell exclusion and positive CD3 and CD4 expression, followed by identification of $CD25^{hi}CD127^{lo}$ Treg cells and 4 Treg subsets. Quiescent cells were first identified as $CCR7^+$, and then among $CCR7^-$ cells other subsets were identified. $CCR8^+$ cells were considered effector, $CD161^+$ and $CCR8^-CD161^-$ intermediate (as in Fig. 2f).
b. Representative flow cytometry sorting gating strategy, on a selected blood sample from a patient with NSCLC, to obtain responder T cells (Tresp) for *in vitro* suppression assay. $CD4^+$ T cells were first identified based on physical parameters, dead cell exclusion and positive CD4 expression, followed by identification of $CD25^-$ Tresp cells.
c. Top: Representative flow cytometry plots of IFN-γ and TNF intracellular staining in tumor Tconv and 4 subsets of tumor Treg cells, following stimulation with PMA/ionomycin. Middle: Representative flow cytometry plots of IL-17 and IL-2 intracellular staining in tumor Tconv and 4 subsets of tumor Treg cells, following stimulation with PMA/ionomycin. Bottom: Mean % of cytokine producing cells for tumor Tconv and 4 subsets of tumor Treg cells, following stimulation with PMA/ionomycin, as shown above (n = 5 Tconv, 5 Treg intermediate, 3 quiescent, 5 effector, 5 $CD161^+$, depending on subset abundance and availability of sorted cells, patients with NSCLC, 3 independent experiments, lines indicate samples from the same donors). Two-tailed paired t-test. Numbers on graphs indicate p-value.
d. Pearson correlation graphs of the abundance of 4 Treg cell subsets (shown as % of total Treg cells) in n=159 patients with NSCLC used for RFS (recurrence-free survival) analysis in Fig. 2i.

For (c, d), source data are provided as a Source Data file.

SUPPLEMENTARY FIGURE 6. Flow cytometry analysis of CD4⁺ and Treg cells in patients with breast and lung cancer.

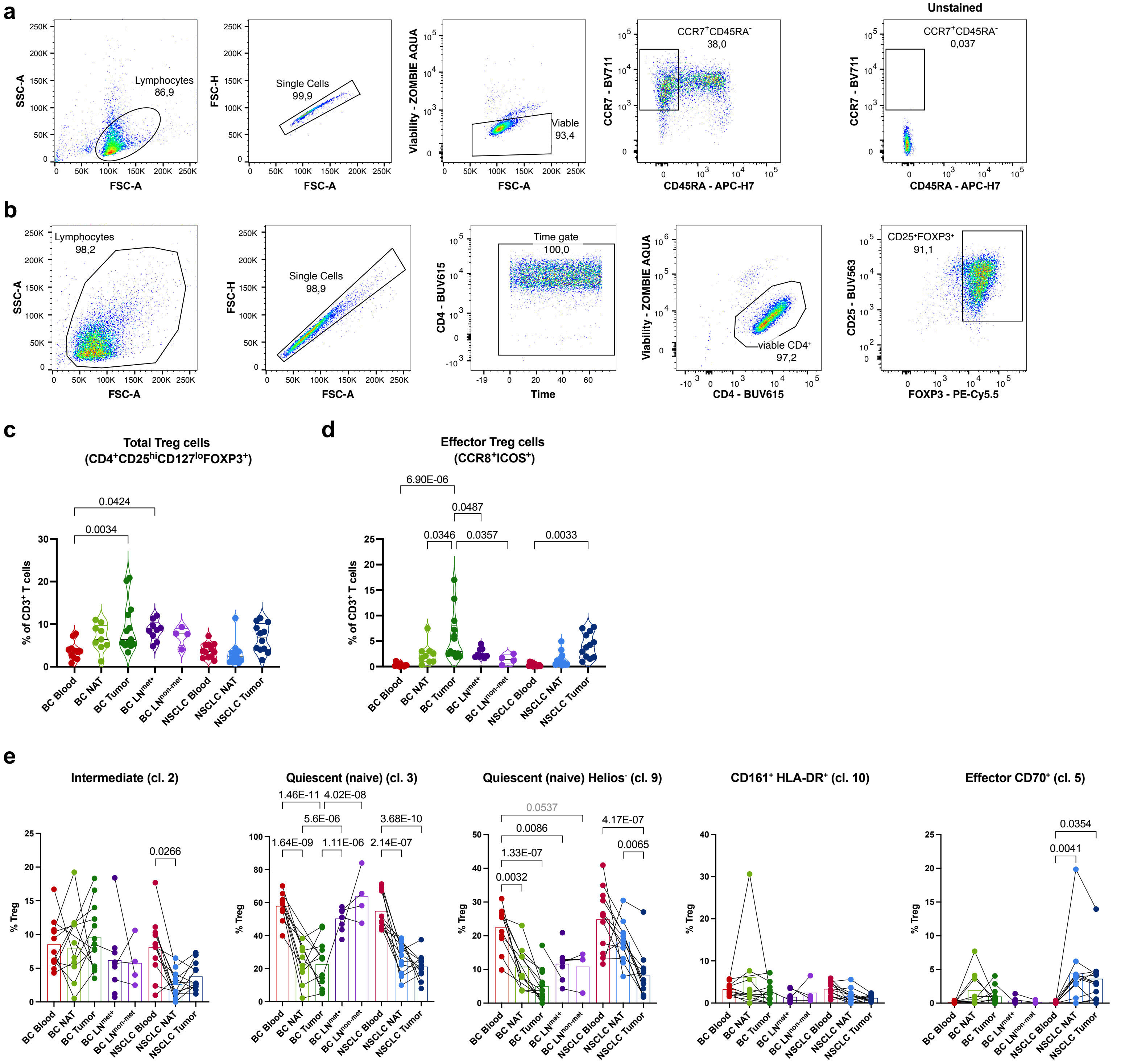

SUPPLEMENTARY FIGURE 6. Flow cytometry analysis of $CD4^+$ and Treg cells in patients with breast and lung cancer.

a. Representative flow cytometry sorting gating strategy, on a selected sample of Treg purified from healthy PBMCs, to obtain quiescent $CCR7^+$ Treg for *ex vivo* differentiation. Treg cells were first identified based on physical parameters and dead cells were excluded. Quiescent, non-naive cells were identified as $CCR7^+CD45RA^-$.
b. Representative flow cytometry gating strategy, on a selected sample of $CCR7^+CD45RA^-$ Treg differentiated *ex vivo*. $CD4^+$ T cells were first identified based on physical parameters, dead cell exclusion and gated as $CD4^+$. Treg were further gated as $CD25^{hi}FOXP3^+$.
c. Frequency of total Treg cells (by manual gating), among $CD3^+$ T cells, according to tumor type and tissue.
d. Frequency of $CCR8^+ICOS^+$ effector Treg cells (by manual gating), among $CD3^+$ T cells, according to tumor type and tissue. For (c) and (d), data is shown as violin plot with mean and quartiles marked and single data points represent samples from separate patients (n=11 BC blood, 9 BC NAT, 13 BC tumor, 4 BC non-metastatic lymph node, 9 BC metastatic lymph node, 12 NSCLC blood, 12 NSCLC NAT, 12 NSCLC tumor samples). One-way ANOVA with Tukey's post-test. Numbers on graphs indicate p-value.
e. Mean frequency of selected Treg cell clusters, as identified in main text Fig. 4a-c, according to tumor type and tissue. Lines indicate samples from the same patients (n=11 BC blood, 9 BC NAT, 13 BC tumor, 4 BC non-metastatic lymph node, 9 BC metastatic lymph node, 12 NSCLC blood, 12 NSCLC NAT, 12 NSCLC tumor samples). One-way ANOVA with Tukey's post-test. Numbers on graphs indicate p-value.

For (c-e), source data are provided as a Source Data file.

SUPPLEMENTARY FIGURE 7. Clonal relationship of Treg cells in tumor and tissue.

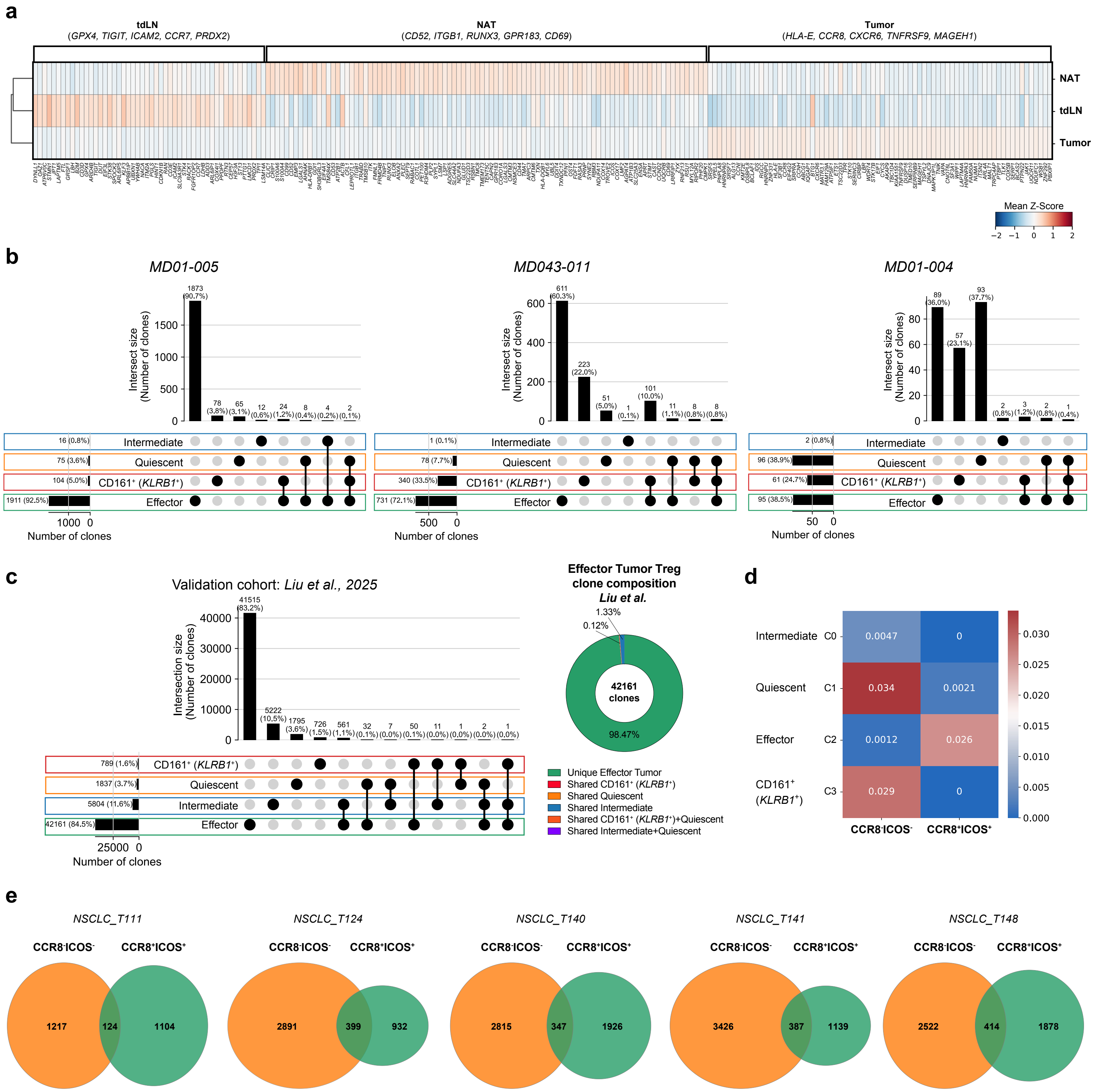

SUPPLEMENTARY FIGURE 7. Clonal relationship of Treg cells in tumor and tissue.

a. Heatmap showing relative gene expression of manually-curated genes of clonally related Treg cells residing in different tissues (tumor-draining LN, NAT, tumor), as identified in main text Fig. 5c.
b. Shared TCR clonotypes between the 4 subsets of Treg cells (Fig. 2a) inside the NSCLC tumor tissue. Data are expressed as in Fig. 5d, split separately for each patient. For (a-b) cells were analyzed from tumor, NAT and tumor-draining LN from n=3 patients with NCSLC.
c. Left: shared TCR clonotypes between the 4 subsets of Treg cells (Fig. 2a) inside the NSCLC tumor tissue, in a second cohort of patients with NSCLC from *Liu et al.*[5] (n=230 patients with NSCLC). Data are expressed as in Fig. 5d. Right: donut graph indicating composition of effector tumor Treg clones. Percentages of unique or clones shared with other Treg cell subsets are indicated.
d. Enrichment of transcriptomic signatures of 4 Treg subsets (identified in main text Fig. 2a) in bulk RNA sequencing of $CCR8^{+}ICOS^{+}$ and $CCR8^{-}ICOS^{-}$ Treg sorted from lung tumors[6] (n=5 patients with NSCLC), represented as Jaccard distance scores.
e. Venn diagram showing overlap of detected TCR sequences reconstructed from bulk RNA sequencing of $CCR8^{+}ICOS^{+}$ and $CCR8^{-}ICOS^{-}$ Treg cells sorted from n=5 patients with NCSLC, separately for every patient in the analyzed cohort (referring to main text Fig. 5e).

For (a-c, e), source data are provided as a Source Data file.

SUPPLEMENTARY FIGURE 8. Clonal relationship of Treg cells in tumor and tissue.

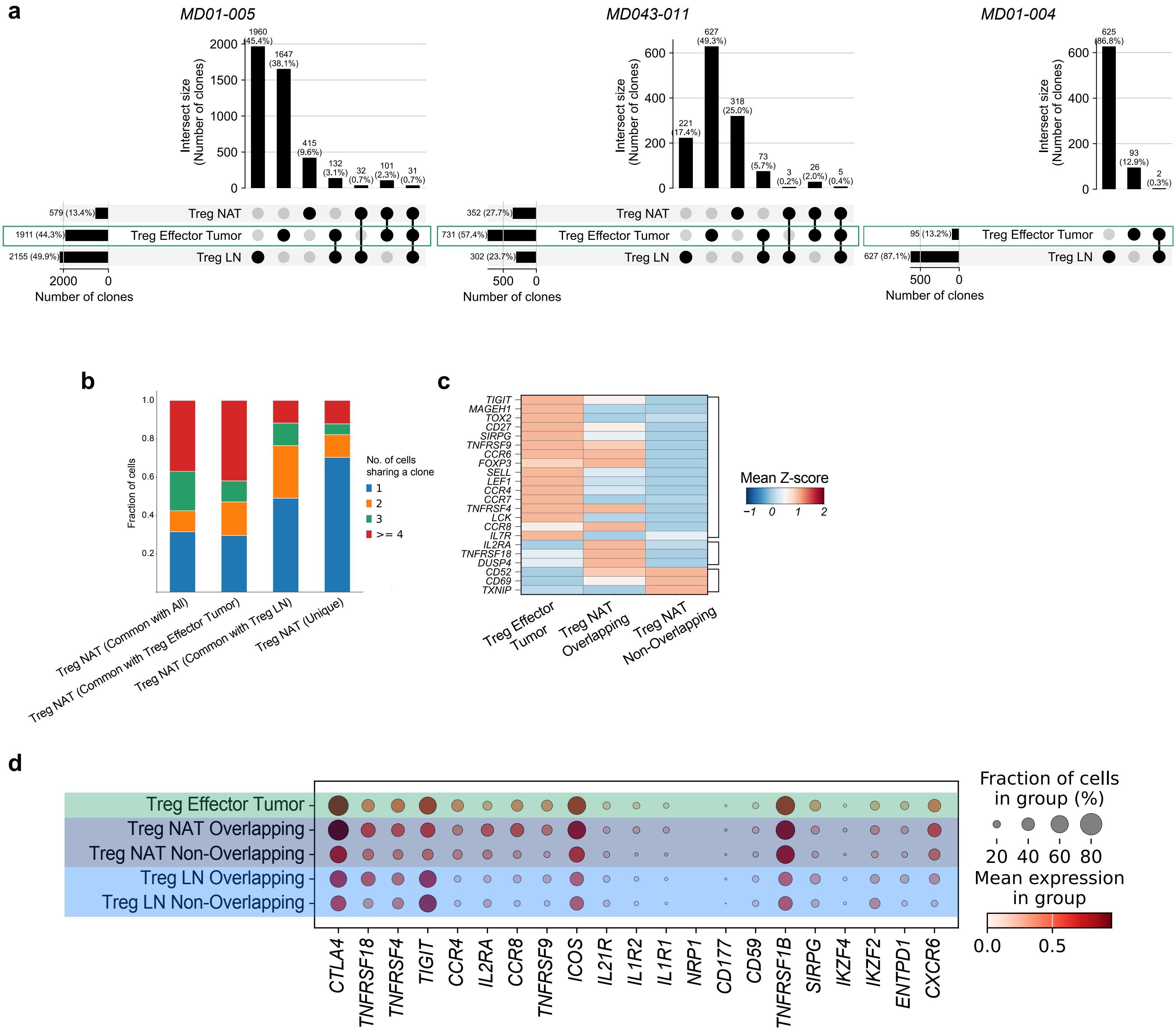

SUPPLEMENTARY FIGURE 8. Clonal relationship of Treg cells in tumor and tissue.

a. Shared TCR clonotypes between the effector Treg cells inside the NSCLC tumor tissue and total Treg cells from NAT and tumor-draining LN. Data are expressed as in Fig. 5f, split separately for each patient.
b. Number of Treg cells (1, 2, 3 or ≥4) bearing the same TCR clone (expressed in frequency, %) in NAT, depending on their overlap with effector tumor Treg and/or tumor-draining LN Treg cells. Higher number of cells sharing the same clone indicates clonal expansion.
c. Heatmap showing relative gene expression of manually-curated differentially expressed genes (FDR<0.05; expressed in ≥20% of cells, Two-sided Wilcoxon rank-sum test, Bonferroni correction) of effector tumor and clonally overlapping/non-overlapping NAT Treg cells, as identified in Supplementary Data 12.
d. Dot plot showing the average expression levels and expression frequencies of selected genes in effector tumor Treg cells and Treg cells in tumor-draining LN and NAT, depending on overlap with effector tumor Treg. Colors indicate cells residing in different tissues. For (a-d), cells were analyzed from tumor, NAT and tumor-draining LN from n=3 patients with NCSLC.

For (a-d), source data are provided as a Source Data file.

SUPPLEMENTARY FIGURE 9. Clonal relationship of effector Treg and Tconv cells.

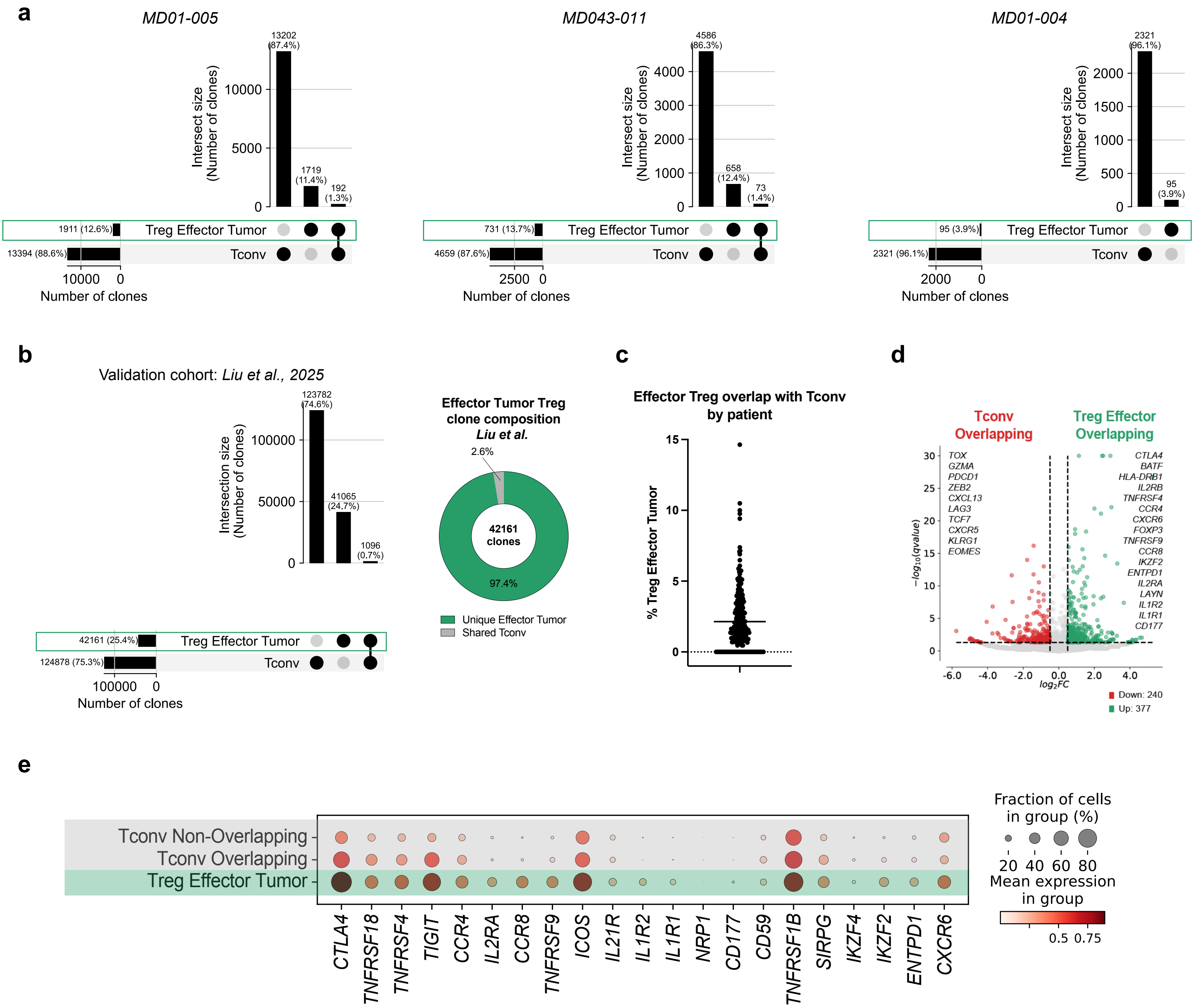

SUPPLEMENTARY FIGURE 9. Clonal relationship of effector Treg and Tconv cells.

a. Shared TCR clonotypes between the effector Treg cells inside the NSCLC tumor tissue and total conventional $CD4^+$ T cells (Tconv) from tumor, NAT and tumor-draining LN. Data are expressed as in Fig. 6a, split separately for each patient.
b. Left: shared TCR clonotypes between the effector Treg cells and conventional $CD4^+$ T cells (Tconv) from tumor NSCLC tumor tissue, in a second cohort of patients with NSCLC from *Liu et al.*[5] (n=230 patients with NSCLC). Data are expressed as in Fig. 6a. Right: donut graph indicating the composition of effector tumor Treg clones. Percentages of unique or Tconv-shared clones are indicated.
c. Mean % of effector tumor Treg cells with TCR overlapping with $CD4^+$ Tconv cells, for the data shown in b. Single data points for each patient are shown.
d. Volcano plot of differentially expressed genes between overlapping $CD4^+$ Tconv and overlapping effector tumor Treg, from Fig. 6a. Genes with LFC>0.5 and FDR<0.05 (Two-sided Wald test, Benjamini–Hochberg correction) were considered significant. Selected DEGs of interest are indicated.
e. Dot plot showing the average expression levels and expression frequencies of selected genes in effector tumor Treg cells and Tconv $CD4^+$ T cells, depending on overlap with effector tumor Treg. Colors indicate either Treg or Tconv cells. For (a, d, e) cells were analyzed from tumor, NAT and tumor-draining LN from n=3 patients with NCSLC.

For (a-e), source data are provided as a Source Data file.

Supplementary References

1. Poon, M.M.L. *et al.* Tissue adaptation and clonal segregation of human memory T cells in barrier sites. *Nat Immunol* **24**, 309-319 (2023).

2. Tabula Sapiens, C. *et al.* The Tabula Sapiens: A multiple-organ, single-cell transcriptomic atlas of humans. *Science* **376**, eabl4896 (2022).

3. Thomas, M.F. *et al.* Single-cell transcriptomic analyses reveal distinct immune cell contributions to epithelial barrier dysfunction in checkpoint inhibitor colitis. *Nat Med* **30**, 1349-1362 (2024).

4. Caushi, J.X. *et al.* Transcriptional programs of neoantigen-specific TIL in anti-PD-1-treated lung cancers. *Nature* **596**, 126-132 (2021).

5. Liu, Z. *et al.* A single-cell atlas reveals immune heterogeneity in anti-PD-1-treated non-small cell lung cancer. *Cell* **188**, 3081-3096 e3019 (2025).

6. Alvisi, G. *et al.* IRF4 instructs effector Treg differentiation and immune suppression in human cancer. *J Clin Invest* **130**, 3137-3150 (2020).
