# Supplementary material for: Molecular heterogeneity and clonal origin of CCR8+ effector regulatory T cells in human cancer
Source: Nat Commun. 2026 Aug 21;17:9994. doi: 10.1038/s41467-026-76670-6 (PMC13590533; doi:10.1038/s41467-026-76670-6)
Supplement: Supplementary file 4 [file 41467_2026_76670_MOESM2_ESM.pdf]

Description of Additional Supplementary Files

Supplementary Data 1. Gene expression in clusters of $CD4^+$ T cells in the integrated dataset, for each cluster complete list of genes and DEGs filtered for FDR<0.05, LFC>1 (Two-sided Wilcoxon rank-sum test, Bonferroni correction).

Supplementary Data 2. Gene expression in tumor Treg vs. tumor Tconv cells, for each tumor type in the analyzed dataset (Two-sided t-test_overstim_var, Bonferroni correction).

Supplementary Data 3. Genes upregulated in tumor Treg vs. tumor Tconv cells (FDR<0.05; expressed in at least 20% of cells), comparative for all tumor types in analyzed dataset.

Supplementary Data 4. Gene expression in C3 (Treg) cluster vs. each of $CD4^+$ Tconv clusters, all genes compared and results for 88 core Treg signature genes (Two-sided Wilcoxon rank-sum test, Bonferroni correction).

Supplementary Data 5. Gene expression in tumor Treg vs. NAT Treg cells, for each tumor type in the analyzed dataset (Two-sided t-test_overstim_var, Bonferroni correction).

Supplementary Data 6. Gene set enrichment analysis (REACTOME collection) of Treg cell upregulated genes specific for certain tumors (One-sided hypergeometric test, g:SCS correction).

Supplentary Data 7. Gene expression in 4 clusters of Treg cells in the integrated datset, for each cluster complete list of genes and DEGs filtered for FDR<0.05, LFC>0, PCT>0.2 (Two-sided Wilcoxon rank-sum test, Bonferroni correction).

Supplementary Data 8. Antibodies and reagents used for flow cytometry experiments.

Supplementary Data 9. Differential gene expression in effector (C2) tumor Treg between breast and lung tumors (Two-sided Wilcoxon rank-sum test, Bonferroni correction).

Supplementary Data 10. Gene expression in clonally related Treg cells residing in different tissues (tumor-draining LN, NAT, tumor) (Two-sided Wilcoxon rank-sum test, Bonferroni correction).

Supplementary Data 11. Gene expression analysis between effector tumor Treg and Treg cells in the tumor-draining LN, filtered for adj p-value<0.05, PCT>0.2, positive LFC (Two-sided Wilcoxon rank-sum test, Bonferroni correction).

Supplementary Data 12. Gene expression analysis between NAT Treg clonally non-overalapping with effector tumor Treg and effector tumor Treg+overlapping NAT Treg cells, filtered for adj p-value<0.05, PCT>0.2, positive LFC (Two-sided Wilcoxon rank-sum test, Bonferroni correction).

Supplementary Data 13. Gene expression analysis between Tconv cells, depending on their overlap with intratumoral effector Treg. Positive LFC indicates higher expression in cells overlapping with effector Treg cells in tumor (Two-sided Wald test, Benjamini–Hochberg correction).

Supplementary Data 14. Gene expression analysis between Tconv cells overlapping with intratumoral effector Treg and intratumoral effector Treg, overlapping with Tconv cells. Positive LFC indicates higher expression in effector Treg cells in tumor (Two-sided Wald test, Benjamini–Hochberg correction).
